# Metal-mediated weak adsorption enables the selective electrosynthesis of *N*-phenylhydroxylamine from nitrobenzene hydrogenation in water

**DOI:** 10.1038/s41467-026-76066-6

**Published:** 2026-07-30

**Authors:** Zhaole Lu, Rong Yang, Renxiang Cheng, Lingjun Kong, Bin Zhang

**Affiliations:** Institute of Molecular Plus, Department of Chemistry, School of Science, Tianjin University, Tianjin, China

**Keywords:** Electrocatalysis, Sustainability

## Abstract

The electrocatalytic hydrogenation of nitrobenzene (Ph-NO_2_) to *N*-phenylhydroxylamine (Ph-NHOH) is of industrial importance as a high-value-added process but remains challenging because of the thermodynamically favorable overhydrogenation process. Here, metal-mediated weak adsorption is proposed to enable selective Ph-NO_2_-to-Ph-NHOH electrosynthesis in water. An activity volcano in the context of scaling relationships based on the adsorption energy of Ph-NO has identified metallic silver (Ag) as the optimal highly intrinsically active candidate for Ph-NHOH electrosynthesis. The results of in situ electrochemical infrared spectroscopy reveal the weak adsorption of Ph-NHOH on metallic Ag, which prevents its subsequent hydrogenation. Afterward, a nanosized Ag catalyst (CV-Ag) synthesized by electrodeposition is developed to rapidly and selectively synthesize Ph-NHOH under a large current. CV-Ag enhances the electrosynthesis reaction kinetics of Ph-NHOH. Therefore, an ampere-level current is realized during the electrosynthesis of Ph-NHOH, achieving a high selectivity of nearly 99.6% and continuous gram-scale synthesis of Ph-NHOH at 5.85 g per batch.

## Abstract (web summary)

The sustainable synthesis of N-phenylhydroxylamine (Ph-NHOH), a high-value intermediate, without over-hydrogenation side reactions is highly desirable. Here, the authors report the electrosynthesis of Ph-NHOH from nitrobenzene on metallic silver catalysts in aqueous media under ambient conditions.

## Introduction

N-Phenylhydroxylamine (Ph-NHOH) is an important synthetic intermediate that is extensively applied in pharmaceutical manufacturing and synthetic chemistry as a key building block for numerous pharmacologically active molecules, such as *o*-acetylisonipecarbin^1–3^. Its utility as a fundamental intermediate in the synthesis of sulfonamides and nitroimidazoles further highlights its industrial importance^4,5^. At present, the inherent chemical instability of the benzene ring in Ph-NHOH may lead to undesirable side reactions, including isomerization, branching, and oxidation, which decrease the selectivity of the reaction^6^. A key obstacle is that thermodynamic driving forces favor overhydrogenation of the intermediate Ph-NHOH to phenylamine (Ph-NH_2_), which reduces the selectivity^7–10^. Consequently, achieving highly selective Ph-NHOH synthesis remains a great challenge.

Conventional synthesis routes predominantly rely on the reduction of zinc powder in high-concentration ammonia chloride, catalytic hydrogenation, and sodium borohydride reduction, but these approaches suffer from excessive energy consumption, operationally hazardous hydrogen sources, and stringent reaction conditions, which pose both economic and environmental sustainability challenges^11–16^. Alternatively, electrochemical reduction offers a more sustainable alternative, a strategy that has been explored since early studies on the electroreduction of nitroarenes^17,18^. Recent advancements in selective Ph-NHOH synthesis have been achieved through distinct strategies, including catalyst engineering and reactor design^19^. A representative example of the former is an interfacial electronic effect induced by ethylenediamine modification to control the catalytic selectivity of a well-designed Pt-based catalyst, which has achieved high selectivity for the synthesis of nitrobenzene (Ph-NO_2_) to Ph-NHOH^20^. However, a major concern with the introduction of organic modifiers is not only the potential masking of active sites but also their instability during the reaction process. Therefore, the development of green synthesis methods that are capable of enabling highly selective Ph-NHOH synthesis by enhancing the intrinsic activity of catalysts is highly desirable.

In this article, we propose a mild metal-mediated electrochemical method for Ph-NHOH synthesis using water as the hydrogen source, eliminating the need for conventional chemical reductants^21,22^. First, a thermodynamic analysis of the electroreduction of Ph-NO_2_ is performed to screen silver (Ag) as the optimal highly intrinsically active candidate for Ph-NHOH electrosynthesis. We experimentally determine that the performance of Ph-NHOH electrosynthesis from Ph-NO_2_ is closely related to the adsorption of Ph-NHOH on the metal catalyst surface. Furthermore, the optimized catalyst (CV-Ag) achieves a high Ph-NHOH selectivity of nearly 98.0% and a Faraday efficiency (FE) of 96.5%. The broad substrate scope, encompassing halogen, methyl, cyano, ester, aldehyde, and ketone substituents, demonstrates the versatility of this method. More importantly, the continuous and highly selective synthesis of Ph-NHOH at a current of 1.5 A is achieved in a zero-gap membrane electrode assembly electrolyzer, enabling the gram-scale synthesis of Ph-NHOH at 5.85 g per batch.

## Results

### Analysis of the calculation-assisted reaction process

**Fig. 1:**
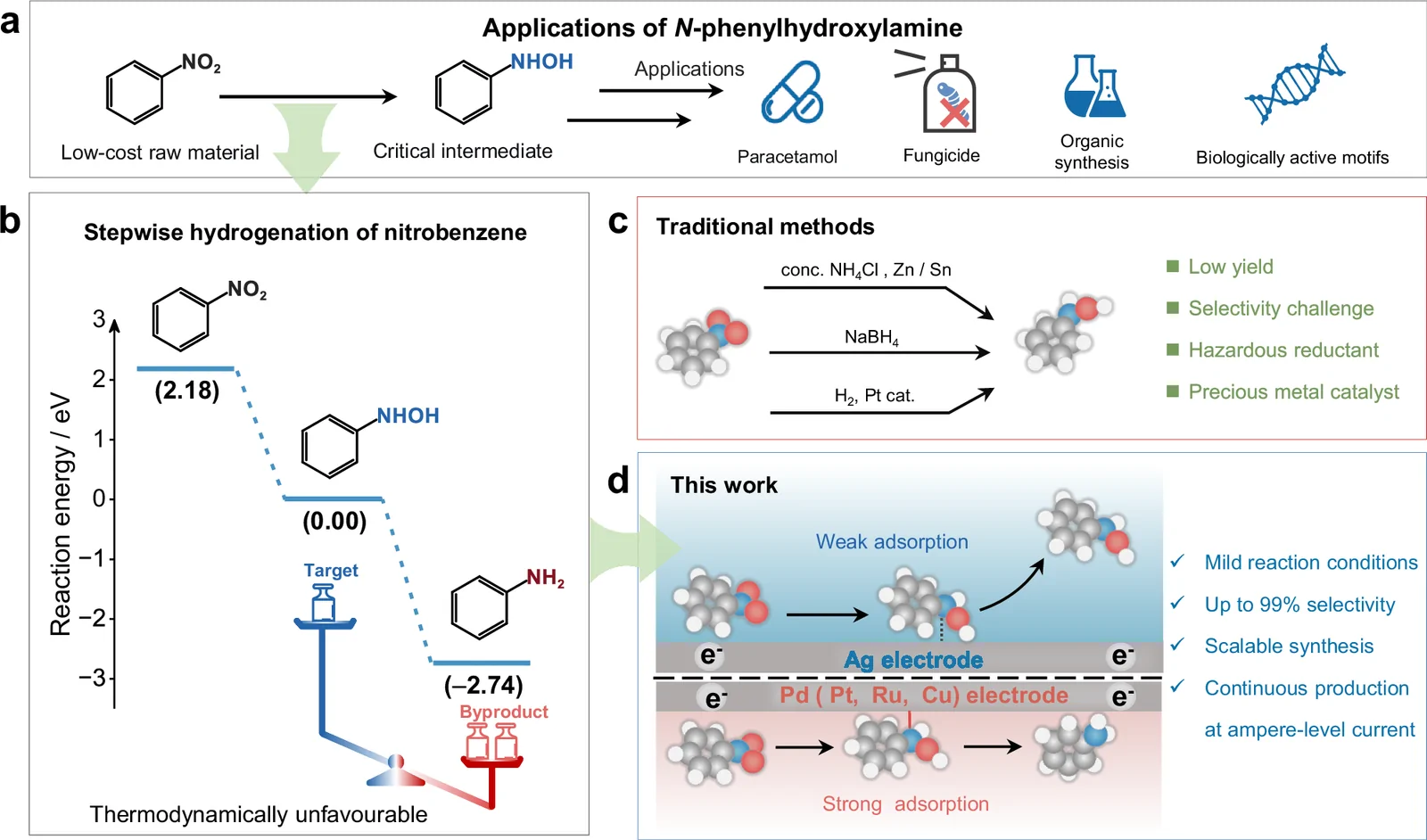
Applications, synthesis challenge and our proposed electrosynthetic method of Ph-NHOH. **a** Applications of Ph-NHOH. **b** Challenge of Ph-NO_2_-to-Ph-NHOH synthesis. **c** Industrial production of Ph-NHOH through multiple steps. **d** Ph-NO_2_-to-Ph-NHOH electrosynthesis over a CV-Ag catalyst. Source data for Fig. 1b are provided as a Source Data file.

The mechanism of the electrosynthesis of Ph-NO_2_-to-Ph-NHOH involves a two-step process and four-electron transfer (Supplementary Fig. 1)^23^. Analysing Ph-NO_2_-to-Ph-NHOH synthesis reveals that possible competitive reactions include the overhydrogenation of Ph-NHOH to Ph-NH_2_, the oxidation of Ph-NHOH to nitrosobenzene (Ph-NO), the condensation of Ph-NO with Ph-NHOH into azoxybenzene, and the hydrogen evolution reaction (HER)^24^. The formation of azoxybenzene can be avoided, and Ph-NHOH is stable in acidic solutions (Supplementary Fig. 2, Supplementary Note 2). Thus, density functional theory (DFT) calculations were performed to reveal a sequential hydrogenation pathway for Ph-NO_2_, proceeding through Ph-NO and Ph-NHOH to ultimately form Ph-NH_2_. The Gibbs free energy (Δ*G*) values for Ph-NO_2_-to-Ph-NHOH and Ph-NHOH-to-Ph-NH_2_ were calculated to be –2.18 and –2.74 eV, respectively (Fig. 1b). These results indicate that these steps are energetically advantageous and that Ph-NH_2_ is the most stable final product of the entire hydrogenation pathway. Thus, achieving high-efficiency and high-selectivity Ph-NHOH via electrochemical approaches is challenging.

**Fig. 2:**
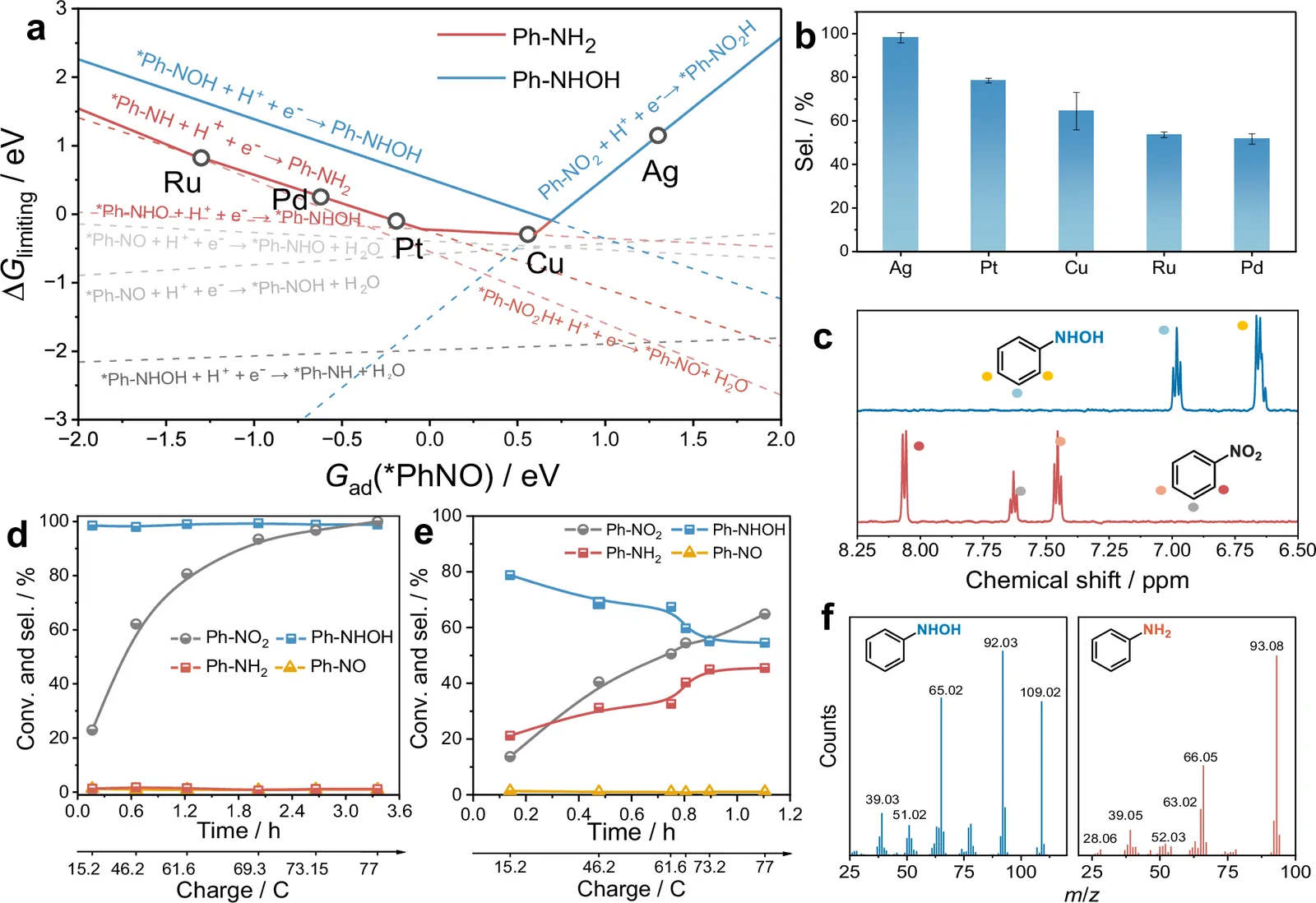
Ph-NO_2_ electroreduction over different metals. **a** *G*_ad_(*Ph-NO) plotted against ΔG_limiting_ over different metal cathodes from DFT calculations. **b** Comparison of Ph-NHOH selectivity over five metal electrodes at –0.2 V vs. RHE. **c** ^1^H NMR spectra of Ph-NHOH and Ph-NO_2_ from the Ph-NO_2_ hydrogenation reaction. **d**,** e** Time-dependent catalysis over the Ag electrode and Pd electrode. **f** MS spectra of the obtained Ph-NHOH and Ph-NH_2_. Source data for Fig. 2 are provided as a Source Data file.

Thermodynamics determine the directionality of a reaction and are crucial for investigating product selectivity^25^. Regulating the adsorption energy of key intermediates on the catalyst surface to promote the rapid desorption of Ph-NHOH after its formation, thereby preventing its further, thermodynamically more favourable hydrogenation, is expected to ultimately reverse the reaction selectivity toward the formation of Ph-NHOH^26^. Considering the stability of the electrode under acidic conditions, five possible active commercial metals, namely, Cu, Ag, Pd, Pt, and Ru, were examined and compared. We calculated the adsorption energies of all possible intermediates during the use of Ph-NO_2_ to produce Ph-NHOH and Ph-NH_2_ (Supplementary Fig. 3, Supplementary Data 1)^27^. The adsorption energy of *Ph-NO [*G*_ad_(Ph-NO)] was chosen as the descriptor to establish a correlation between different adsorbates and these metal catalysts (Supplementary Fig. 4, Supplementary Note 3). The Δ*G* values of all possible elementary steps were calculated and plotted versus the adsorption free energy of *Ph-NO to establish an activity volcano based on scaling relations, where the solid blue and solid red lines represent the Δ*G*-determining steps (Δ*G*_limiting_) of Ph-NHOH and Ph-NH_2_ production, respectively (Fig. 2a, Supplementary Tables 1, 2). We predicted the selectivity of each metal catalyst and labeled each catalyst species. On the strong binding side of the volcano plot, Ph-NHOH cannot outcompete Ph-NH₂ as the dominant product. In contrast, Ph-NHOH formation became feasible on the weak binding side of the plot. As a result, compared with metallic Ag, Ph-NHOH is more thermodynamically preferred over Ph-NH_2_.

Given that the pH, temperature, and diffusion layer critically influence the reaction kinetics and mass transport, these parameters were fixed to ensure a fair comparison of the relative catalytic activities of the different bulk metal electrodes. Electrochemical reduction tests of Ph-NO_2_ were first performed over five bulk metal electrodes in a 0.5 M phosphate solution (pH = 5.8) at room temperature using an H-type cell at 800 rpm. By comparing the cyclic voltammetry (CV) results for different electrodes, reduction peaks were observed for five metals after the addition of Ph-NO_2_, indicating that all five metals possess sufficient activity to reduce Ph-NO_2_ (Supplementary Fig. 5)^22^. The potential range for producing Ph-NHOH on the metallic Ag and Cu electrodes is wider than that of the other bulk metal electrodes. Additionally, the Ag electrode has the lowest onset potential of approximately 0.28 V for Ph-NO_2_ reduction, indicating that Ph-NO_2_ is most readily reduced on the Ag electrode. A cathodic peak appeared at 0.2 V because of the reduction of Ph-NO_2_ to Ph-NHOH^28^. An anodic peak appears in the return cycle at 0.25 V, which is coupled with the cathodic peak at 0.29 V, which represents the two-electron reversible transformation between Ph-NHOH and Ph-NO^29^.

We compared the performance of Ph-NHOH electrosynthesis over these metal electrodes, and the aqueous products were detected after electrolysis at a constant potential (Fig. 2b). ^1^H nuclear magnetic resonance (NMR) spectroscopy and liquid chromatography‒mass spectrometry (LC‒MS) were used to qualitatively analyse the condensation products in the electrolyte. After electrolysis, the characteristic peaks of Ph-NO_2_ at 8.06, 7.63, and 7.46 ppm in the ^1^H NMR spectra disappeared, and the characteristic peaks of Ph-NHOH appeared at 7.14 and 6.85 ppm (Fig. 2c)^30^. The peaks at a mass-to-charge ratio (m/z) of 109.02 and a m/z of 93.08 in the mass spectra demonstrate the formation of Ph-NHOH and Ph-NH_2_, respectively (Fig. 2d). These results demonstrate that accurate determination of the production of Ph-NHOH is feasible. On the basis of the LC data and ^1^H NMR data, the Faraday efficiency (FE), selectivity, and yield rate were calculated from the relevant calibration curves (Supplementary Figs. 6−9). The results showed that the Ag electrode exhibited the highest Ph-NHOH selectivity among these metal electrodes, which is up to 98.1% at –0.2 V vs. RHE (Fig. 2b). In contrast, the Ph-NHOH selectivities of all the other metals are less than 80.0%. In particular, the metallic Pd electrode demonstrates the lowest selectivity for Ph-NHOH (51.7%). Throughout the electrocatalysis process, Ph-NH_2_ is the main aqueous product (Supplementary Fig. 10).

Furthermore, the time-dependent catalysis over the Ag electrode and the Pd electrode was compared. The reaction was terminated when the charge reached 77 C according to the complete transfer of four electrons. After 2 h, the conversion rate of Ph-NO_2_ over the Ag electrode reaches 90%, indicating that the Ag electrode improved the hydrogenation kinetics of Ph-NO_2_. Moreover, no significant decay, i.e., no significant hydrogenation from Ph-NHOH to Ph-NH_2_, is observed even when the reaction time is prolonged to 2 h. The Pd electrode initially exhibits a Ph-NHOH selectivity of 80.0%. (Fig. 2e, f). As Ph-NO_2_ is consumed, the selectivity of Ph-NHOH continuously decreases with increasing Ph-NH_2_. When the reaction charge is exhausted, the selectivity of Ph-NHOH is less than 55.0%. The above calculations and experimental results suggest that Ag is a promising metal catalyst for highly selective Ph-NHOH synthesis that can inhibit the overhydrogenation of Ph-NHOH to Ph-NH_2_.

**Fig. 3:**
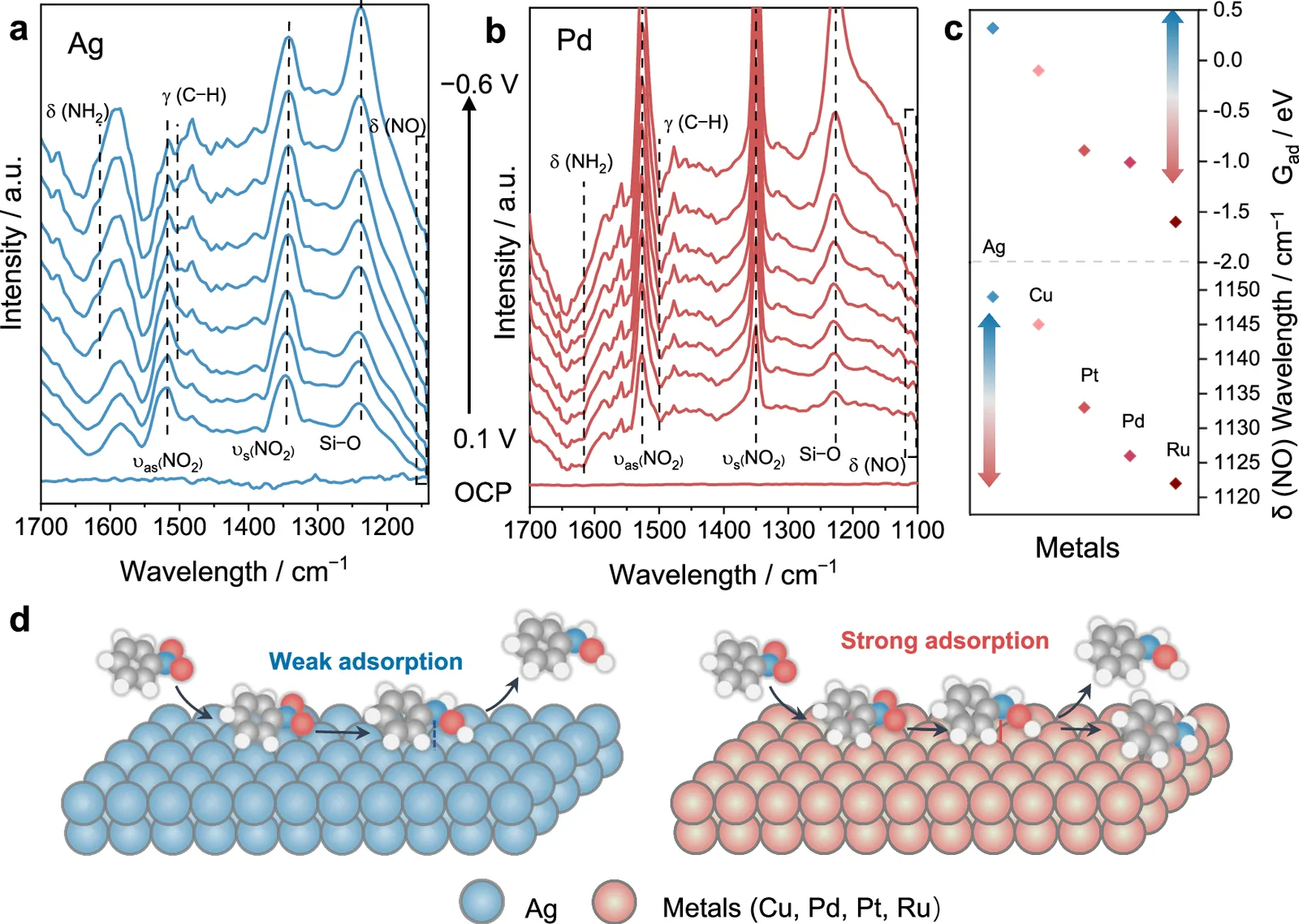
Mechanistic insights from different metals. **a**,** b** In situ ATR-FTIR spectra under various bias potentials over Ag and Pd (Y-axis: transmittance). **c** Corresponding wavenumbers of the N–O stretching vibration related to Ph-NHOH and *G*_ad_ (Ph-NHOH) over five metals. **d** Proposed possible mechanism for different bulk metal catalysts. Source data for Fig. 3a–c are provided as a Source Data file.

To understand how the five different metallic electrodes affect Ph-NHOH selectivity, electrochemical in situ attenuated total reflectance Fourier transform infrared (ATR-FTIR) spectroscopy was employed to characterize the reaction process^31^. The absorption bands at approximately 1352 and 1523 cm^−1^ in the ATR-FTIR spectra of the five metals are assigned to the symmetric and asymmetric stretching vibrations of the nitro group, *ν*_s_(NO_2_) and *ν*_as_(NO_2_), in Ph-NO_2_ (Fig. 3a, b, Supplementary Fig. 11)^32^, indicating that Ph-NO_2_ is adsorbed onto the metal electrode surface during the electrochemical process. Additionally, the N‒H bending vibration peak at 1622 cm^−1^ indicates the generation of Ph-NH_2_^33^. The peak near 1120 to 1145 cm^−1^ was related to the stretching vibration of N−O [δ(NO)] in Ph-NHOH, as reported previously^34,35^. The wavenumbers of *δ*(NO) on these metal electrodes clearly differ, following the trend Ag > Cu > Pt > Pd > Ru, which indicates that the adsorption strength of Ph-NHOH follows the order Ag < Cu < Pt < Pd < Ru. Additionally, the Ph-NHOH adsorption energies (*G*_ad_ [Ph-NHOH]) of the five metals obtained from the DFT calculations tend to be Ag > Cu > Pt > Pd > Ru, which is fully consistent with the IR results. Thus, the Ag electrode has the weakest adsorption strength for Ph-NHOH, which is perfectly consistent with the calculation results (Fig. 3c)^36^.

To further investigate the reaction process, linear sweep voltammetry (LSV) was conducted under different pH conditions (Supplementary Fig. 12, Supplementary Note 4). The corresponding reduction potentials as a function of pH indicate that the potentials of the reduction peaks for Ph-NO, Ph-NHOH, and Ph-NH_2_ on Ag all shift by approximately 59 mV per unit of pH, which is consistent with a proton-coupled electron transfer mechanism^37^. In the Ph-NO reduction to Ph-NHOH stage, all five metals exhibit Tafel slopes of approximately 120 mV dec^−1^ (Supplementary Fig. 13a, b), indicating that the reaction kinetics are controlled by an electron transfer process^38^. Cyclic voltammetry (CV) experiments were further performed in aprotic anhydrous acetonitrile containing Ph-NO to directly assess the intrinsic electron transfer capabilities of the five metals (Supplementary Fig. 14a, Supplementary Note 5). The peak-potential difference (Δ*E*_P_) for Ph-NO reduction varied significantly across the different metal electrodes (Supplementary Fig. 14b), revealing the electron transfer capabilities of the metal electrodes^39^. Although Ag and Ru also possess good electron transfer capabilities, the weaker adsorption of Ph-NHOH on Ag prevents overhydrogenation of the intermediate. In the critical stage of Ph-NHOH reduction to Ph-NH_2_, the kinetic isotope effect (KIE) values for all five metals exceeded 1.5 (Supplementary Fig. 15a–e), confirming that proton transfer is involved in the rate-determining step^40,41^. Therefore, the reaction selectivity is fundamentally governed by the weak adsorption characteristics of the key Ph-NHOH intermediate rather than the kinetic matching of electron/proton transfer. These results emphasize the significant role of weak Ph-NHOH adsorption in Ph-NHOH electrosynthesis (Fig. 3d).

### Structural optimization of the Ag catalyst for Ph-NHOH synthesis

**Fig. 4:**
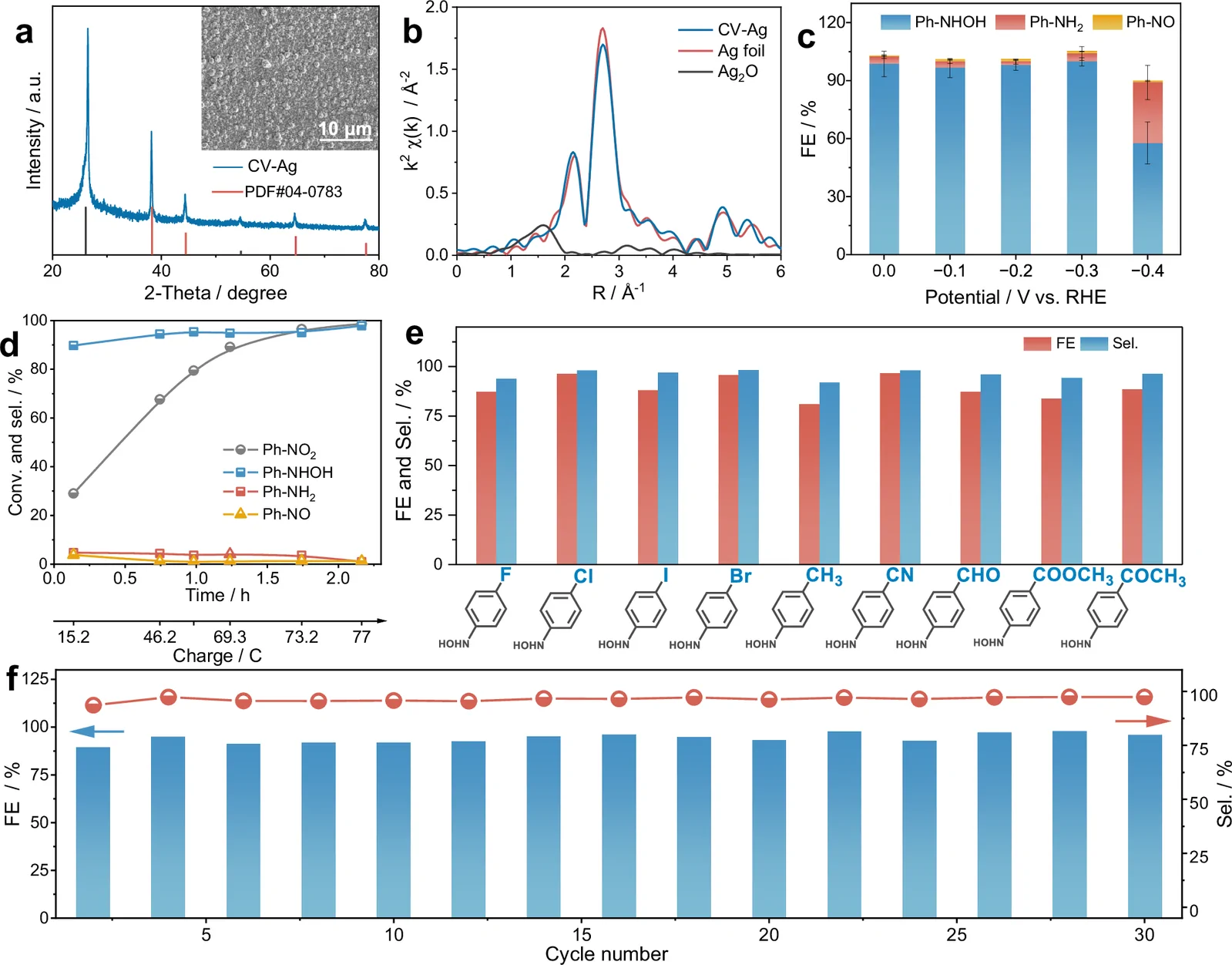
Characterizations and performance of CV-Ag. **a** XRD pattern of CV-Ag, with the corresponding SEM image shown in the inset. **b** EXAFS spectra of CV-Ag, Ag foil, and Ag_2_O. **c** Potential-dependent product distributions over CV-Ag. **d** Time-dependent catalysis over CV-Ag. **e** Synthesis of functionalized *N*-phenylhydroxylamine derivatives over CV-Ag. **f** Electrochemical cycling stability of the CV-Ag electrode. Source data for Fig. 4 are provided as a Source Data file.

To accelerate Ph-NHOH synthesis, an optimal Ag catalyst (CV-Ag) was prepared via cyclic voltammetry (CV) electrodeposition. The greater accessibility of active sites over CV-Ag than bulk Ag made it possible to achieve a high yield rate of Ph-NHOH (Supplementary Fig. 16). Scanning electron microscopy (SEM) images reveal that the surface of the CV-Ag electrode is rough, with uniformly distributed Ag nanoparticles averaging 68 nm in diameter (Fig. 4a, Supplementary Fig. 17). High-resolution transmission electron microscopy (HRTEM) characterization and corresponding selected area electron diffraction images confirmed the crystal lattice in CV-Ag (Supplementary Fig. 18). Smaller grains and more abundant grain boundaries were observed in CV-Ag, indicating the formation of more active sites.

The crystal phase and surface chemical states of CV-Ag were confirmed via X-ray diffraction (XRD) and X-ray photoelectron spectroscopy (XPS) (Fig. 4a, Supplementary Fig. 19). The XRD patterns demonstrated the presence of the monoconstituent metallic Ag in CV-Ag. The Ag 3d_3/2_ peak at 374.6 eV and the Ag 3d_5/2_ peak at 368.6 eV are observed in the XPS spectra, which correspond to the formation of Ag^0^, which is consistent with the XRD patterns^42^. The local coordination environment of the as-obtained CV-Ag was investigated via *k*^2^-weighted Fourier transformed extended X-ray absorption fine structure (EXAFS) spectroscopy (Fig. 4b). The EXAFS spectrum of CV-Ag exhibited a Ag–Ag scattering path at 2.86 Å, which is consistent with that of metallic Ag foil, whereas the absence of a Ag–O peak confirms complete reduction to the metallic state^43,44^. The similar peak of the Ag–Ag bond possibly suggests that the coordination structures between CV-Ag and the Ag foil are the same (Supplementary Figs. 20−21). Quantitative EXAFS fitting reveals that the average coordination number (CN) of Ag in CV-Ag is approximately 10.7, which is slightly lower than that of Ag foil (12.0) because of the effect of particle size. (Supplementary Table 3, Supplementary Note 12).

The electrochemical behavior of CV-Ag was first investigated via linear sweep voltammetry (LSV) at a scan rate of 10 mV s^−1^. As illustrated in Supplementary Fig. 22, the addition of Ph-NO_2_ induces a positive shift in the onset potential from −0.32 V to–0.28 V, indicating the occurrence of an electroreduction reaction of Ph-NO_2_. The reduction current peak near 0.15 V is ascribed to the reduction of *Ph-NO_2_ to *Ph-NHOH^45^. Furthermore, the enhanced Ph-NHOH selectivity and FE on CV-Ag are confirmed. After the pH of the electrolyte is optimized, over the potential range from 0 V to −0.3 V vs. RHE, CV-Ag delivers a high selectivity of 98.0% and a Ph-NHOH FE of 96.5% (Fig. 4c, Supplementary Figs. 23, 24 and Supplementary Note 6). The time-dependent catalysis demonstrates that the conversion of Ph-NO_2_ over CV-Ag reaches 98.4% after 2 h (Fig. 4d). Additionally, Ph-NHOH is produced preferentially over CV-Ag, with nearly 94.0% selectivity within 40 min. When the charge reaches 77 °C, the conversion time of Ph-NO_2_ over CV-Ag is less than that over the bulk Ag electrode. These results indicate that optimal CV-Ag maintains high Ph-NHOH selectivity and improves the hydrogenation kinetics of Ph-NO_2_. This is mainly due to the substantially enlarged electrochemically active surface area of the CV-Ag catalyst (Supplementary Fig. 25, Supplementary Note 7)^46^. Functional group compatibility analyses reveal that the reaction is compatible with the tested substrates bearing halogens, methyl, cyanogen, esters, aldehydes, and ketone groups (Fig. 4e, Supplementary Figs. 26–34). Various phenylhydroxylamine derivatives with considerably high FEs and selectivities are obtained, confirming the wide adaptability of this electrosynthesis method (Supplementary Table 4). Furthermore, no decrease in selectivity or FE was observed for Ph-NHOH after 30 cycles (Fig. 4f). XPS spectra suggest the excellent durability of the CV-Ag cathode for this selective Ph-NHOH electrosynthesis (Supplementary Fig. 35).

### Ampere-level scalable electrosynthesis of Ph-NHOH

**Fig. 5:**
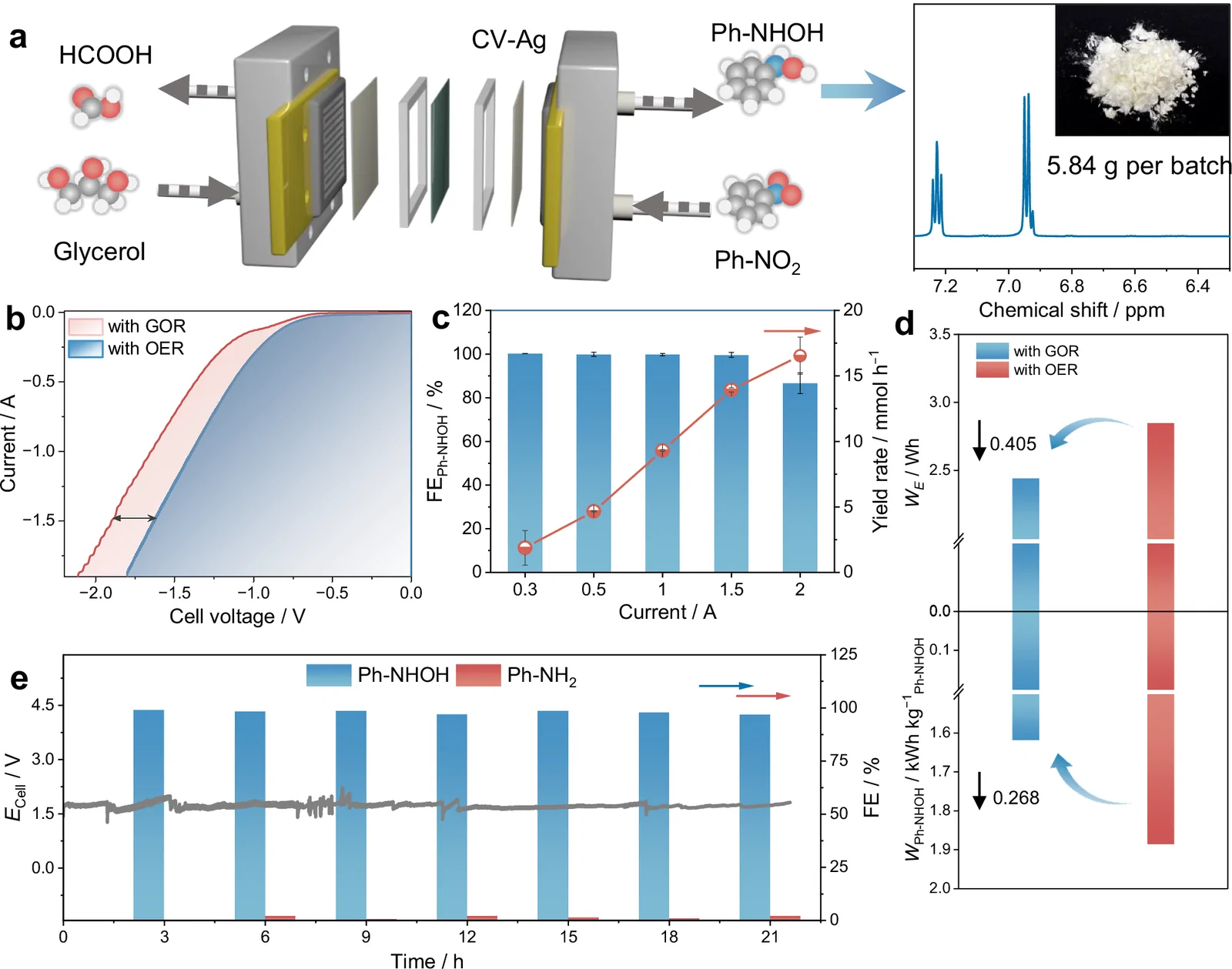
Ampere-level Ph-NHOH electrosynthesis. **a** Schematic diagram of the internal structure of the MEA electrolyzer, and ^1^H NMR and a photograph of the as-prepared Ph-NHOH. **b** LSV curves over the CV-Ag catalyst with or without GOR. **c** Ph-NHOH FE and yield rate at different currents. **d** The values of the *W*_E_ and *W*_Ph-NHOH_ during the Ph-NHOH electrosynthesis process with or without the GOR over the CV-Ag catalyst. **e** Time-dependent voltage curve and FEs over CV-Ag at 1.5 A. Source data for Fig. 5 are provided as a Source Data file.

Given its optimal performance, CV-Ag was employed for the large-scale synthesis of Ph-NHOH, which is proven to have high added value (Supplementary Fig. 36, Supplementary Note 8). To enable scalable production, a zero-gap membrane electrode assembly (MEA) electrolyzer was designed using CV-Ag as the cathodic catalyst and Ni foam as the anodic catalyst under conditions that mimicked those of practical processes (Fig. 5a, Supplementary Fig. 37). The Ph-NO_2_ concentration was optimized without compromising the selective performance (Supplementary Fig. 38). The glycerol electrooxidation reaction (GOR) was used as a high-value-added, low-cell-voltage anodic reaction to replace the oxygen evolution reaction (OER) (Supplementary Fig. 39, Supplementary Note 9)^47,48^. The introduction of glycerol shifts the polarization curves by 0.23 V at 1.5 A compared with the OER, corresponding to a positive shift in the cathodic potential from –1.85 V to –1.62 V (Fig. 5b). When a partial current was applied up to 1.5 A, a high Ph-NHOH FE of 99.6% was maintained, with a high Ph-NHOH yield rate of 13.9 mmol h^−1^, which is competitive with reported systems (Fig. 5c, Supplementary Table 5).

An evaluation was further conducted on the consumed electricity (*W*_E_) necessary to reach 1.5 A and the energy required to produce 1.0 kg of Ph-NHOH (*W*_Ph-NHOH_). As presented in Fig. 5d, approximately 2.44 Wh of electricity is consumed for GOR coupling at 1.5 A, which is lower than that of the OER (2.85 Wh). Additionally, the values at the same current are 1.89 and 1.62 kWh kg^−1^_Ph-NHOH_ for the OER and GOR, respectively. This represents an electricity savings of approximately 14.3% compared with the case without glycerol, underscoring the superiority of coupling Ph-NHOH electrosynthesis with the GOR. The operating stability of Ph-NHOH electrosynthesis was evaluated in the assembled MEA at a current of 1.5 A over the CV-Ag catalyst. This MEA electrolyzer performs stable electrolysis for more than 5 h, achieving a total yield of 5.85 g of high-purity Ph-NHOH, as confirmed by ^1^H NMR (Supplementary Figs. 40, 41, Fig. 5a). Continuous Ph-NHOH production can be maintained by simply refreshing the electrolyte. The catalytic activity of the CV-Ag-based system does not degrade for more than 20 h for both cathodic Ph-NHOH electrosynthesis and anodic HCOOH production (Fig. 5e, Supplementary Fig. 42). Postreaction characterization confirms that the CV-Ag catalyst maintains its original morphology and valence state, with no observable detachment or oxidation (Supplementary Fig. 43, Supplementary Note 10 and Table 6). Finally, a preliminary technoeconomic analysis (TEA) on a plant gate-levelized cost per tonne of Ph-NHOH was conducted, comprehensively accounting for major cost contributors, including electricity and anodic reactant consumption^49^. The profit of Ph-NHOH is 12.87 $ Kg^−1^ at 1.5 A, demonstrating the industrial application potential of this electrosynthesis technique (Supplementary Fig. 44, Supplementary Notes 1, 11). Overall, this CV-Ag-based integrated system facilitated the sustainable synthesis of Ph-NHOH with high current and high selectivity, opening the door to potential future industrial production.

## Discussion

In summary, we theoretically predict and experimentally report that metallic Ag is highly active for the selective electrocatalytic hydrogenation of nitrobenzene (Ph-NO_2_) to *N*-phenylhydroxylamine (Ph-NHOH). The high selectivity and Faraday efficiency (FE) of Ph-NHOH originate from the weak adsorption of Ph-NHOH on the surface and subsequent easy desorption, thus suppressing its overhydrogenation to Ph-NH_2_. Afterward, a simple electrodeposition strategy is used to synthesize a small-sized Ag-based catalyst with a large electrochemical surface to enable amper-level gram-scale electrosynthesis of Ph-NHOH. Scalable Ph-NHOH synthesis (5.85 g per batch) maintains an FE of 99.6% under continuous electrolysis for more than 20 h at 1.5 A. This work highlights the critical role of adsorption in the electrochemical synthesis of Ph-NHOH and provides a green method for the sustainable electrosynthesis of hydroxylamine derivatives.

## Methods

### Materials preparation

A CV-Ag electrode was obtained via cyclic voltammetry (CV). For CV-Ag preparation, Ag foil (0.1 mm thickness) was first cut into 1.0 cm × 2.0 cm pieces and then washed in 3.0 M HCl for 15 min to remove surface oxides, followed by ultrasonication in ethanol and acetone for 15 min, respectively. Afterward, the Ag foil was immersed in an aqueous solution consisting of 5.0 mM AgNO_3_, 0.1 M KNO_3_, and 0.1 M trisodium citrate dihydrate (Na_3_C_6_H_5_O_7_·2H_2_O). The electrodeposition process was carried out at 25 °C in a three-electrode cell. The Ag foil served as the working electrode, and the KCl-saturated Ag/AgCl and the carbon rod served as the reference electrode and counter electrode, respectively. The growth of the CV-Ag was performed by applying a facile CV method with a potential range of 0.4 V to 0.2 V vs. Ag/AgCl at a scan rate of 50 mV s^−1^ for 800 cycles.

### Characterization

The morphology of the catalysts was observed via field emission scanning electron microscopy (FEI Apreo S LoVac) and transmission electron microscopy (Thermo Scientific Talos F200X). X-ray photoelectron spectroscopy measurements were performed on a Thermo Fisher Scientific K-Alpha spectrometer. All the peaks were calibrated by the binding energy of 284.8 eV of the C 1 s spectrum. X-ray diffraction tests were performed on a Rigaku SmartLab9 kW diffraction system. In situ ATR-FTIR was performed on a Nicolet 6700 FTIR spectrometer with silicon as the prismatic window. X-ray absorption spectroscopy was performed at BL08B2* of SPring-8 (8 GeV, 100 mA), Japan, in which the X-ray beam was monochromatized with a water-cooled Si (111) double-crystal monochromator and focused with two Rh-coated focusing mirrors with a beam size of 2.0 mm in the horizontal direction and 0.5 mm in the vertical direction around the sample position to obtain X-ray absorption fine structure (XAFS) spectra both in the near and extended edge. Ag foil samples were used as references. The X-ray absorption spectra were analysed with the ATHENA software package^50^. ^1^H nuclear magnetic resonance (NMR) data were recorded on a Bruker AVANCE III 600 MHz NMR instrument. HPLC was performed on a SHIMADZU Shim-pack GIST C18 chromatographic column (4.6 × 250 nm, 5 μm) and a UV detector (Essentia SPD-16). The corresponding mobile phase, flow rate, column temperature, and detection wavelength were methanol:water (3:2), 1.0 mL min^−1^, 35 °C, and 280 nm, respectively. Notably, sample preparation and chromatographic detection are carried out as soon as possible to prevent oxidation of the products.

### Electrochemical measurements

All the electrochemical measurements were carried out on a Koster electrochemical workstation (CS 6.2) using an H-type electrolytic cell separated by a Nafion 117 membrane at room temperature (25 °C). The Nafion 117 membrane was protonated by boiling in ultrapure water, H_2_O_2_ (5.0%) aqueous solution, and 0.5 M H_2_SO_4_ at 80 °C for another 2 h before use. The anodic electrolyte was a 0.5 M phosphate solution (pH = 5.8), and the cathodic electrolyte was an Ar-saturated 0.5 M phosphate solution (pH = 5.8, 8 mL) with 0.2 mmol nitrobenzene (Ph-NO_2_ with 500 µl dioxane). The working electrode was a 1.0 × 1.0 cm metal plate (Geometric area: 2 cm^2^) immersed in the electrolyte, with both sides exposed to the solution. Ag/AgCl (KCl-saturated) and a carbon rod were used as the reference electrode and the counter electrode, respectively. All the potentials were converted relative to the reversible hydrogen electrode (vs. RHE) via the following formula. All electrochemical data were recorded without iR correction.

$${E}_{{{{\rm{RHE}}}}}=\,{E}_{{{{\rm{A}}}}{{{\rm{g}}}}/{{{\rm{A}}}}{{{\rm{g}}}}{{{\rm{C}}}}{{{\rm{l}}}}}+0.059\times \,{pH}+0.199$$

Before the electroreduction test, linear sweep voltammetry (LSV) was performed until the polarization curves reached a steady state at a scan rate of 10 mV s^−1^ from 0.2 to –0.5 V vs. RHE. All the potentiostatic tests were subsequently carried out at different potentials at 77 C with a stirring rate of 800 rpm.

### Electrochemical in situ ATR-FTIR spectra measurements

In situ attenuated total reflection Fourier transform infrared spectroscopy (ATR-FTIR) was performed on a Nicolet 6700 FTIR spectrometer. A thin gold film was chemically deposited on the surface of a silicon crystal. The ink from the sample was dropped on the aforementioned gold film supported by silicon, and the whole sample served as the working electrode. A carbon rod and Ag/AgCl were used as the counter electrode and reference electrode, respectively. LSV curves were obtained from 0.3 to –0.6 V vs. RHE with a scan rate of 2.0 mV s^–1^.

### Product identification and quantification

The liquid products were detected via ^1^H NMR in water suppression mode and quantified via HPLC calibration curves. After the electrochemical test, 500 μL of the electrolyte, 100 μL of dimethylmalonic acid, and 100 μL of DMSO-d_6_ were mixed in the NMR tube for tests (the NMR tubes were purged with argon before sample injection). The amount of the analyte was calculated on the basis of the ratio of the area of the analyte peak in the HPLC chromatogram. The error bars correspond to the standard deviation of three independent measurements.

Calculation of the FE, conversion, and selectivity:

$${FE}=\frac{{N}_{{{{\rm{Ph}}}}-{{{\rm{NHOH}}}}}\times F\times [{{{\rm{Ph}}}}-{{{\rm{NHOH}}}}]\times V}{{{{{\rm{M}}}}}_{{{{\rm{Ph}}}}-{{{\rm{NHOH}}}}}\times {{{\rm{Q}}}}}\times 100\%$$

$${Selectivity}({Sel}.)=\frac{n({{{\rm{as}}}}-{{{\rm{formed\; Ph}}}}-{{{\rm{NHOH}}}})}{n({{{\rm{consumed\; Ph}}}}-{{{\rm{N}}}}{{{{\rm{O}}}}}_{2})}\times 100\%$$

$${Coversion\; yield}\left({Conn}.\right)=\frac{n\left({{{\rm{consumed\; Ph}}}}-{{{\rm{N}}}}{{{{\rm{O}}}}}_{2}\right)}{n\left({{{\rm{added\; Ph}}}}-{{{\rm{N}}}}{{{{\rm{O}}}}}_{2}\right)}\times 100\%$$

where *n* is the electron-transfer number (4), *F* is the Faradic constant (96,485 C mol^–1^), [Ph-NHOH] is the measured concentration, *V* is the volume of the electrolyte in the cathode chamber (8.5 mL), *M*_Ph-NHOH_ is the molar mass of Ph-NHOH, *Q* is the total quantity of applied electricity, and *t* is the electrolysis time.

### Assembly of a membrane electrode electrolyzer for large-scale synthesis experiments

Amplified electrolysis experiments were performed on a 25 cm^2^ two-electrode flow cell separated by a Nafion 117 membrane at room temperature with a CS6.2 electrochemical workstation. The flow electrolyzer (Wuhan Zhisheng New Energy Co., Ltd.) was assembled with two stainless steel plates (cover plates), two copper plates (current collectors), and two platinum-coated titanium plates with a serpentine flow field (electrolysis chamber). Carbon cloth (5.0 cm × 5.0 cm) was used as the cathode (with electroplated CV-Ag catalyst) and anode (Ni foam). 0.5 M phosphate solution (pH = 5.8) and 2.0 M KOH served as the catholyte and anolyte, respectively. Ph-NO_2_ (50 mM) dissolved in dioxane was added to the catholyte, and 0.2 M glycerol was added to the anolyte. After that, galvanostatic electrolysis was conducted at a flow rate of 30 mL min^−1^. In a single electrolysis process, the volume of the cathode electrolyte was 0.5 L. Long-term electrolysis was conducted at a constant current for 20 h in batch operation mode, during which the catholyte was completely replaced with freshly prepared electrolytes every 5 h.

### Calculation of electrical energy consumption

The values of electrical energy consumption (${W}_{{{{\rm{E}}}}}$) with and without AA are calculated via the following equation:

$${W}_{{{{\rm{E}}}}}=I\times U\times t$$

where I denotes the current (1.5 A), U is the reaction potential, and t is the reaction time (1 h).

The calculated values of the energy required to produce per of Ph-NHOH (${W}_{{{{\rm{P}}}}{{{\rm{h}}}}-{{{\rm{NHOH}}}}}$), with and without AA, are determined via the following equation:

$${W}_{{{{\rm{P}}}}{{{\rm{h}}}}-{{{\rm{NHOH}}}}}=(n\times F\times U)/(3600\times M\times {FE})$$

where n denotes the number of electrons transferred, U denotes the reaction potential at 1.5 A, F is the Faraday constant (96,485 C mol^–1^), ${FE}$ is the Faradaic efficiency at 1.5 A, and M is the molar mass at normal temperature and pressure.

### Product purification

After the electrolysis of Ph-NO_2_, the mixture was extracted with ethyl acetate (EA). The organic phase was dried over anhydrous sodium sulfate, filtered, and concentrated by vacuum distillation to remove ethyl acetate, yielding the crude product. The crude product was further recrystallized using a mixed solvent of petroleum ether and dichloromethane, affording white Ph-NHOH crystals as the final product. The product was detected via ^1^H NMR.

### Computational models and methods

All density functional theory (DFT) calculations were performed via the Vienna Ab initio Simulation Package (VASP) with the generalized gradient approximation functional^51–53^. Five common metals (Pd, Pt, Ru, Cu, and Ag) were investigated to evaluate their performance in the electroreduction of nitrobenzene (Ph-NO_2_) to phenylhydroxylamine (Ph-NHOH). The adsorption configurations of the reaction intermediates on different metals were provided in Supplementary Data 1. The catalyst interfaces were modeled via face-centered cubic (111) surfaces. A vacuum layer of approximately 15 Å was applied along the z-direction in all the structures to prevent interactions between the periodic images.

### Reaction free energy (ΔG) calculations

The reaction free energies of the Ph-NO_2_ reduction elementary steps are the energy differences between the DFT-calculated energies of the product and reactant states. The computational hydrogen electrode (CHE) model proposed by Nørskov was adopted, as shown below^51^:

$${{{\rm{H}}}}+{{{{\rm{e}}}}}^{-}\to \frac{1}{2}{{{{\rm{H}}}}}_{2}$$

The chemical potential of the solvated proton and electron pair (${{{\rm{H}}}}+\,{{{{\rm{e}}}}}^{-}$) is calculated as 1/2 G(H_2_) + eUSHE (SHE represents the standard hydrogen electrode), which is assumed to achieve equilibrium under standard conditions (0 V vs. RHE, pH = 0, *P* = 1 bar, and T = 298.15 K).

### Adsorption energy (G_ad_) calculations

Additionally, reference systems, including graphene (C), nitrosobenzene (Ph-NO), water (H₂O), and hydrogen (H_2_), were modeled to establish energy benchmarks for C, H, O, and N. On the basis of these references, the adsorption energy (E_ad_) of all adsorbates (*C_a_N_b_H_c_O_d_) on the catalyst surfaces was calculated via the following equation^54,55^:

$${E}_{ad}\left({ * C}_{{{{\rm{a}}}}}{N}_{{{{\rm{b}}}}}{H}_{{{{\rm{c}}}}}{O}_{{{{\rm{d}}}}}\right)={E}_{{{{\rm{t}}}}}-({E}_{ * }+{{{\rm{a}}}}{E}_{{{{\rm{C}}}}}+{{{\rm{b}}}}{E}_{{{{\rm{N}}}}}+{{{\rm{c}}}}{E}_{{{{\rm{H}}}}}+{{{\rm{d}}}}{E}_{{{{\rm{O}}}}})$$

where ${E}_{{{{\rm{t}}}}}$ is the total DFT-calculated energy of each intermediate on the catalyst and E* is the DFT-calculated energy of the bare surface.

Furthermore, the adsorption free energy correction was based on equation^56^, which includes the zero-point energy (${ZPE}$), entropy (S), and solvation energy (${E}_{{{{\rm{sol}}}}}$)^57^, as shown in Supplementary Tables 2, 3.

$${G}_{ad}\left({ * C}_{{{{\rm{a}}}}}{N}_{{{{\rm{b}}}}}{H}_{{{{\rm{c}}}}}{O}_{{{{\rm{d}}}}}\right)={E}_{{{{\rm{ad}}}}}\left( * {C}_{{{{\rm{a}}}}}{N}_{{{{\rm{b}}}}}{H}_{{{{\rm{c}}}}}{O}_{{{{\rm{d}}}}}\right)+{ZPE}-{TS}+{E}_{{{{\rm{sol}}}}}$$

The considered elementary steps:

$${{{{\rm{C}}}}}_{6}{{{{\rm{H}}}}}_{5}{{{{\rm{NO}}}}}_{2}+* +{{{{\rm{H}}}}}^{+}+{{{{\rm{e}}}}}^{-}\to \, * {{{{\rm{C}}}}}_{6}{{{{\rm{H}}}}}_{5}{{{{\rm{NO}}}}}_{2}{{{\rm{H}}}}$$

$$ * {{{{\rm{C}}}}}_{6}{{{{\rm{H}}}}}_{5}{{{{\rm{NO}}}}}_{2}{{{\rm{H}}}} \,+\,{{{{\rm{H}}}}}^{+}+{{{{\rm{e}}}}}^{-}\to \,{ * {{{\rm{C}}}}}_{6}{{{{\rm{H}}}}}_{5}{{{\rm{NO}}}}+{{{{\rm{H}}}}}_{2}{{{\rm{O}}}}$$

$$ * {{{{\rm{C}}}}}_{6}{{{{\rm{H}}}}}_{5}{{{\rm{NO}}}} \,+\,{{{{\rm{H}}}}}^{+}+{{{{\rm{e}}}}}^{-}\to \,{ * {{{\rm{C}}}}}_{6}{{{{\rm{H}}}}}_{5}{{{\rm{NOH}}}}$$

$${ * {{{\rm{C}}}}}_{6}{{{{\rm{H}}}}}_{5}{{{\rm{NO}}}} \,+\,{{{{\rm{H}}}}}^{+}+{{{{\rm{e}}}}}^{-}\to \,{ * {{{\rm{C}}}}}_{6}{{{{\rm{H}}}}}_{5}{{{\rm{NHO}}}}$$

$$ * {{{{\rm{C}}}}}_{6}{{{{\rm{H}}}}}_{5}{{{\rm{NOH}}}} \,+\,{{{{\rm{H}}}}}^{+}+{{{{\rm{e}}}}}^{-}\to \,{ * {{{\rm{C}}}}}_{6}{{{{\rm{H}}}}}_{5}{{{\rm{NHOH}}}}$$

$$ * {{{{\rm{C}}}}}_{6}{{{{\rm{H}}}}}_{5}{{{\rm{NOH}}}} \,+\,{{{{\rm{H}}}}}^{+}+{{{{\rm{e}}}}}^{-}\to {{{{\rm{C}}}}}_{6}{{{{\rm{H}}}}}_{5}{{{\rm{NHOH}}}}+* $$

$$ * {{{{\rm{C}}}}}_{6}{{{{\rm{H}}}}}_{5}{{{\rm{NHO}}}} \,+\,{{{{\rm{H}}}}}^{+}\,+\,{{{{\rm{e}}}}}^{-}\,\to \,{ * {{{\rm{C}}}}}_{6}{{{{\rm{H}}}}}_{5}{{{\rm{NHOH}}}}$$

$$ * {{{{\rm{C}}}}}_{6}{{{{\rm{H}}}}}_{5}{{{\rm{NHO}}}}+{{{{\rm{H}}}}}^{+}+{{{{\rm{e}}}}}^{-}\to {{{{\rm{C}}}}}_{6}{{{{\rm{H}}}}}_{5}{{{\rm{NHOH}}}}+* $$

$$ * {{{{\rm{C}}}}}_{6}{{{{\rm{H}}}}}_{5}{{{\rm{NHOH}}}}+{{{{\rm{H}}}}}^{+}+{{{{\rm{e}}}}}^{-}\to {{{{\rm{ * C}}}}}_{6}{{{{\rm{H}}}}}_{5}{{{\rm{NH}}}}+{\rm{H}}_{2}{\rm{O}}$$

$$ * {{{{\rm{C}}}}}_{6}{{{{\rm{H}}}}}_{5}{{{\rm{NH}}}}+{{{{\rm{H}}}}}^{+}+{{{{\rm{e}}}}}^{-}\to {{{{\rm{C}}}}}_{6}{{{{\rm{H}}}}}_{5}{{{{\rm{NH}}}}}_{2}+* $$

## Supplementary information


Supplementary Information
Description of Additional Supplementary Files
Supplementary Data 1
Transparent Peer Review file


## Source data


Source Data


## Data Availability

All the data needed to evaluate the conclusions in the paper are presented in the paper and/or its Supplementary Materials (characterization, electrochemical in situ spectra measurements, supplementary technoeconomic analysis, and calculation details). Additional data related to this paper may be requested from the authors. Source data are provided with this paper.
