# Supplementary material for: Metal-mediated weak adsorption enables the selective electrosynthesis of N-phenylhydroxylamine from nitrobenzene hydrogenation in water
Source: Nat Commun. 2026 Jul 30;17:9221. doi: 10.1038/s41467-026-76066-6 (PMC13522461; doi:10.1038/s41467-026-76066-6)
Supplement: Supplementary file 1 [file 41467_2026_76066_MOESM1_ESM.pdf]

Table of contents

Supplementary Figures 1–44

Supplementary Notes 1–12

Supplementary Tables 1–6

Supplementary References 1–18

Supplementary technoeconomic analysis (TEA)

Taking the production of Ph-NHOH with a daily capacity of 5000 kg at a current density of 0.12 A cm^{-2} and a 99% FE of Ph-NHOH as an example.

The specific assumptions made for the TEA are listed as follows:

The electricity price was considered to be 0.04 \$ kWh.

The operating voltage was set as 1.63 V.

The electrolyzer cost per area was calculated as follows:[1]

$$Electrolyzer\ Cost\ per\ area\ = 275\ \$\ kW^{-1} \times \frac{0.12\ A}{cm^2} \times 1.63\ V \times \frac{10^4 cm^2}{m^2} \times \frac{kW}{1000} = 537.9\ \$\ m^{-2}$$

The anticipated service life of the electrolyzer was 20 years, and the working time per year was 350 days[2]. It is assumed that the electrolyzer stacks do not have to be replaced during the lifetime of the plant.

4, The separation cost is composed of two parts: the separation cost of Ph-NHOH and that of HCOOH[3]. We set the Ph-NHOH separation yield at 80%.

Thus, the parameters needed in the cost calculation are determined as follows:

$$The\ Current = \frac{5000\ Kg}{day} \times \frac{day}{86400\ s} \times \frac{1000g}{Kg} \times \frac{mol}{109\ g} \times 4e^- \times \frac{96485\ C}{mol} \times \frac{1}{0.99} \times \frac{1}{0.8} = 258716.64\ A$$

$$Ph - NO_2\ Weight = 258716.64\ A\ \times \frac{1}{4e^-} \times \frac{1\ mol}{96485\ C} \times \frac{0.123\ Kg}{mol} \times \frac{86400\ s}{day} = 7123.99\ Kg\ day^{-1}$$

$$Glycerol\ Weight = 258716.64\ A\ \times \frac{1}{8e^-} \times \frac{1\ mol}{96485\ C} \times \frac{0.092\ Kg}{mol} \times \frac{86400\ s}{day} \times 0.4 = 1065.71\ Kg\ day^{-1}$$

$$HCOOH\ Weight = 258716.64\ A\ \times \frac{1}{8e^-} \times 3 \times \frac{1\ mol}{96485\ C} \times \frac{0.046\ Kg}{mol} \times \frac{86400\ s}{day} \times 0.4 = 1598.55\ Kg\ day^{-1}$$

$$Electrolyzer\ Area = 258716.64\ \ A \times \frac{1}{0.12\ A\ cm^{-2}} \times \frac{m^2}{10^4\ cm^2} = 215.60\ m^2$$

$$Power\ = 1.63\ V\ \times 258716.64\ \ A \times \frac{MW}{10^6 W} = 0.422\ MW$$

The Ph-NO_2 flow rate is as follows:

$$Ph - NO_2\ folw\ rate = 7123.99\ \frac{Kg}{day} \times \frac{m^3}{1205\ Kg} \times \frac{day}{24\ h} = 4.11\ \frac{L}{min}$$

The glycerol flow rate is as follows:

$$Glycerol\ folw\ rate = 1065.71\ \frac{Kg}{day} \times \frac{m^3}{1250\ Kg} \times \frac{day}{24\ h} = 0.036\ \frac{L}{min}$$

It is assumed that the Ph-NHOH and HCOOH electrolytes are recycled until they reach a steady–state volume concentration of 10%. The continuous exit electrolyte flow rate is as follows:

$$Ph - NHOH\ Electrolyte\ folw\ = 4.11\ \frac{L}{min} \times \frac{1}{0.1} = 41.1\frac{L}{min}$$

$$HCOOH\ Electrolyte\ folw\ = 0.036\ \frac{L}{min} \times \frac{1}{0.1} = 0.36\frac{L}{min}$$

Cost:

Electrolyzer:

$$Electrolyzer\ Cost = 537.9\ \$\ m^{-2} \times 215.60\ m^2 \times \frac{1}{20\ year} \times \frac{1}{350\ day}$$

$$= 16.57\ \$\ day^{-1}$$

Electricity:

$$Electricity\ Cost\ = 0.422\ MW \times \frac{1000\ kW}{MW} \times \frac{24\ h}{day} \times \frac{0.04\ \$}{kWh} = 438.72\ \$\ day^{-1}$$

Separation cost:

$$Ph - NHOH\ \text{Separation Cost} = 4162240\ \$ \times \left(\frac{41.1\ \frac{L}{min}}{1000\ \frac{L}{min}}\right)^{0.7} \times \frac{1}{20\ year} \times \frac{1}{350\ day}$$

$$= 63.78\ \$\ day^{-1}$$

$$\text{HCOOH Separation Cost} = 4162240\ \$ \times \left(\frac{0.36\ \frac{L}{min}}{1000\ \frac{L}{min}}\right)^{0.7} \times \frac{1}{20\ year} \times \frac{1}{350\ day}$$

$$= 2.31\ \$\ day^{-1}$$

Material Cost:

$$Ph - NO_2\ Cost = 7123.99\ Kg\ day^{-1} \times 5\ \$\ Kg^{-1} = 35619.95\ \$\ day^{-1}$$

$$Glycerol\ Cost = 1065.71\ \ Kg\ day^{-1} \times 0.97\ \$\ Kg^{-1} = 1033.74\ \$\ day^{-1}$$

$$Water\ Cost = 258716.64\ A \times 0.6 \times \frac{1}{4e^-} \times \frac{mol}{96485\ C} \times \frac{0.018\ Kg}{mol} \times \frac{0.2642\ gal}{Kg} \times \frac{86400\ s}{day} \times \frac{0.0054\ \$}{gal} = 0.89\ \$\ day^{-1}$$

$$Total\ Cost = Electrolyzer\ Cost + Electricity\ Cost + \text{Separation Cost} + Water\ Cost + Material\ Cost$$

$$Total\ Cost = \frac{16.57 + 438.72 + 63.78 + 2.31 + 35619.95 + 1033.74 + 0.89}{5000} = 7.44\ \$\ day^{-1}Kg_{Ph-NHOH}^{-1}$$

$$Profit(GOR) = 20 + \frac{1598.55\ \times\ 0.97}{5000} - 7.44 = 12.87\ \$\ day^{-1}Kg_{Ph-NHOH}^{-1}$$

Supplementary Note 1: After we set the prices of Ph-NHOH (20 \$ kg^{-1}) and Ph-NO_2 (5 \$ kg^{-1}), the fluctuation of the balance is treated as a function of two single parameters: the Ph-NHOH FE and the electricity price.

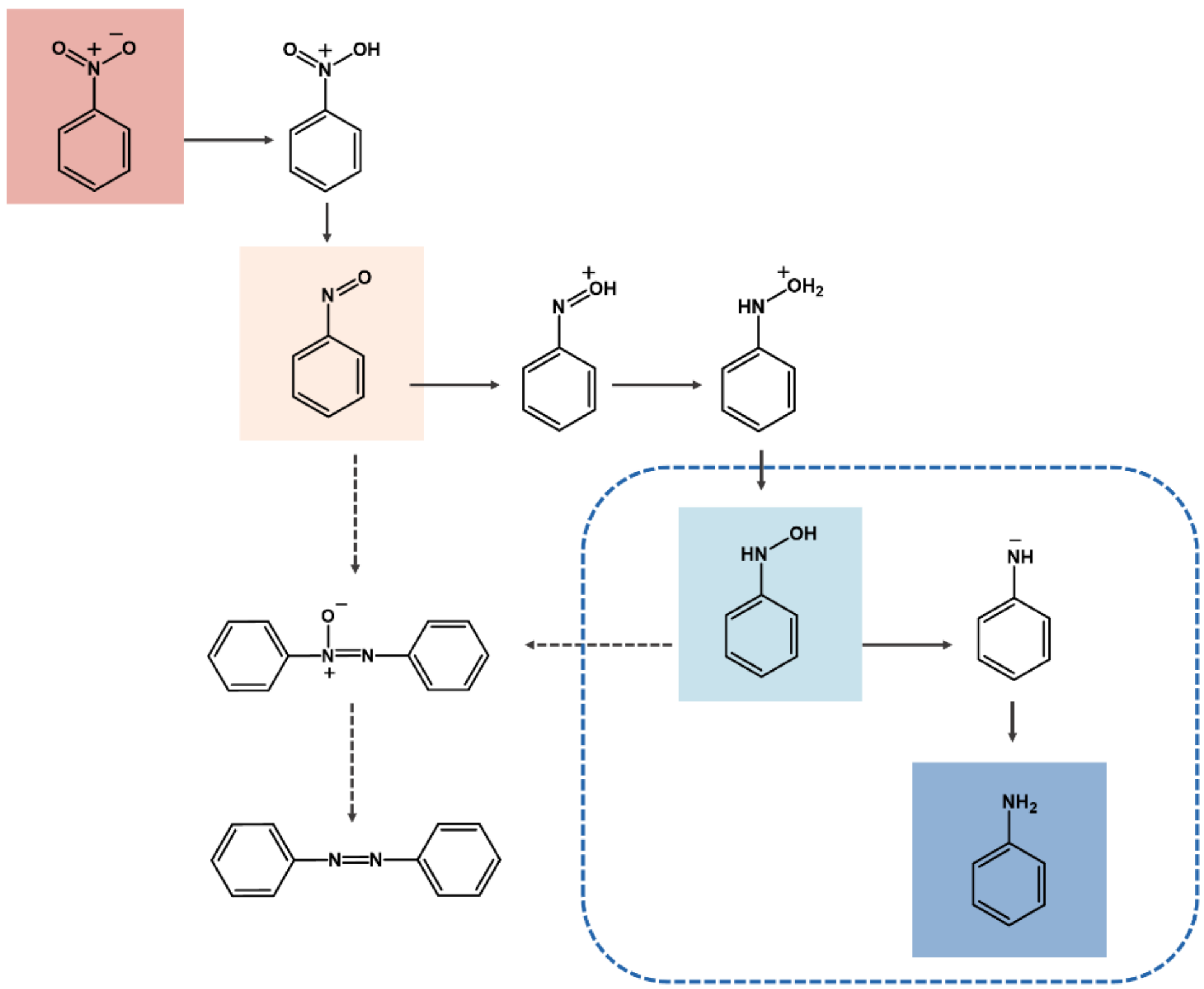

Supplementary Figure 1 Schematic illustration of the electrocatalytic hydrogenation of Ph-NO_2[4].

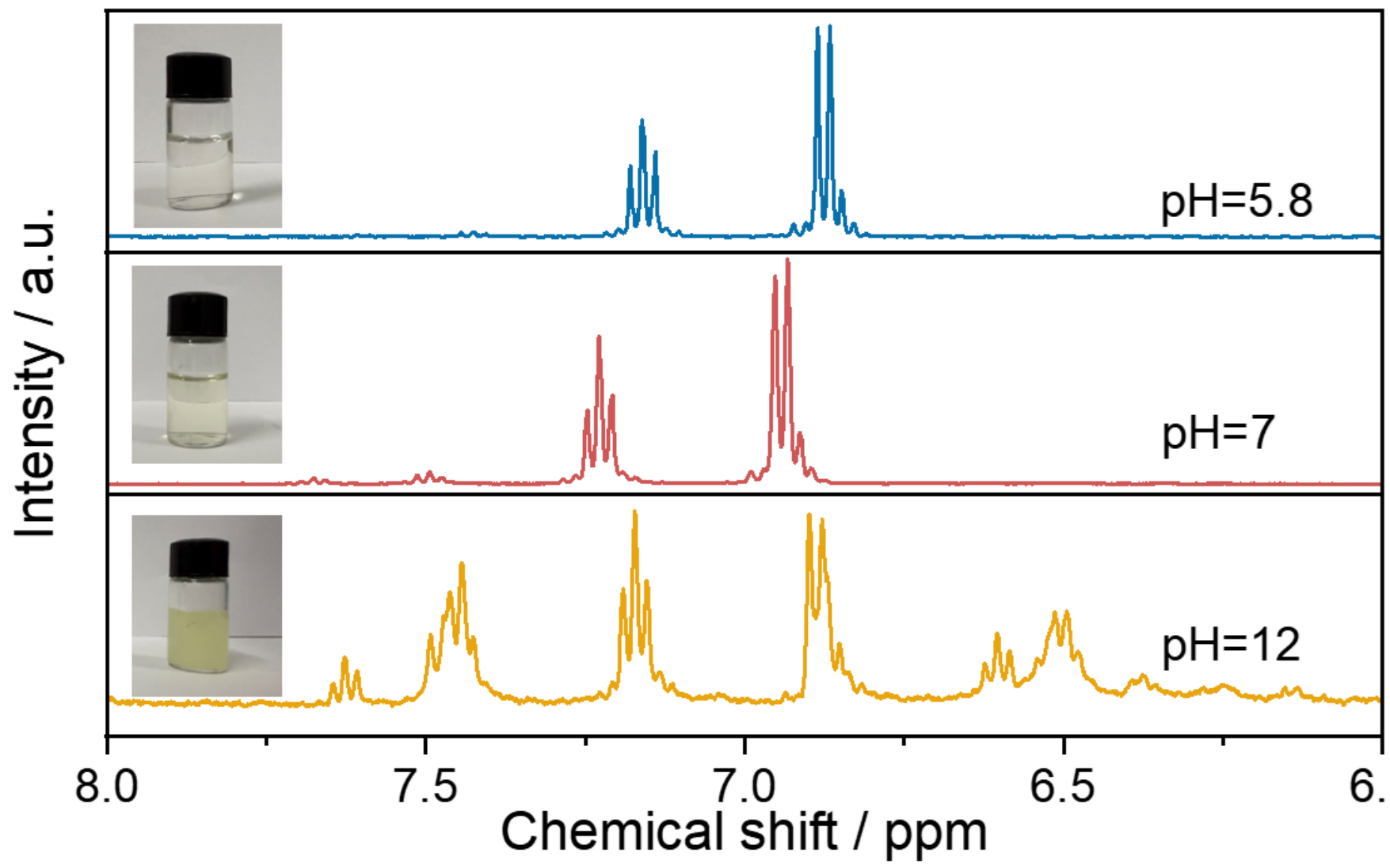

Supplementary Figure 2 ^{1}H NMR spectra of products after electroreduction in different electrolytes with different pH values for 10 min. Source data for Fig. S2 are provided as a Source Data file.

Supplementary Note 2 Ph-NHOH was rapidly converted to azoxybenzene after being placed in a high-pH solution for 10 min, indicating that a weakly acidic solution was optimal for Ph-NHOH synthesis.

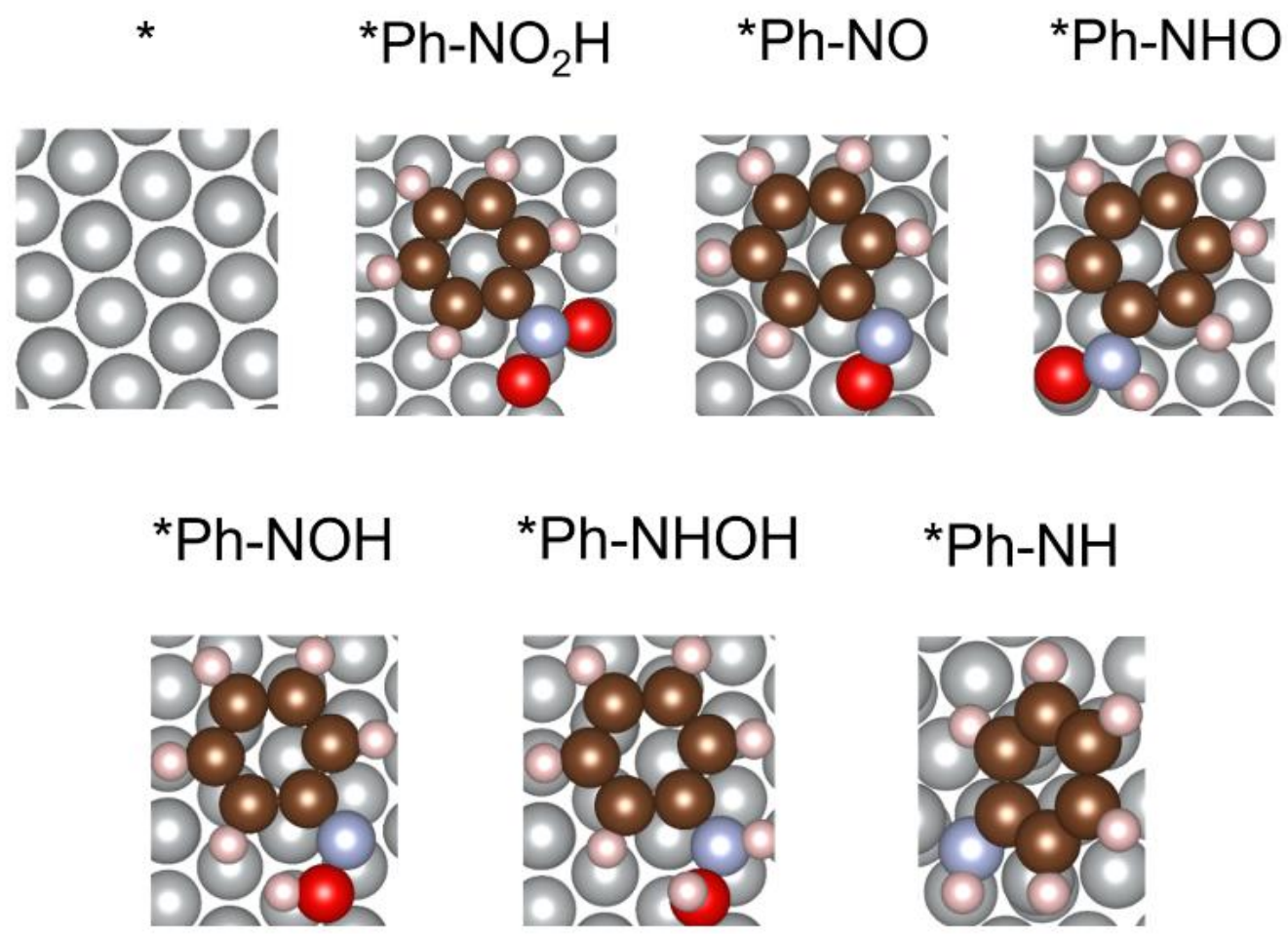

Supplementary Figure 3 Structural model of the Ph-NO_2 reduction reaction over Ag metal.

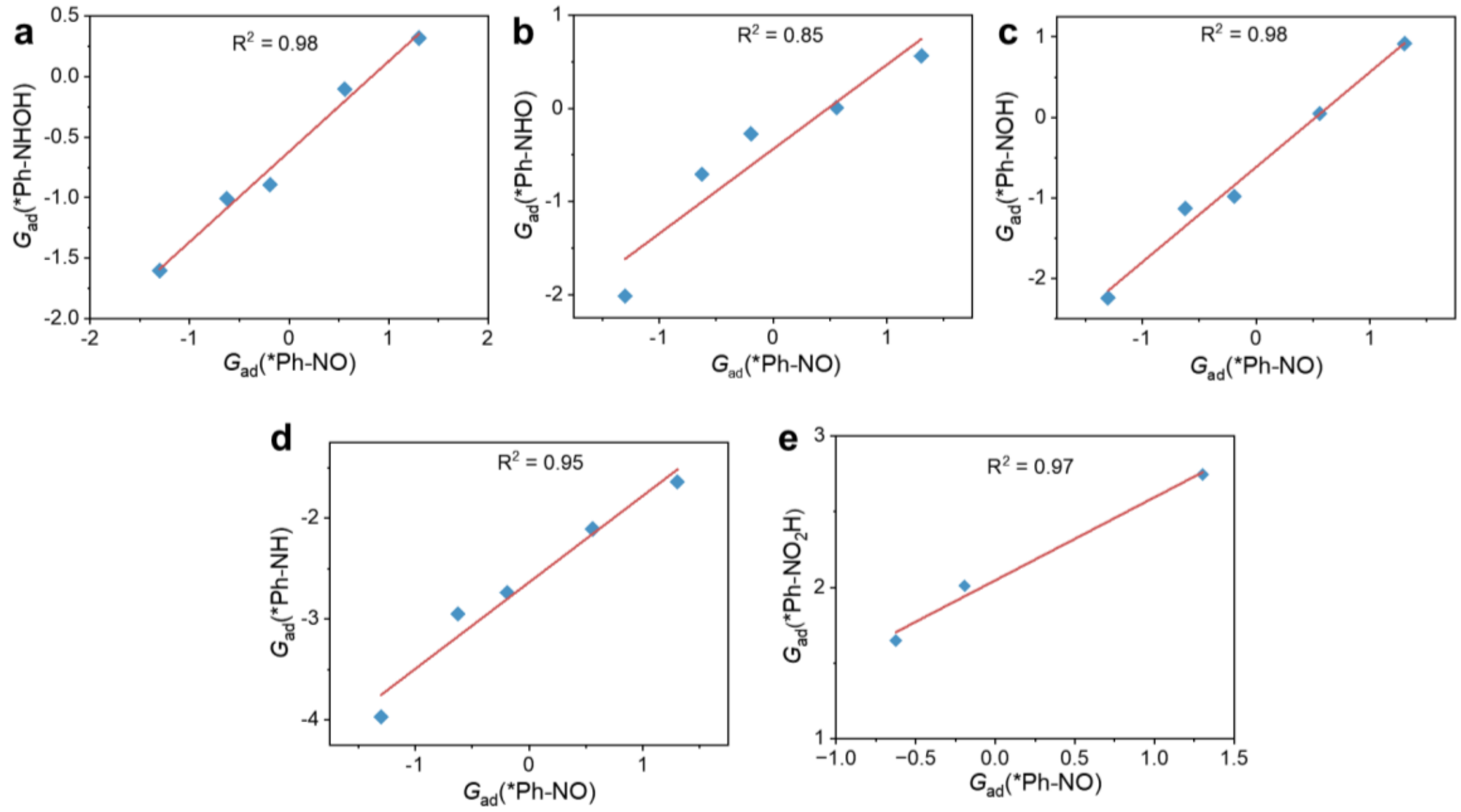

Supplementary Figure 4 Scaling relationships of the various adsorbed intermediates involved in the Ph-NO_2 reduction reaction over metallic Ag. a, G_{ad}(*Ph-NHOH) vs. G_{ad}(*Ph-NO). **b,** G_{ad}(*Ph-NHO) vs. G_{ad}(*Ph-NO). **c,** G_{ad}(*Ph-NOH) vs. G_{ad}(*Ph-NO). **d,** G_{ad}(*Ph-NH) vs. G_{ad}(*Ph-NO). **e,** G_{ad}(*NO_2H) vs. G_{ad}(*Ph-NO). Source data for Fig. S4 are provided as a Source Data file.

Supplementary Note 3 Almost all R^2 values are greater than 0.8, indicating that these scaling relationships are reliable. By utilizing these scaling relationships, a volcano plot for the conversion of Ph-NO_2 to Ph-NHOH and Ph-NH_2 was constructed, as shown in Fig. 2a.

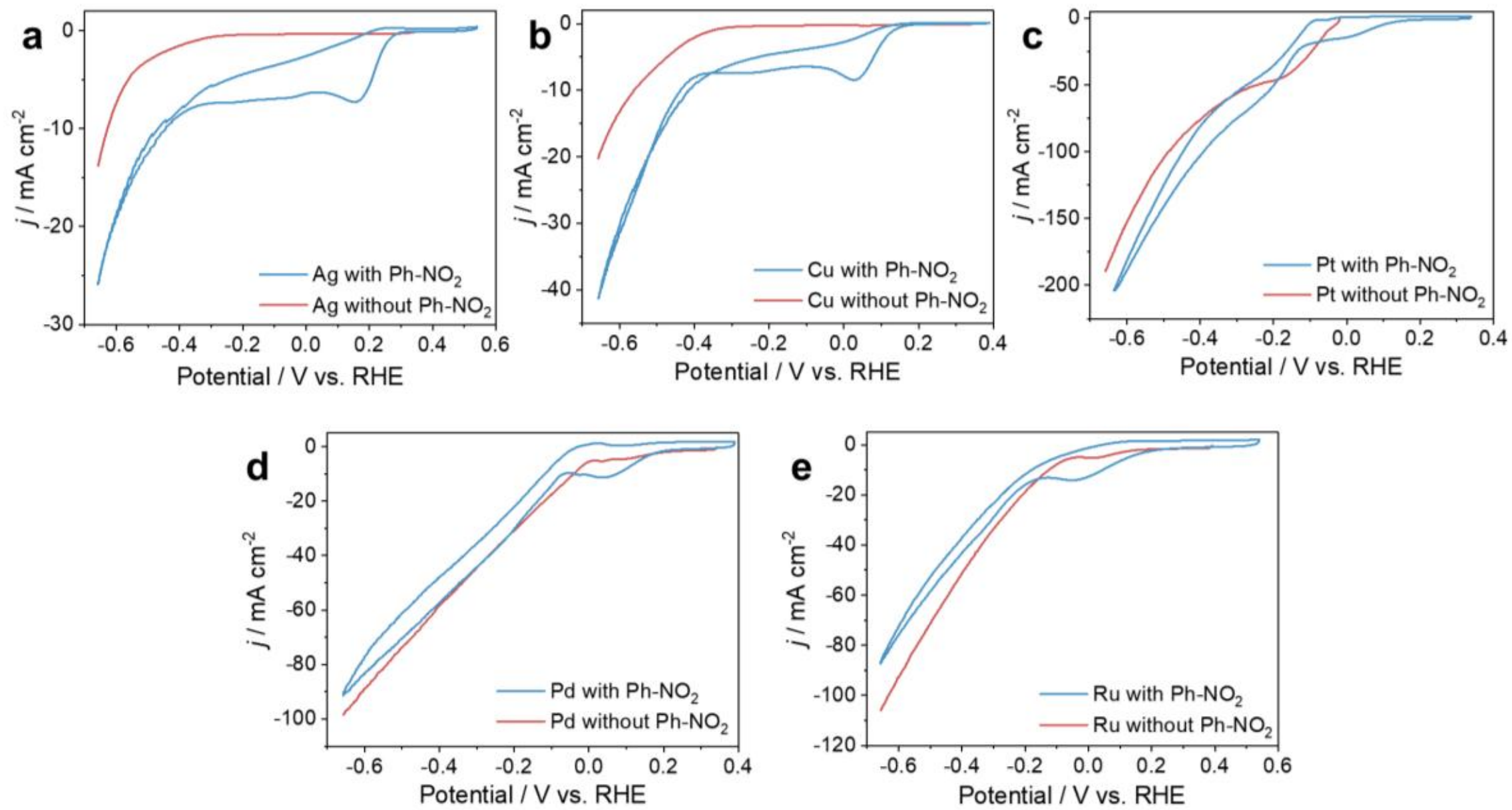

Supplementary Figure 5 CV curves of metallic samples. a–e, Ag, Cu, Pt, Pd, and Ru with/without Ph-NO_2 at 10 mV s^{-1}. Source data for Fig. S5 are provided as a Source Data file.

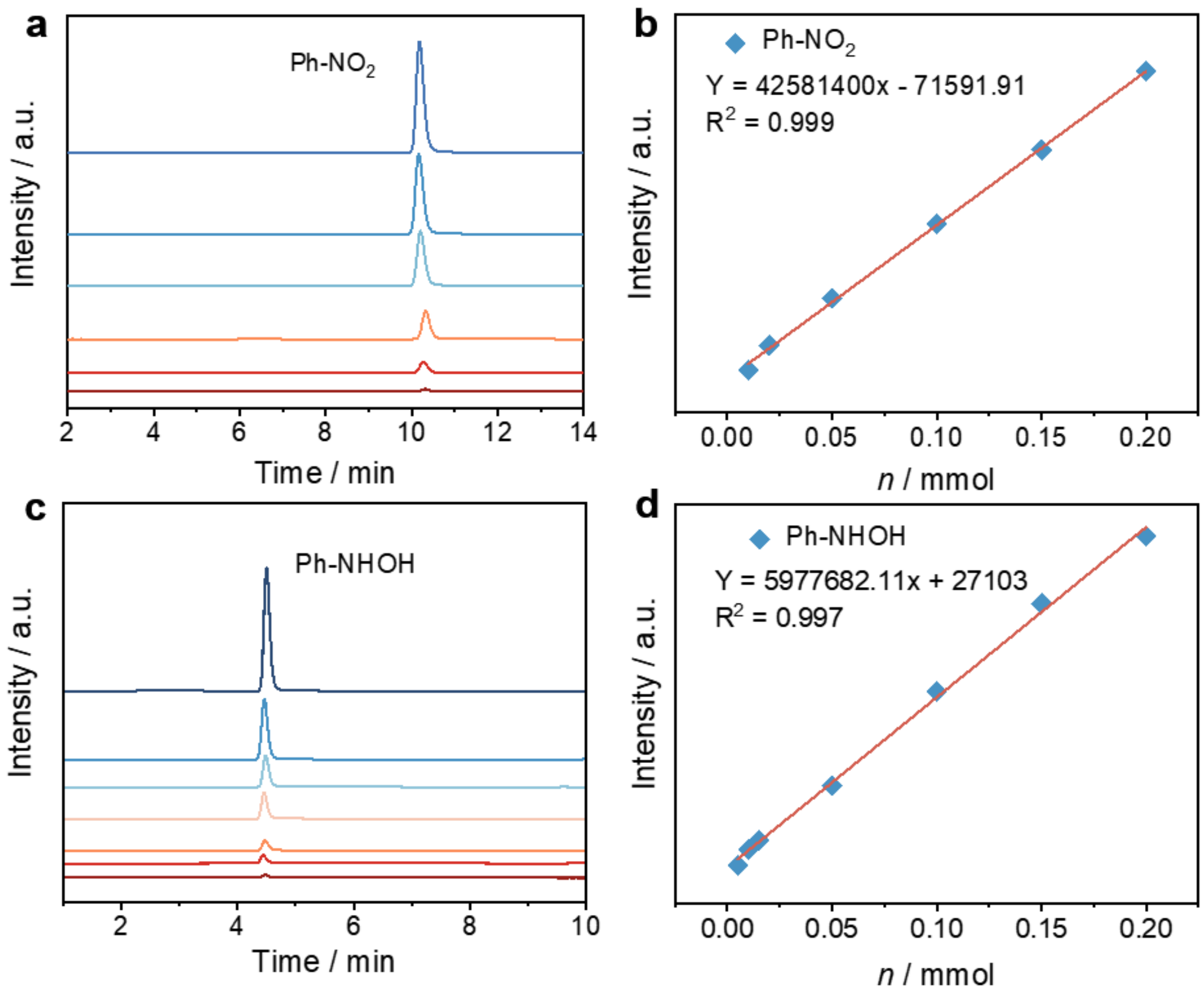

Supplementary Figure 6 Determination by HPLC. a-b, Calibration curve of Ph-NO_2. **c-d**, Calibration curve of Ph-NHOH. Source data for Fig. S6 are provided as a Source Data file.

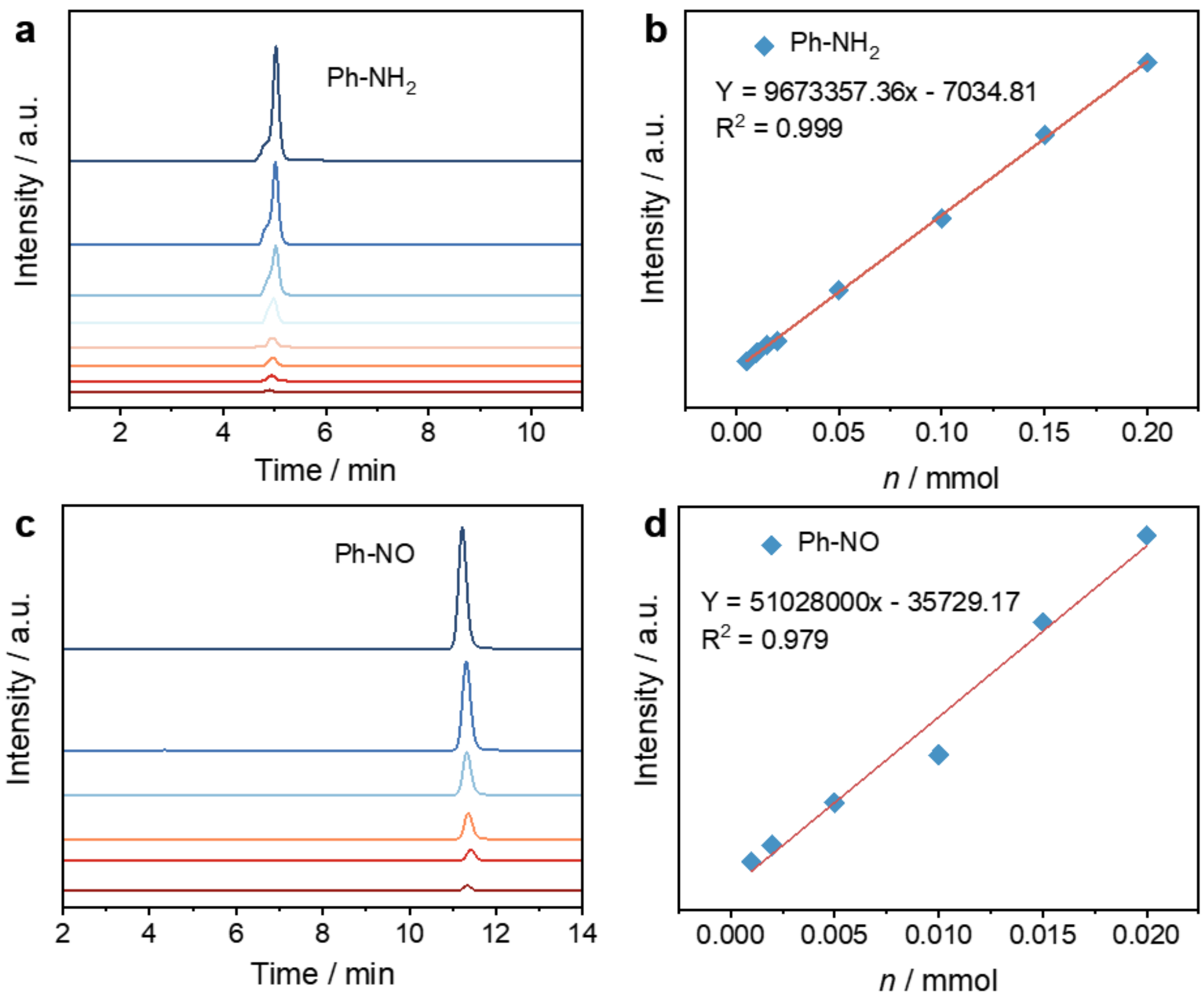

Supplementary Figure 7 Determination by HPLC. a-b, Calibration curve of Ph-NH_2. **c-d**, Calibration curve of Ph-NO. Source data for Fig. S7 are provided as a Source Data file. Source data for Fig. S7 are provided as a Source Data file.

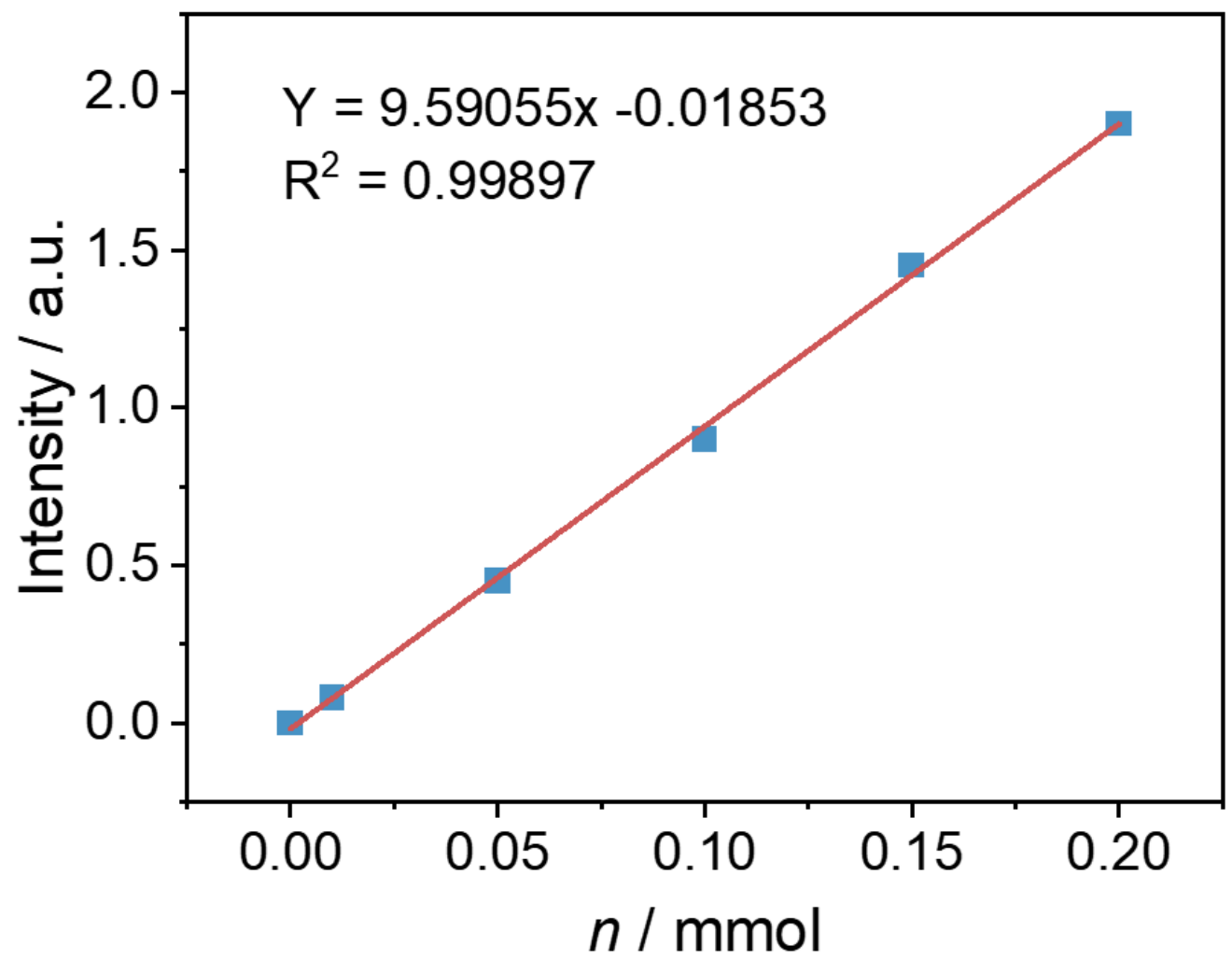

Supplementary Figure 8 Determination by ^{1}H NMR. The calibration curve of Ph-NHOH. Source data for Fig. S8 are provided as a Source Data file.

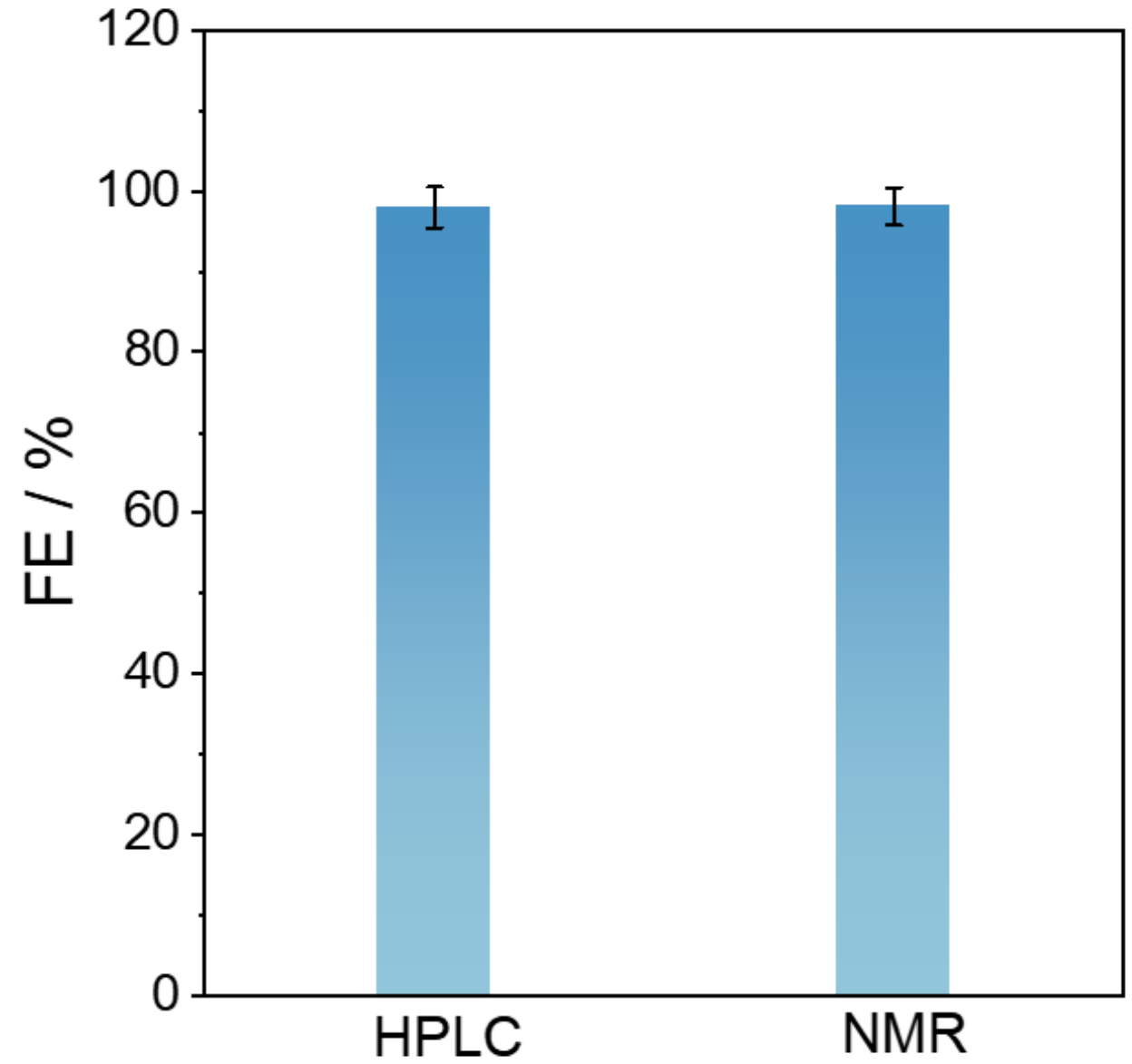

Supplementary Figure 9 Comparison of determination by HPLC and ^{1}H NMR.

Source data for Fig. S9 are provided as a Source Data file.

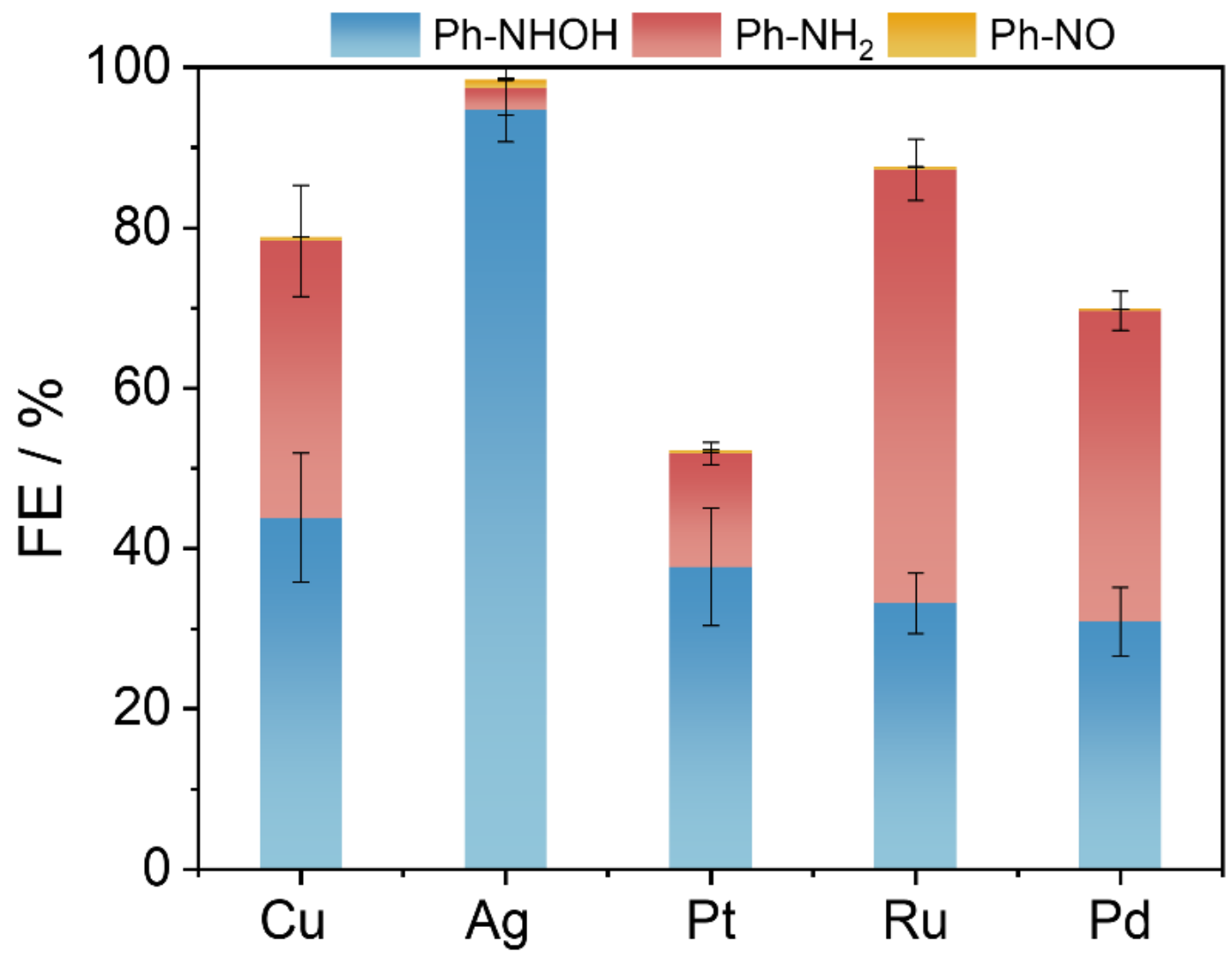

Supplementary Figure 10 Electroreduction of Ph-NO₂ over different bulk metals at −0.2 V vs. RHE. Source data for Fig. S10 are provided as a Source Data file.

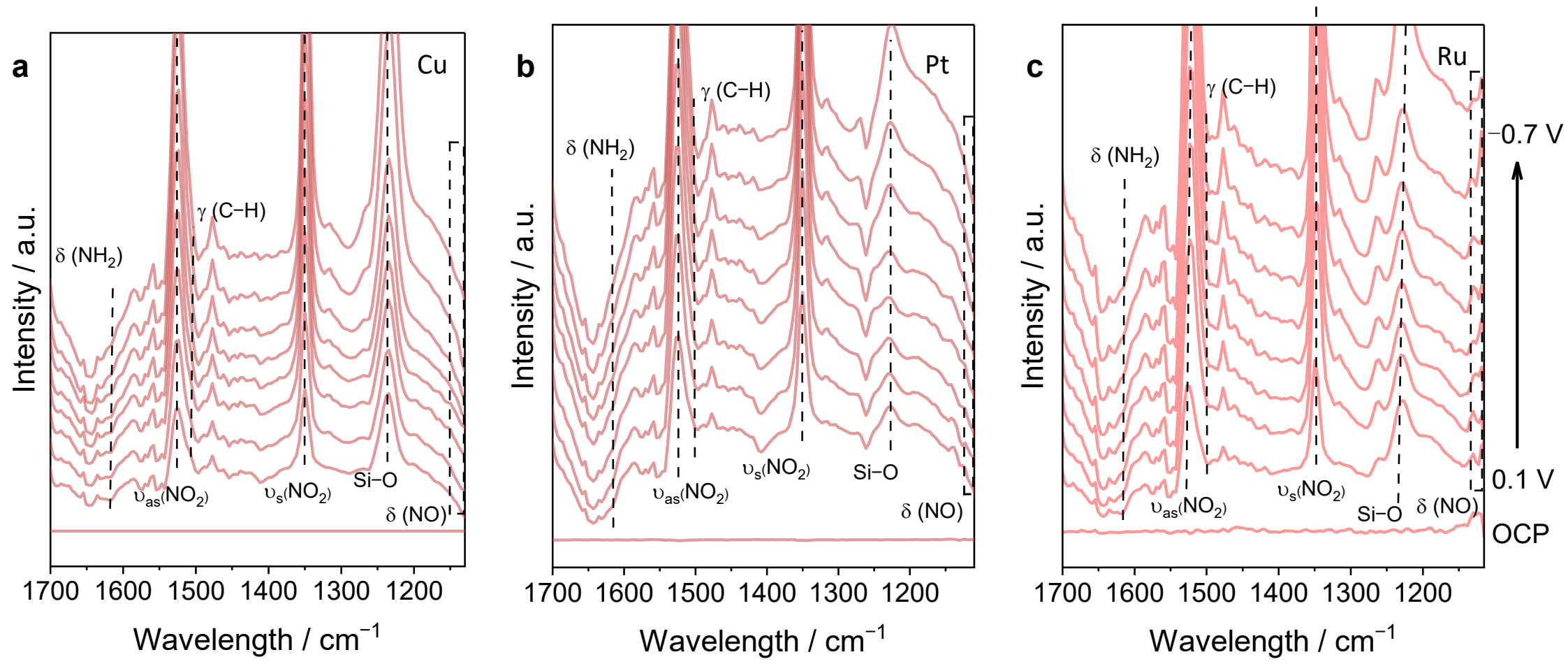

Supplementary Figure 11 In situ ATR-FTIR spectra under various bias potentials over Cu, Pt, and Ru. Source data for Fig. S11 are provided as a Source Data file.

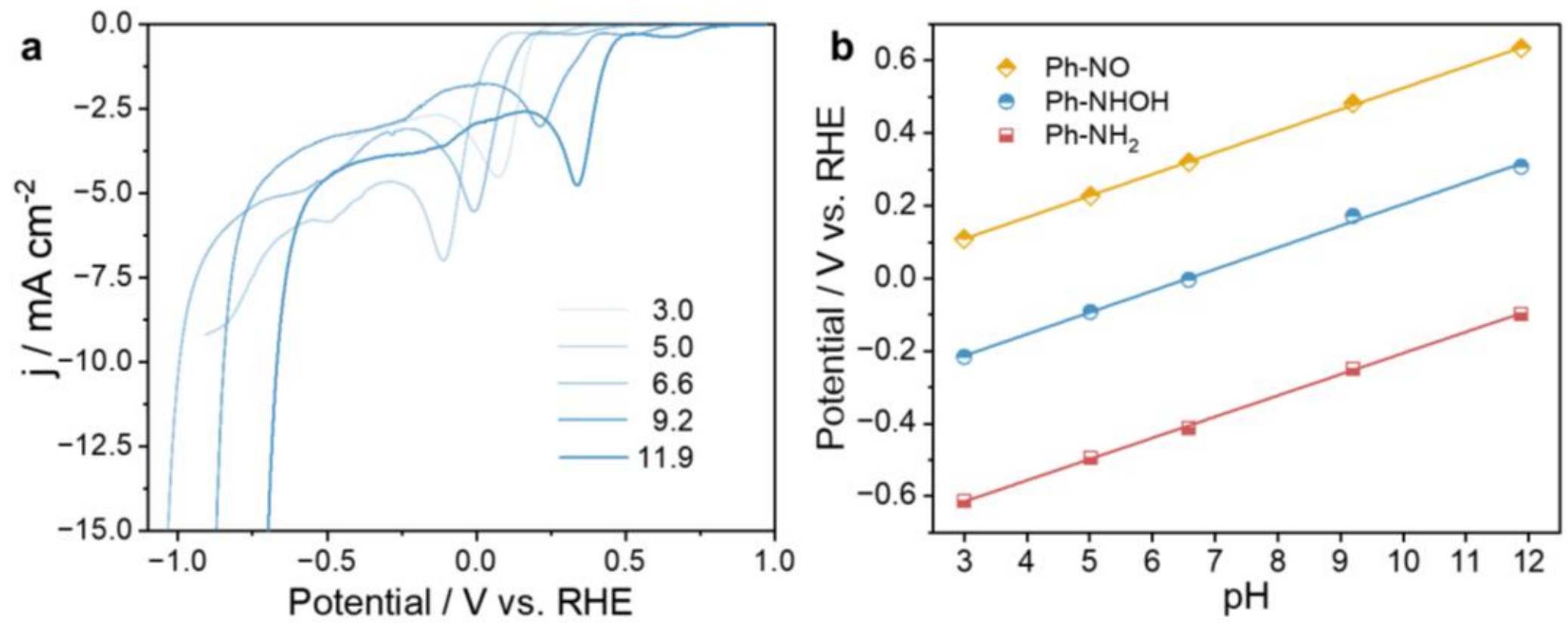

Supplementary Figure 12 a, LSV curves of the CV-Ag electrode under different pH conditions at 50 mV s^{-1}. **b,** Potentials of reduction peaks to form Ph-NO, Ph-NHOH, and Ph-NH_2 on CV-Ag as a function of pH. (The cosolvent dioxane was not used in the measurement of the PCET process on CV-Ag.) Source data for Fig. S12 are provided as a Source Data file.

Supplementary Note 4 LSV measurements were performed at five pH values to examine the influence of pH on the reaction pathway. The LSV curves and the pH dependence of the three reduction potentials are shown in Supplementary Figure 12b. The results revealed that the reduction peak potentials of Ph-NO, Ph-NHOH, and Ph-NH_2 on the Ag electrode shifted by 59 mV per unit increase in pH, confirming that the reductive hydrogenation of Ph-NO_2 proceeded via a sequential proton-coupled electron transfer (PCET) pathway.

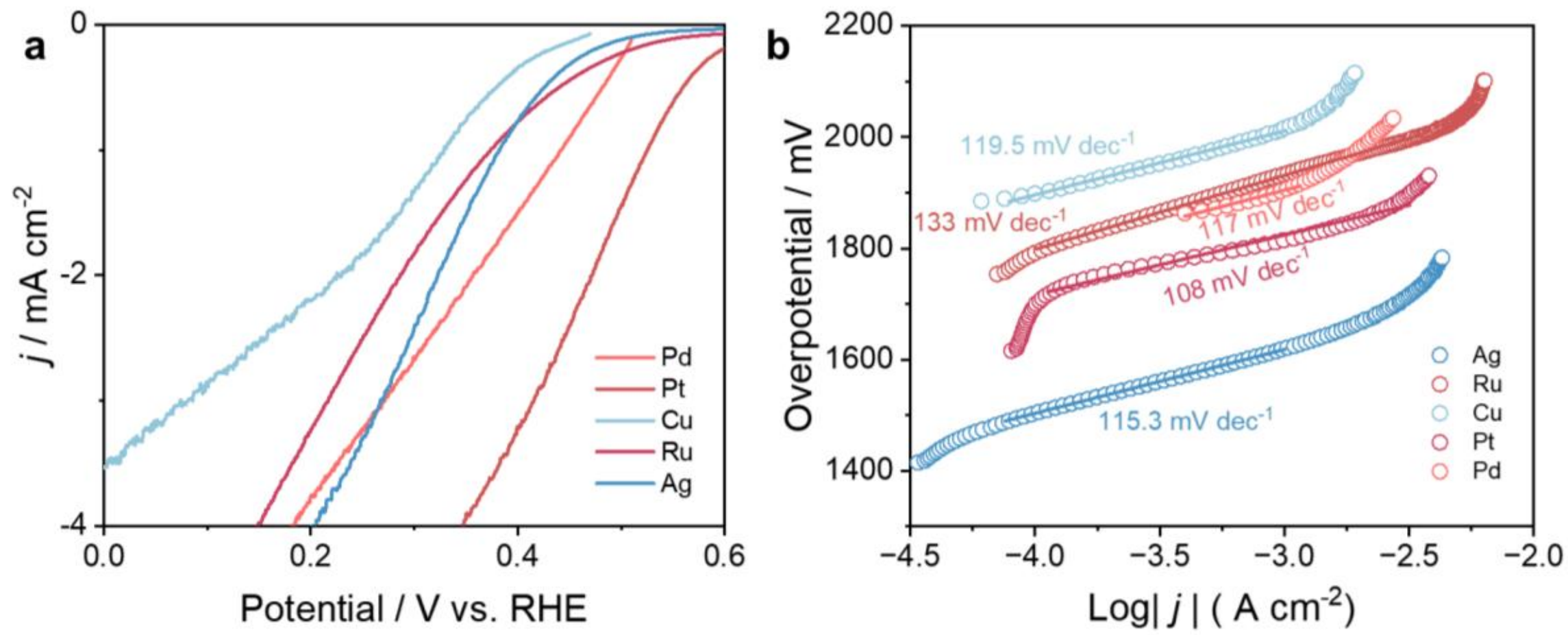

Supplementary Figure 13 **a**, LSV curves of five metals at 10 mV s^{-1}. **b**, The corresponding Tafel plots. Source data for Fig. S13 are provided as a Source Data file.

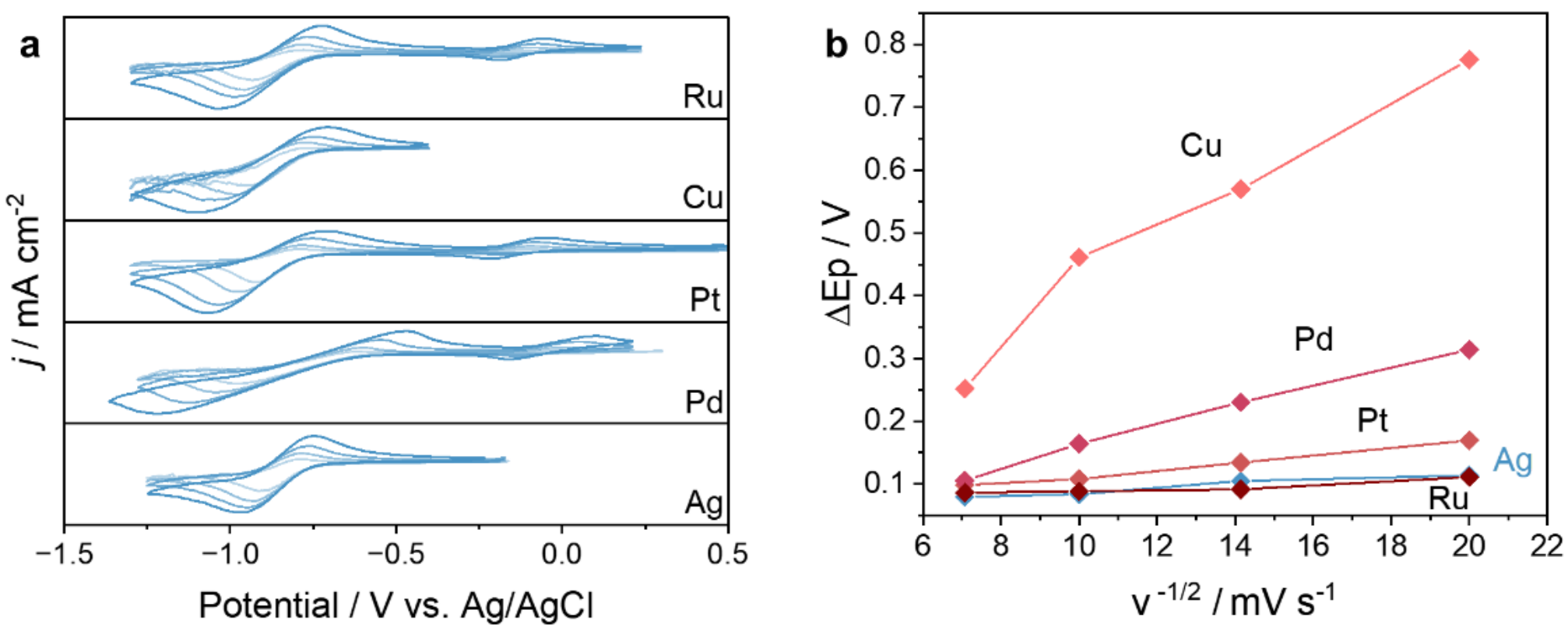

Supplementary Figure 14 a, CV curves of five metals in aprotic solvent (acetonitrile + 0.1 M n-Bu_4NPF_6 + 10 mM Ph-NO). **b**, Peak potential difference (ΔEp) as a function of the scan rate for five different metal electrodes. Source data for Fig. S14 are provided as a Source Data file.

Supplementary Note 5 The heterogeneous electron transfer kinetics of the target redox process in the aprotic organic medium were systematically investigated via variable-scan-rate cyclic voltammetry[5].

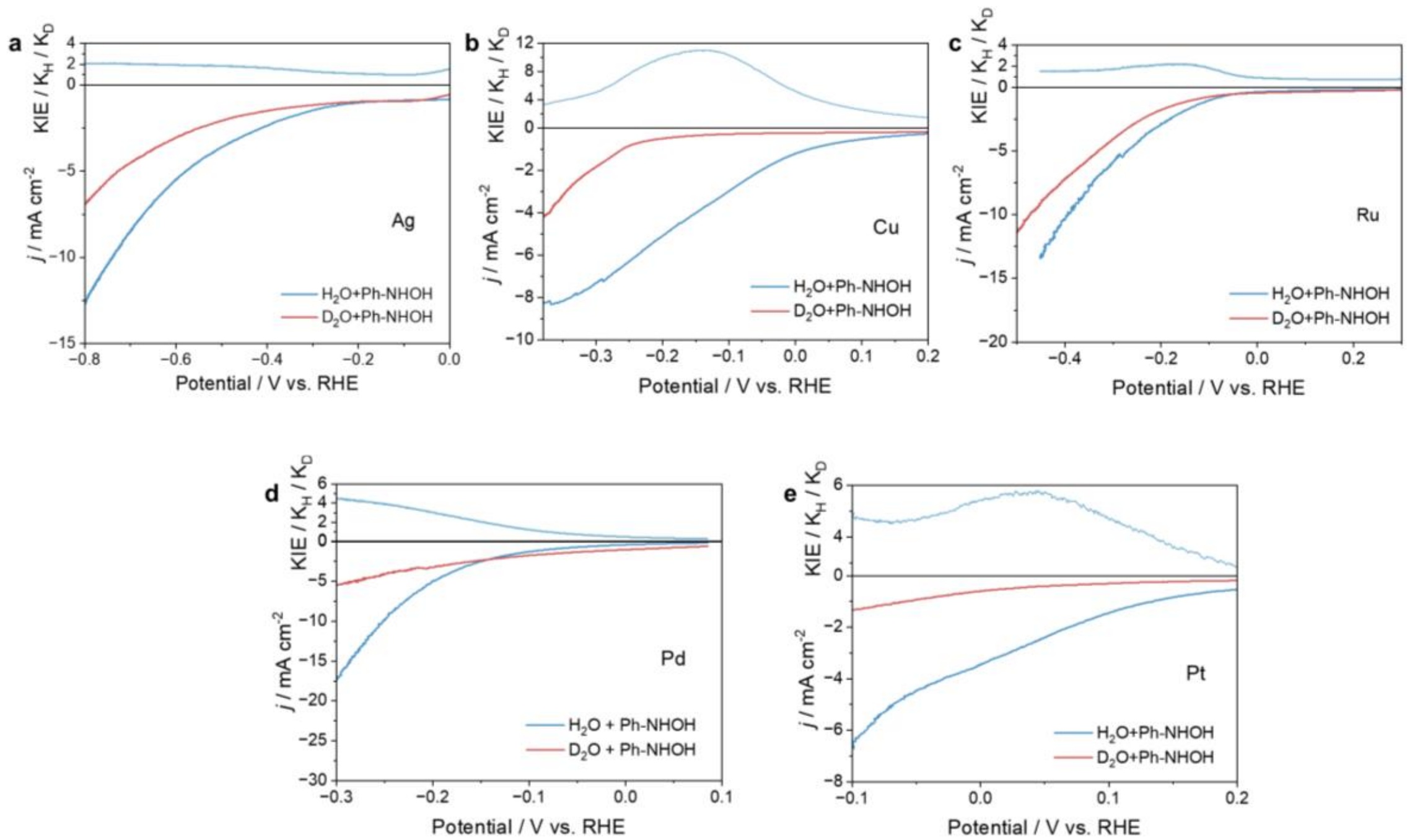

Supplementary Figure 15 KIE of H/D over five metal electrodes at 10 mV s^{-1}. Source data for Fig. S15 are provided as a Source Data file.

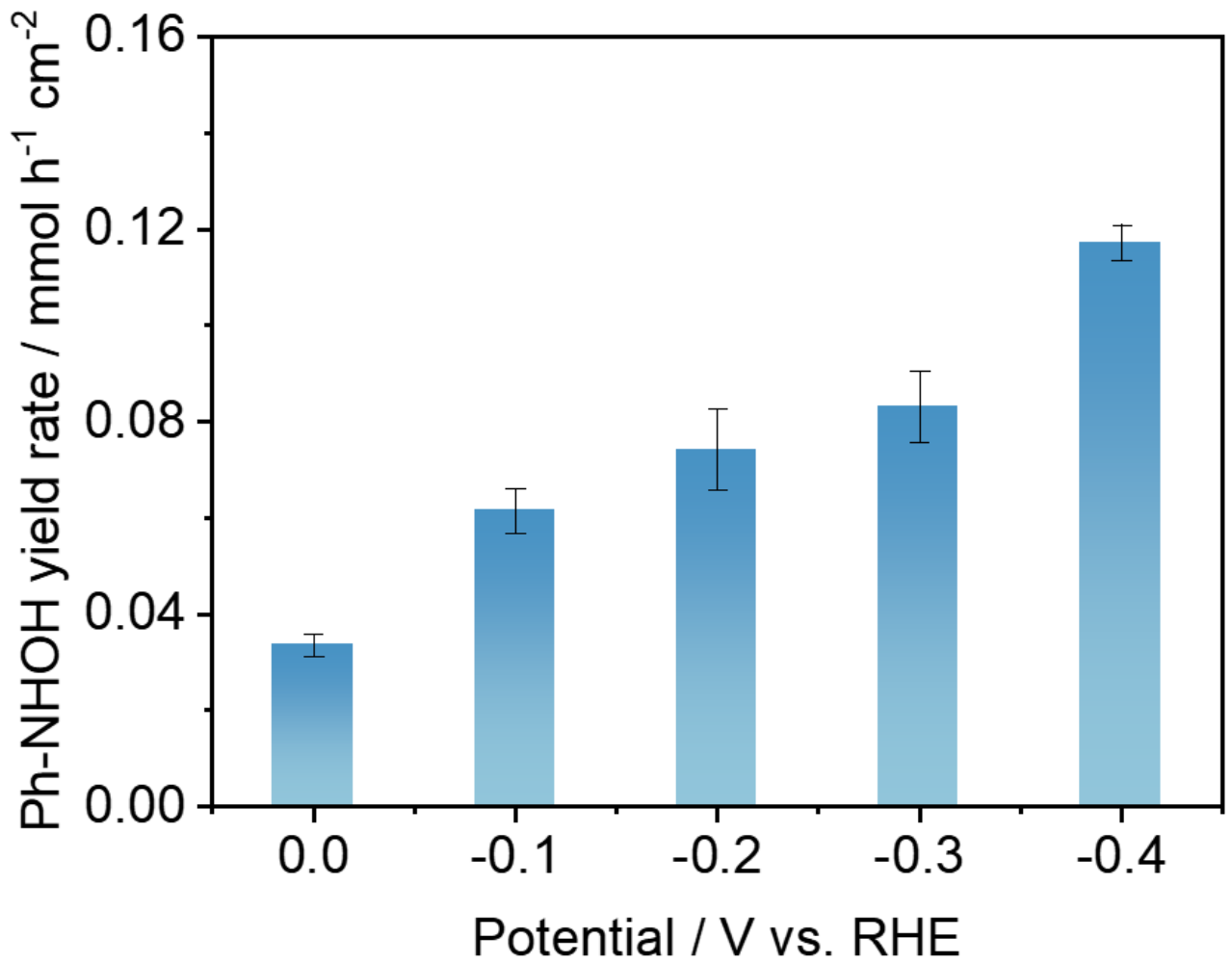

Supplementary Figure 16 Ph-NHOH yield rates over CV-Ag at different potentials.

Source data for Fig. S16 are provided as a Source Data file.

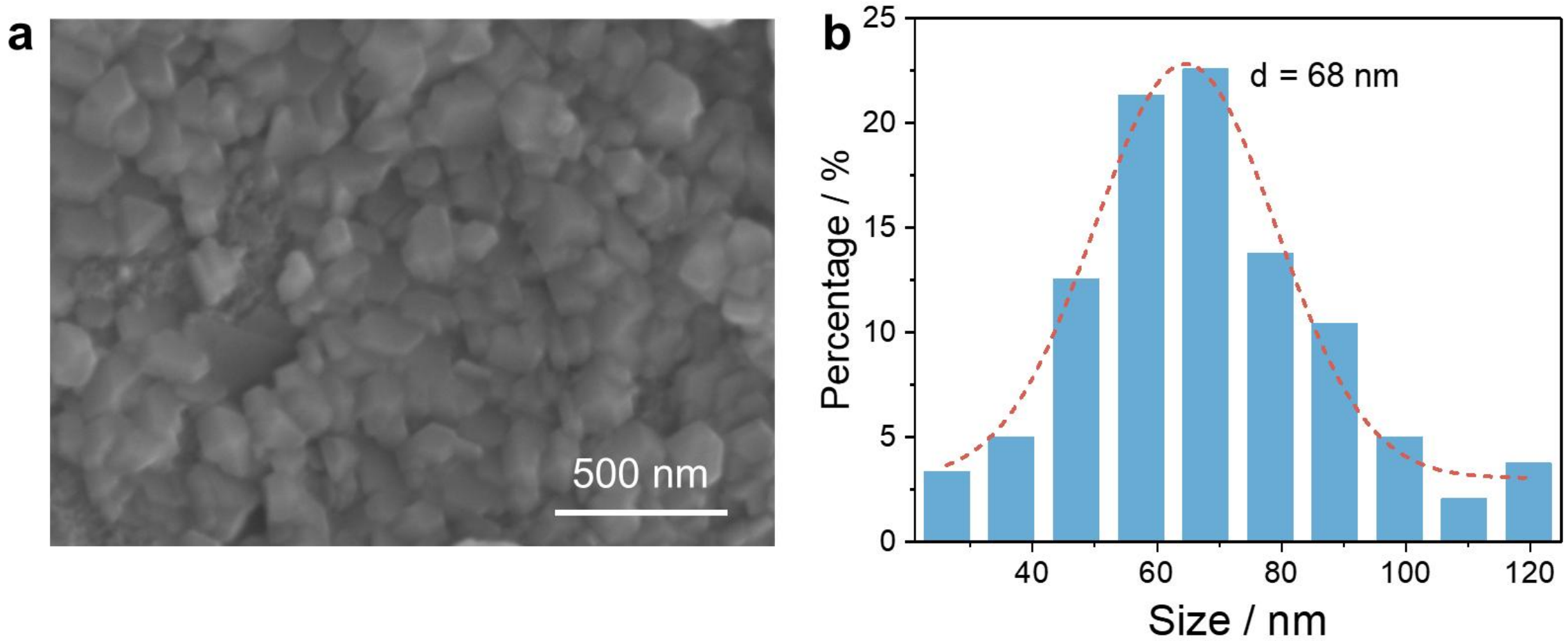

Supplementary Figure 17 a, SEM image of CV-Ag. **b**, Corresponding particle size distribution. Source data for Fig. S17 are provided as a Source Data file.

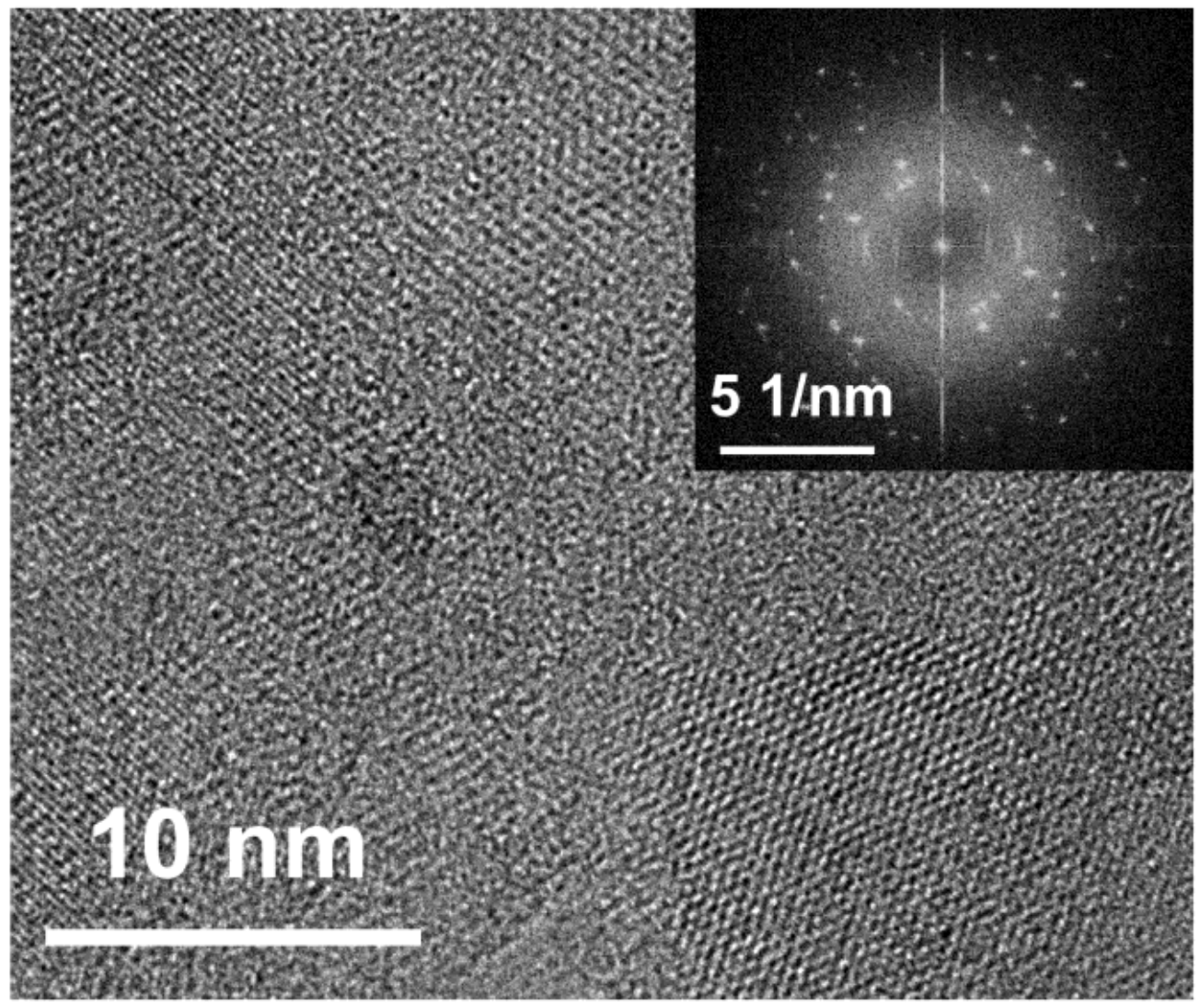

Supplementary Figure 18 HRTEM image and corresponding selected area electron diffraction image of CV-Ag. Source data for Fig. S18 are provided as a Source Data file.

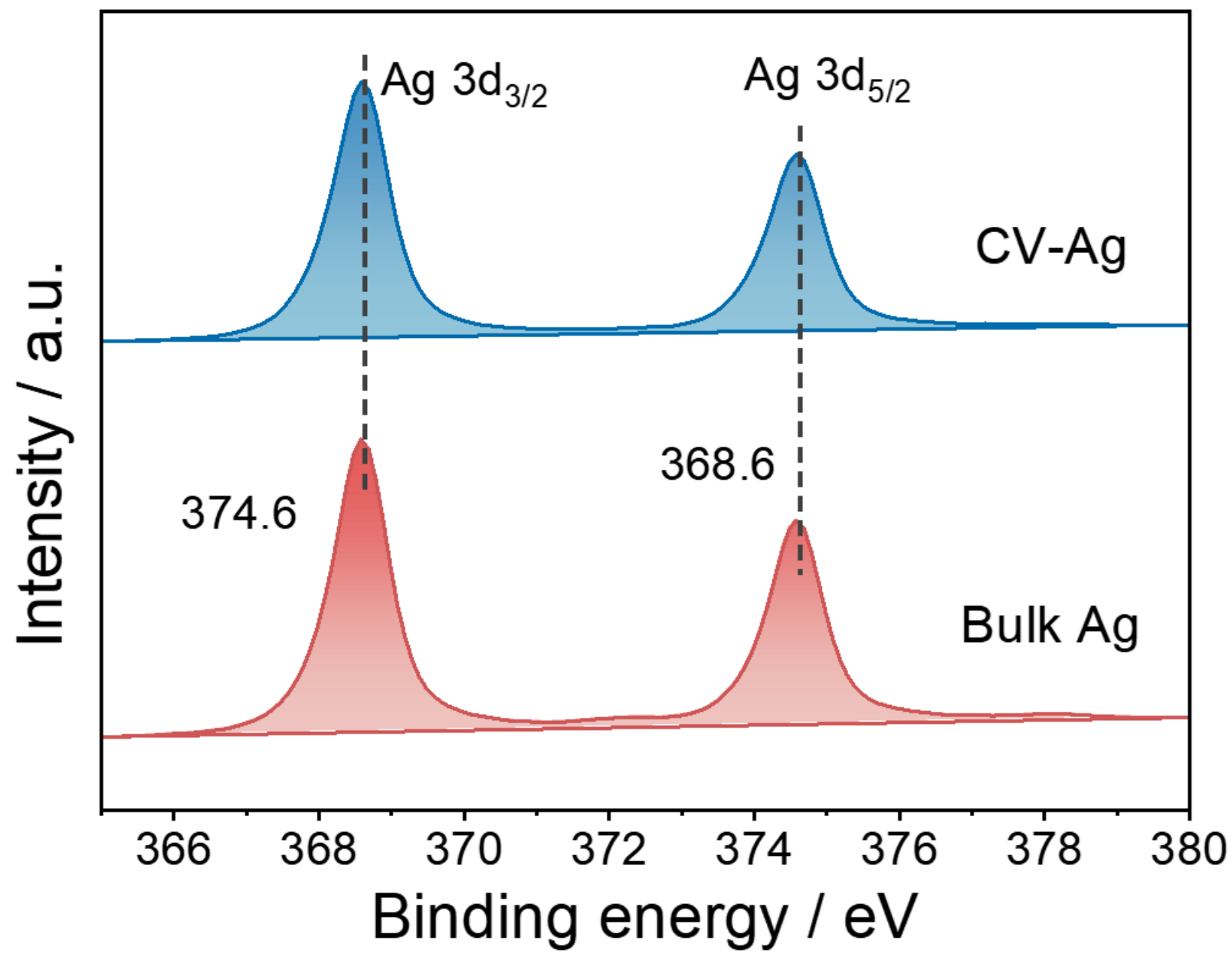

Supplementary Figure 19 XPS spectra of CV-Ag and bulk Ag. Source data for Fig. S19 are provided as a Source Data file.

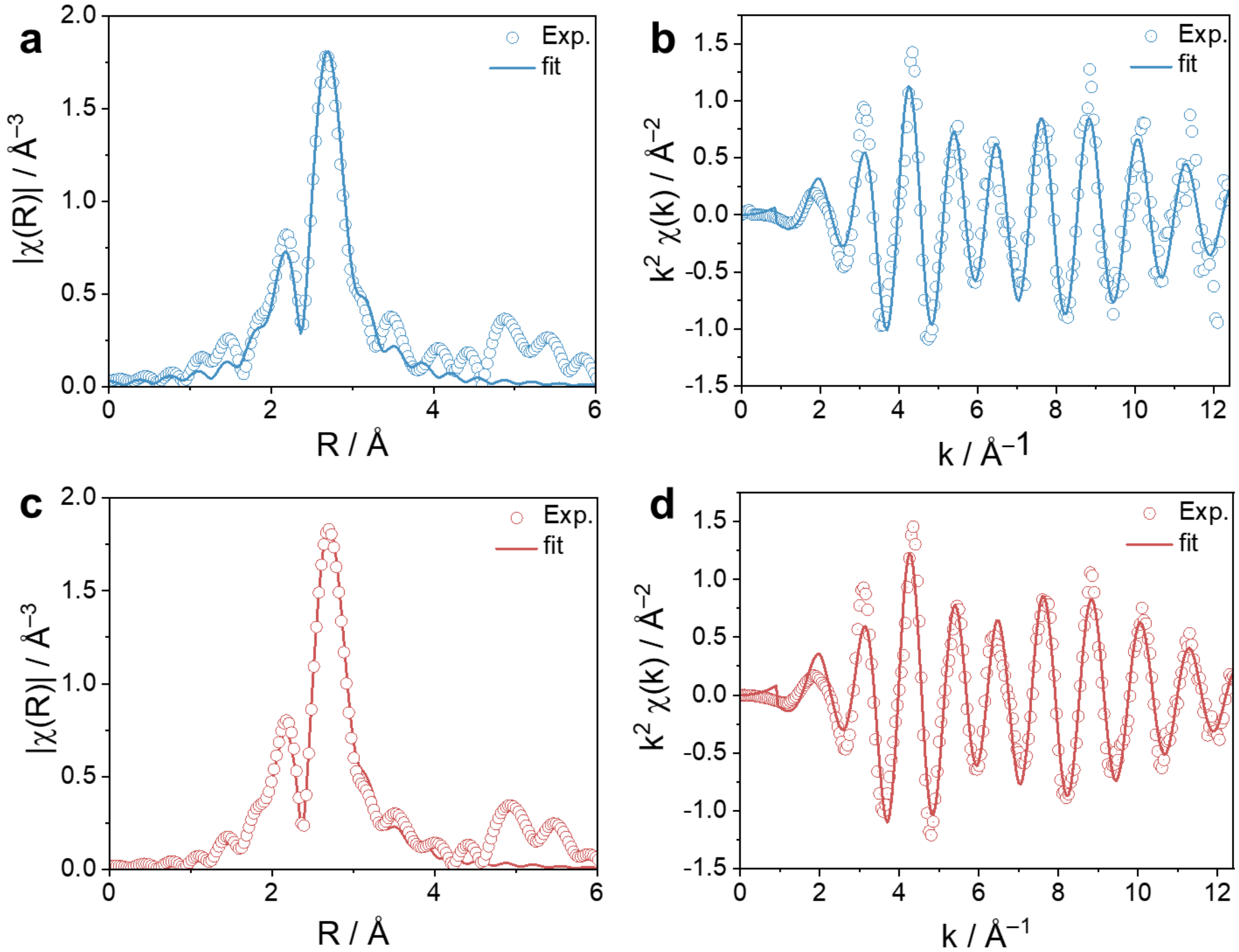

Supplementary Figure 20 k- and R-space fitting results at the Ag K-edge. a-b, CV-Ag. **c-d,** Bulk Ag. Source data for Fig. S20 are provided as a Source Data file.

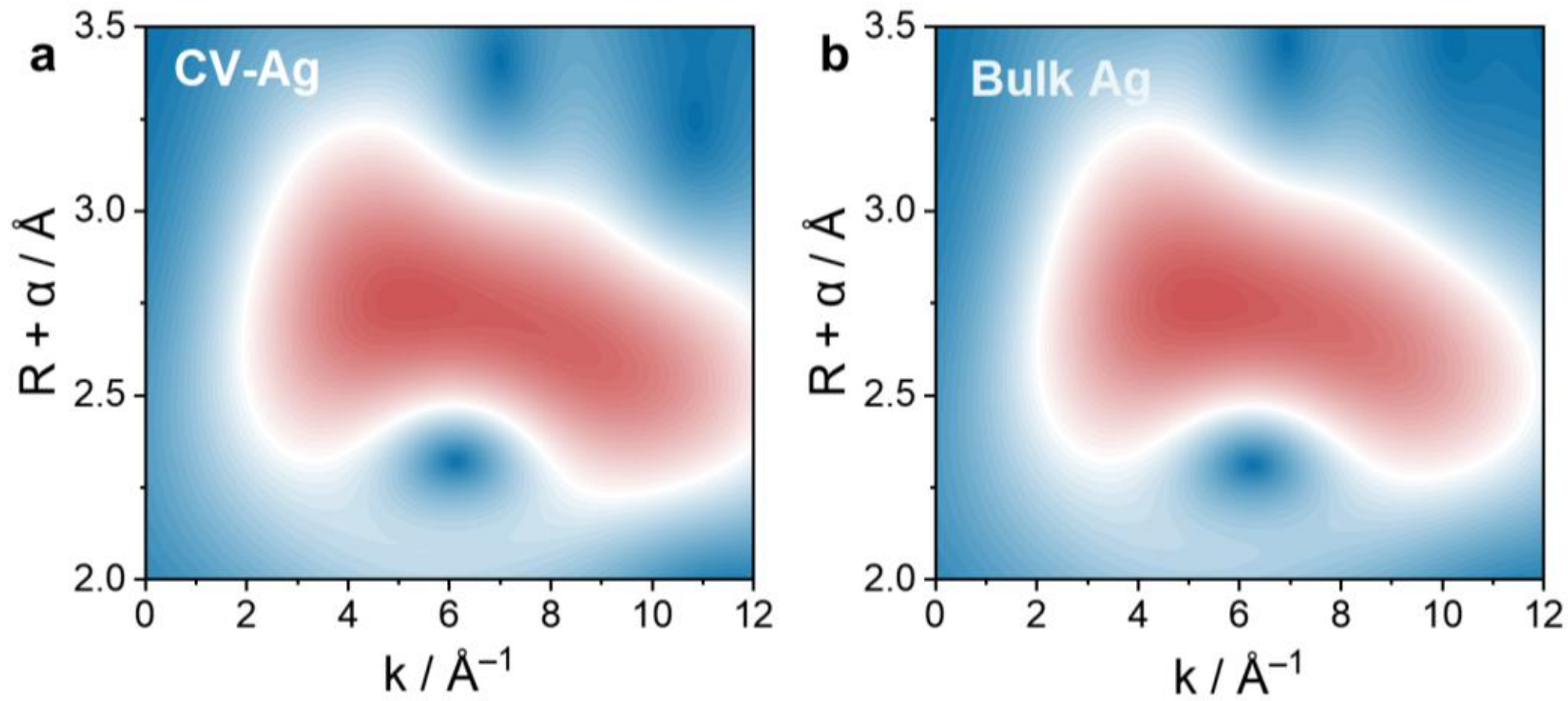

Supplementary Figure 21 Plots of the continuous Cauchy wavelet transform (WT) spectra of CV-Ag and bulk Ag. Source data for Fig. S21 are provided as a Source Data file.

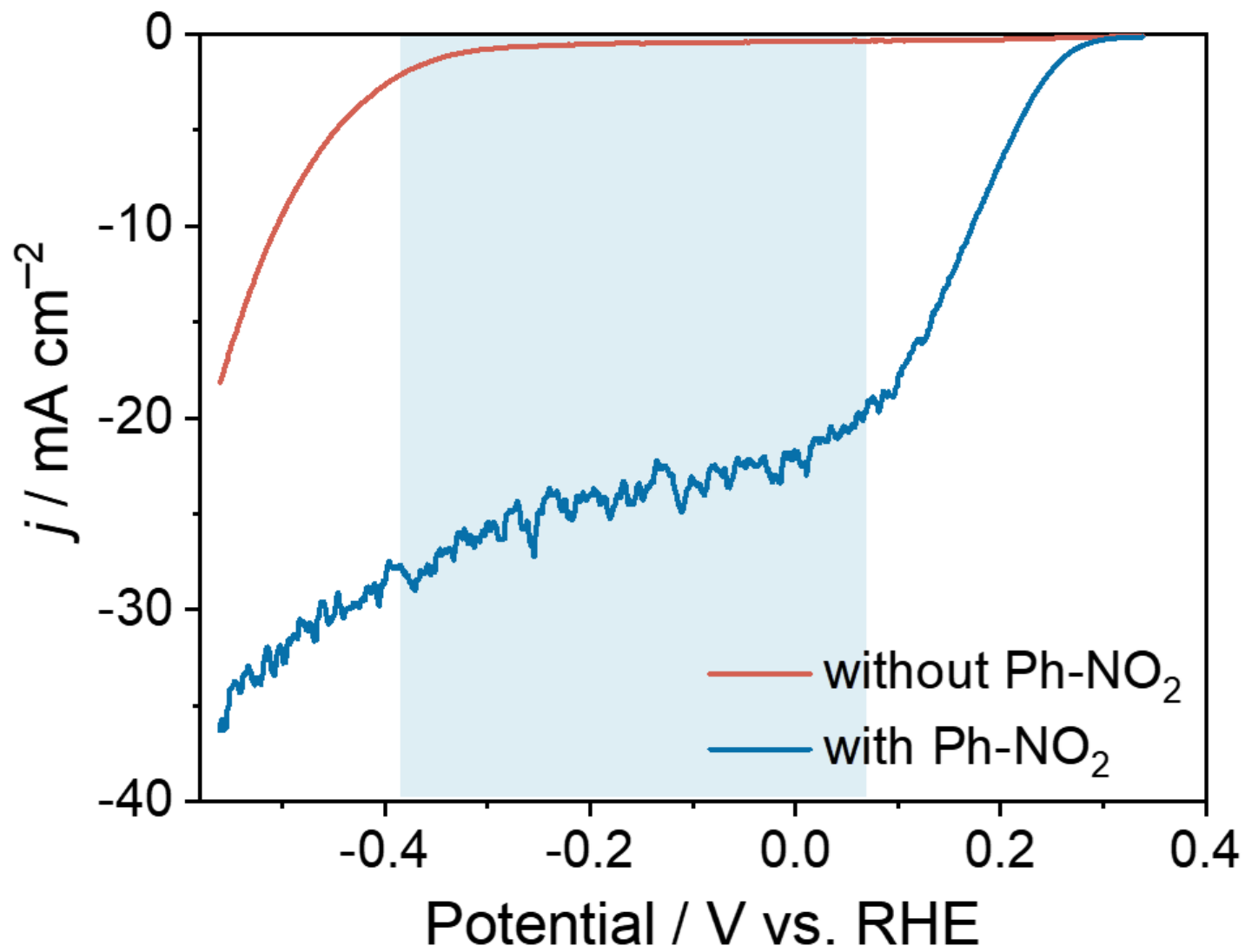

Supplementary Figure 22 LSV curves of CV-Ag in a 1.0 M phosphate solution with and without 0.2 mM Ph-NO_2 at 10 mV s^{-1}. Source data for Fig. S22 are provided as a Source Data file.

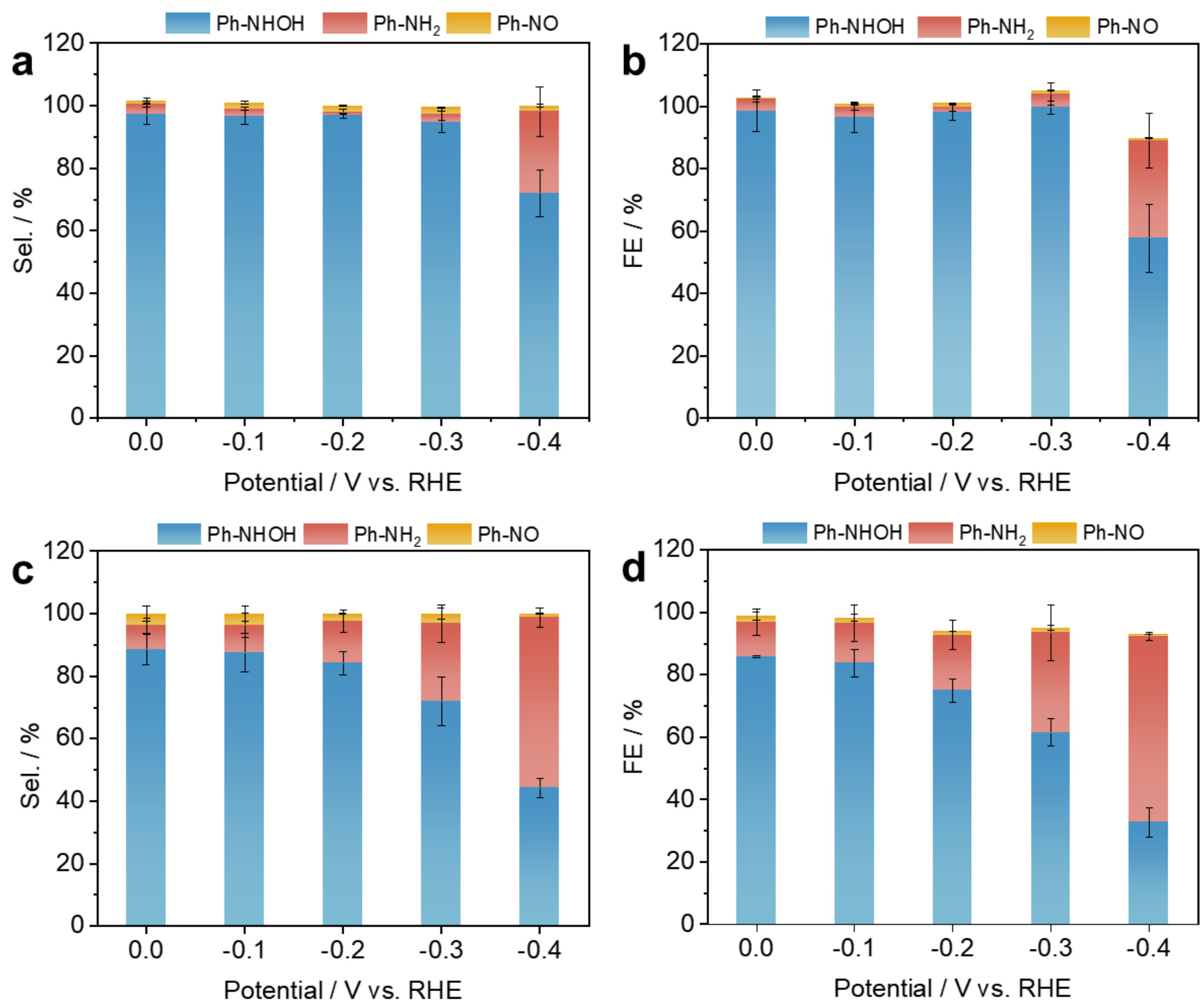

Supplementary Figure 23 Product distributions over different metals in phosphate solution. a-b, CV-Ag, **c-d,** Bulk Ag. Source data for Fig. S23 are provided as a Source Data file.

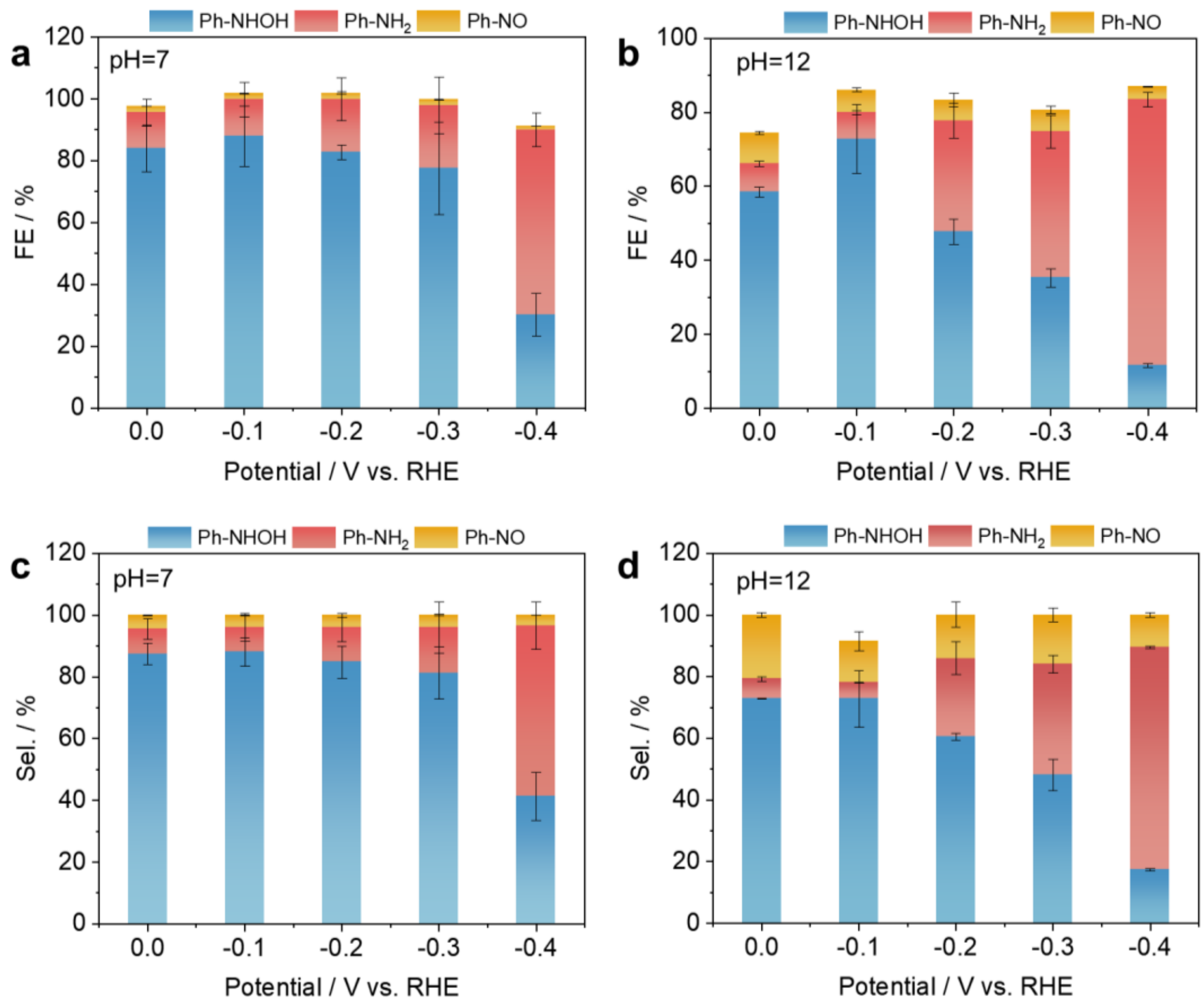

Supplementary Figure 24 Product distribution over the CV-Ag electrodes in neutral (pH=7) and alkaline (pH=12) electrolytes. Source data for Fig. S24 are provided as a Source Data file.

Supplementary Note 6 The detailed product distributions at various pH values and potentials revealed that the formation of Ph-NHOH decreased with increasing pH.

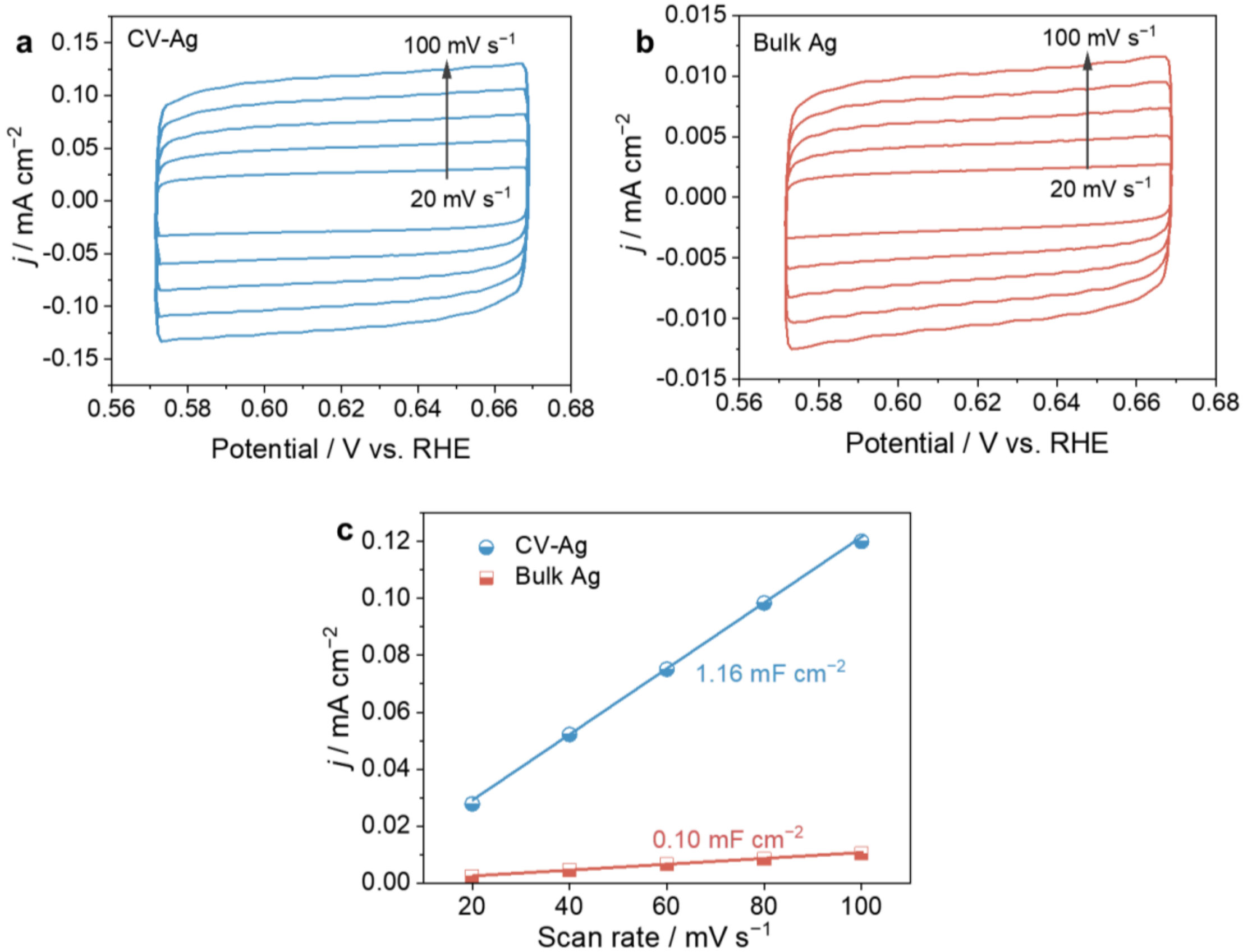

Supplementary Figure 25 CV curves in 0.5 M phosphate solution over **a,** CV-Ag, **b,** and bulk-Ag. **c,** Linear fitting of the capacitive current versus scan rate for CV-Ag and bulk-Ag. Source data for Fig. S25 are provided as a Source Data file.

Supplementary Note 7 Electrochemical double-layer capacitance (C_{dl}) was determined by measuring CV curves in a potential window without the Faradaic process across a range of scan rates.[6] A linear relationship is observed when the current density at a given potential is plotted versus the scan rate, and the slope is C_{dl}. Assuming a constant Cs, the electrochemical surface area (ECSA) is directly proportional to C_{dl}, enabling a relative comparison of the ECSA values from the measured C_{dl}.

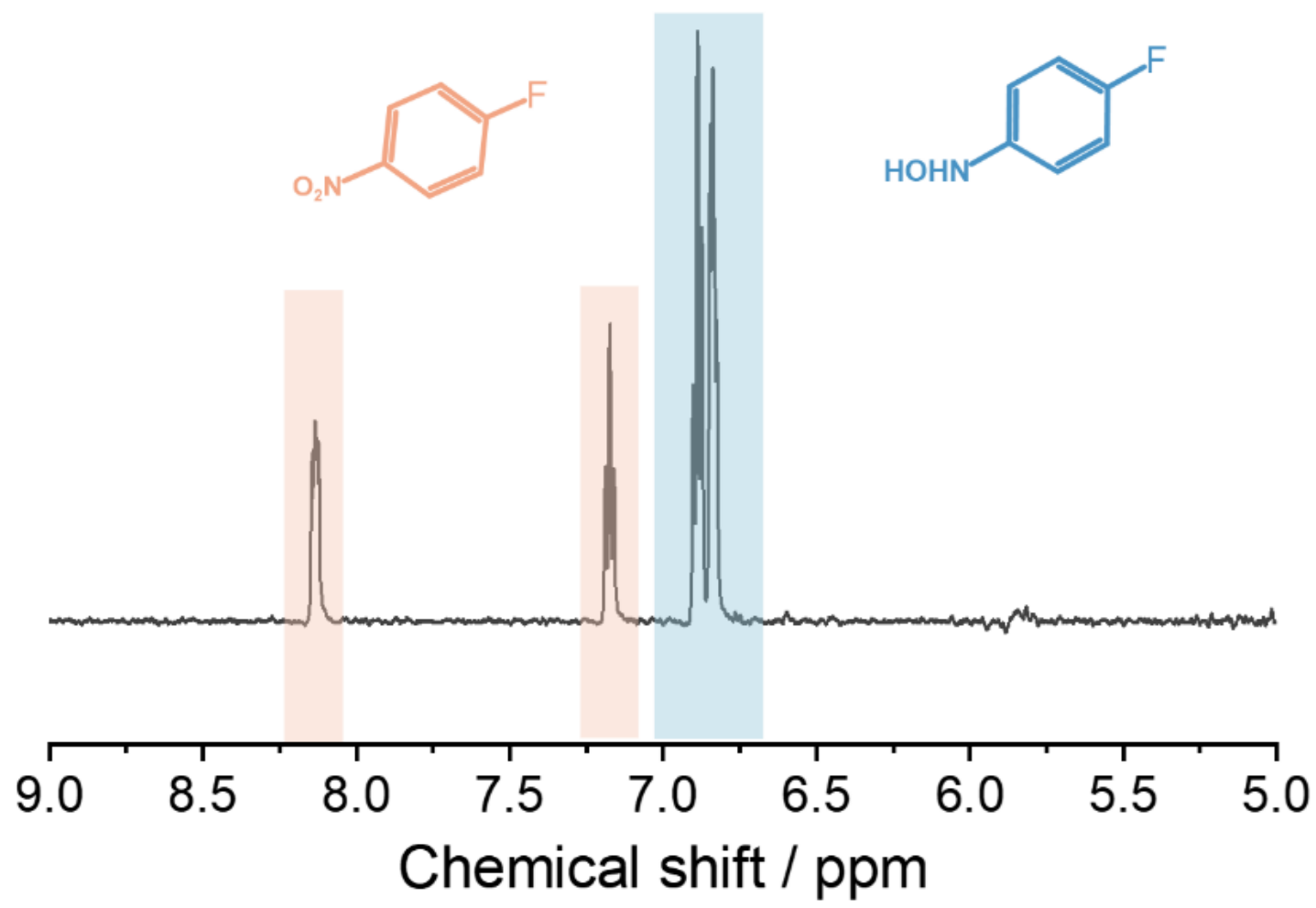

Supplementary Figure 26 ^{1}H NMR spectra of the crude solution obtained after electroreduction of para-F-substituted nitrobenzene. Source data for Fig. S26 are provided as a Source Data file.

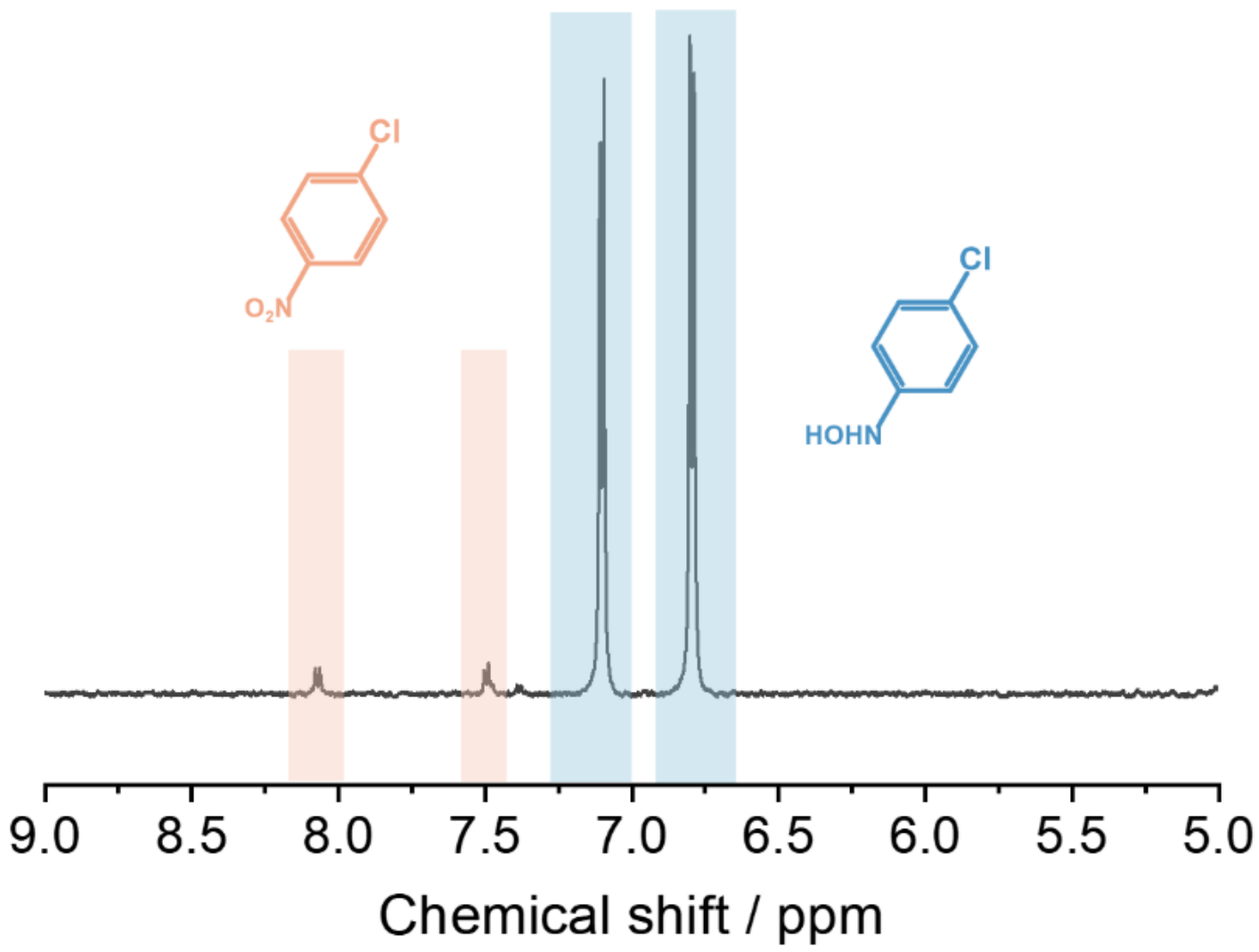

Supplementary Figure 27 ^{1}H NMR spectra of the crude solution obtained after electroreduction of para-Cl-substituted nitrobenzene. Source data for Fig. S27 are provided as a Source Data file.

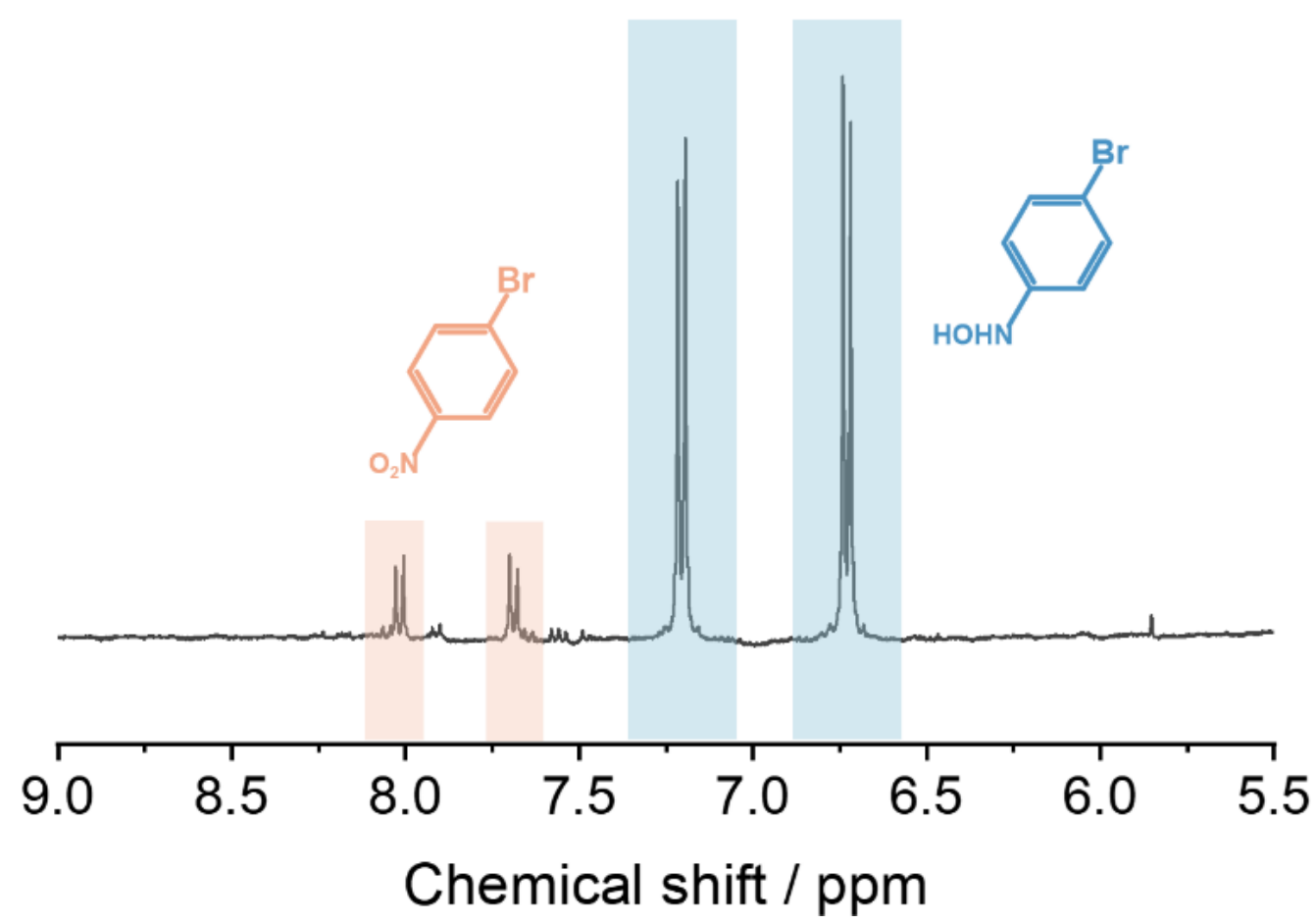

Supplementary Figure 28 ^{1}H NMR spectra of the crude solution obtained after electroreduction of para-Br-substituted nitrobenzene. Source data for Fig. S28 are provided as a Source Data file.

.

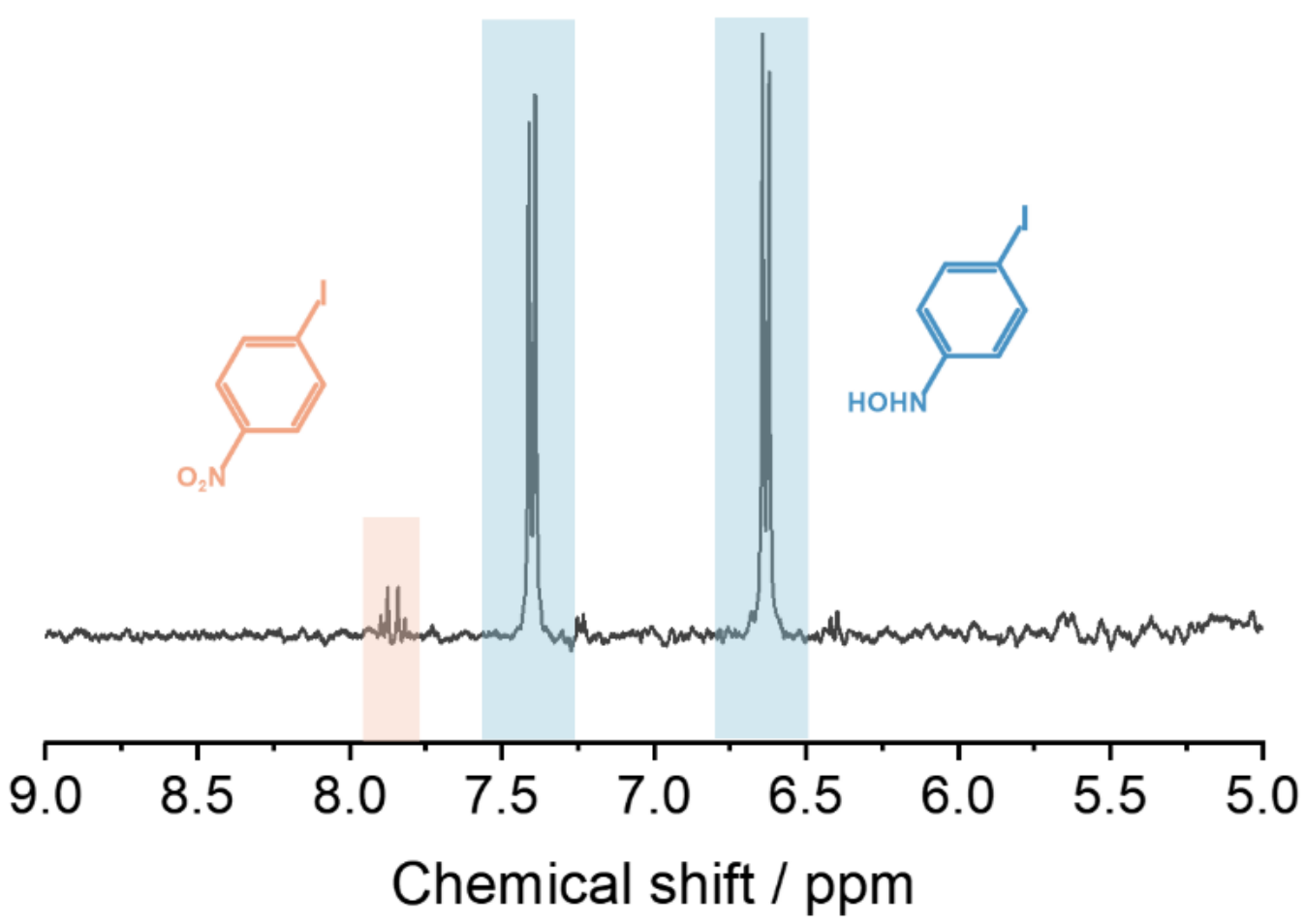

Supplementary Figure 29 ^{1}H NMR spectra of the crude solution obtained after electroreduction of para-I-substituted nitrobenzene. Source data for Fig. S29 are provided as a Source Data file.

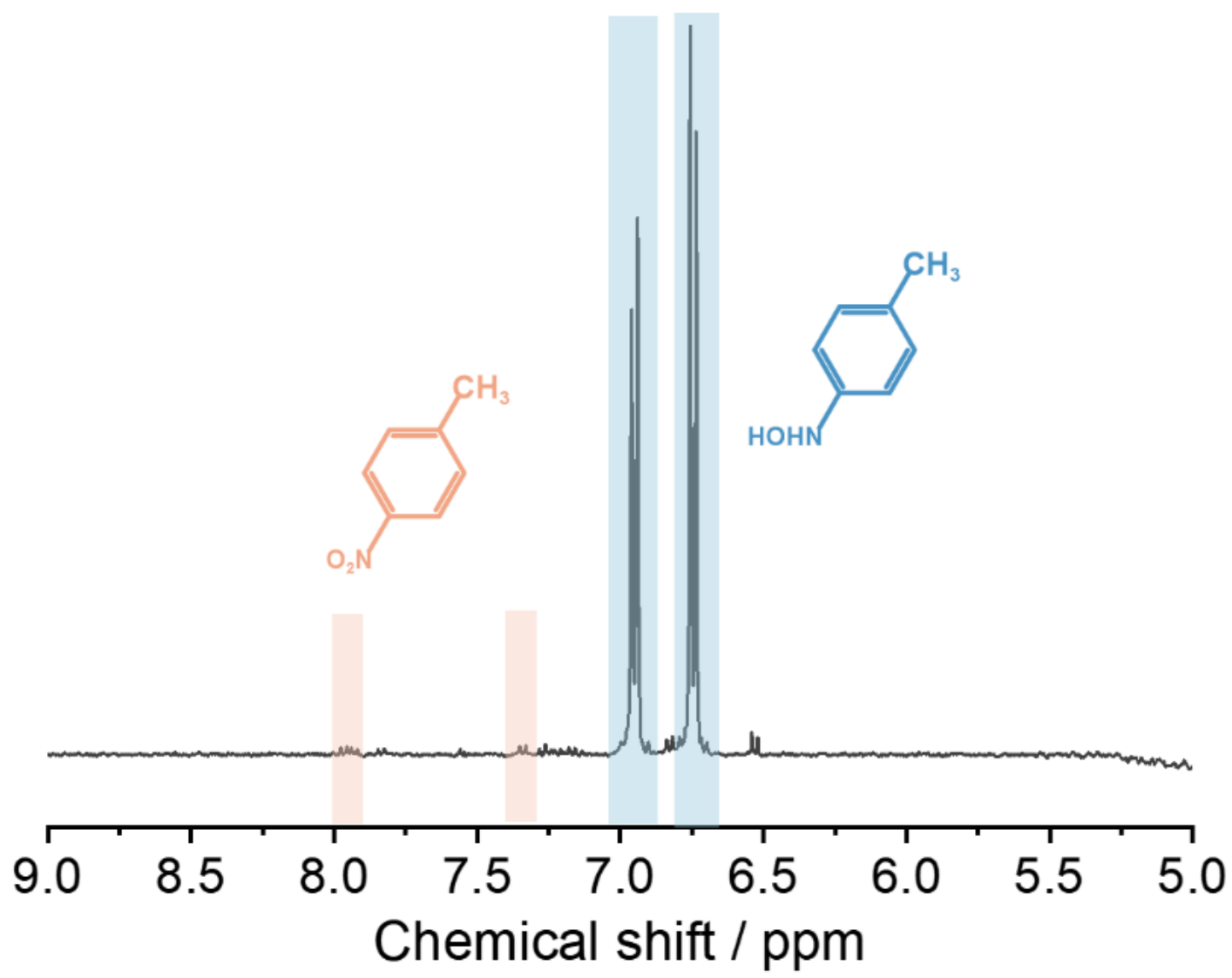

Supplementary Figure 30 ^{1}H NMR spectra of the crude solution obtained after electroreduction of para-methyl-substituted nitrobenzene. Source data for Fig. S30 are provided as a Source Data file.

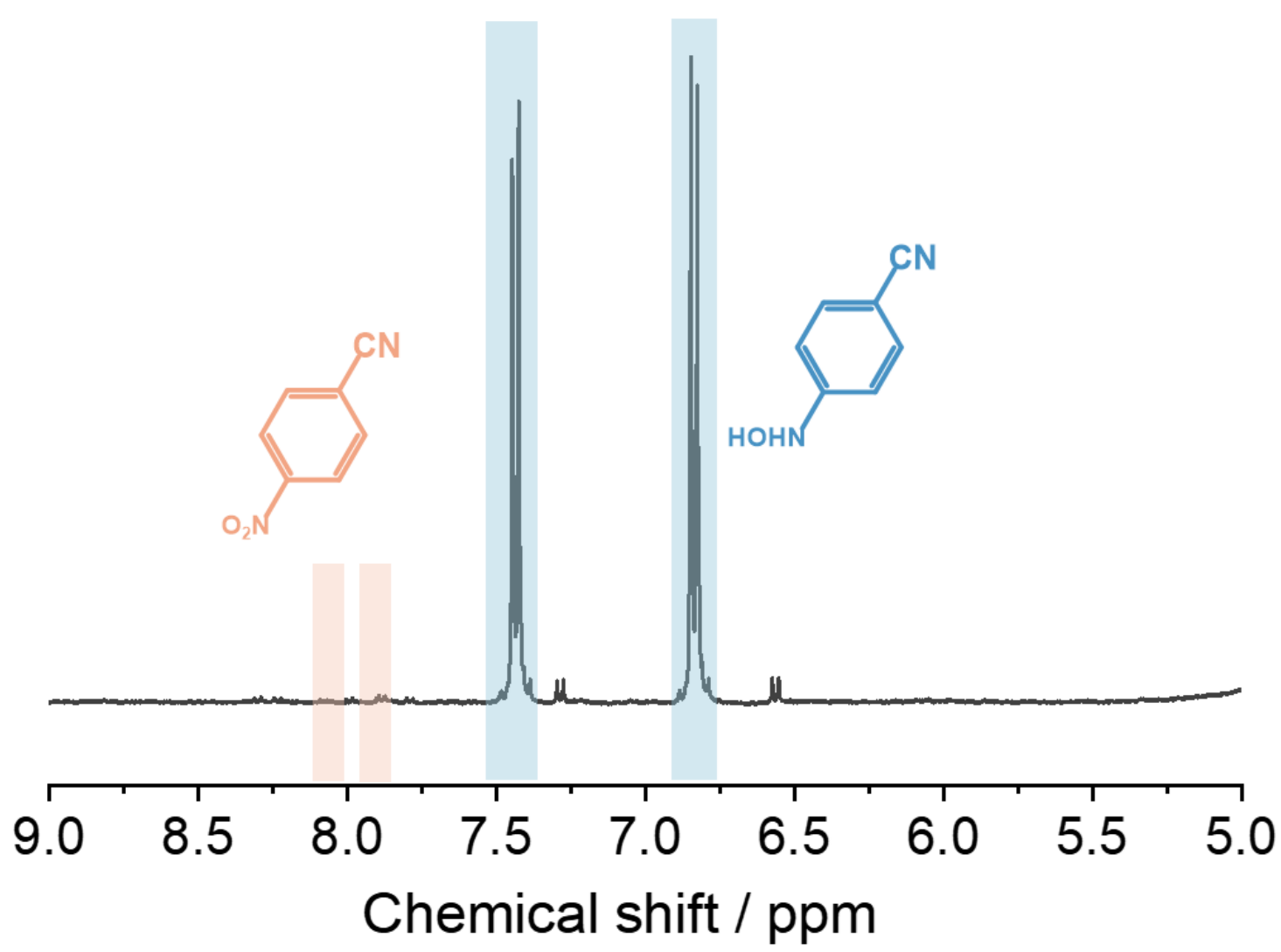

Supplementary Figure 31 ^{1}H NMR spectra of the crude solution obtained after the electroreduction of para-cyan-substituted nitrobenzene. Source data for Fig. S31 are provided as a Source Data file.

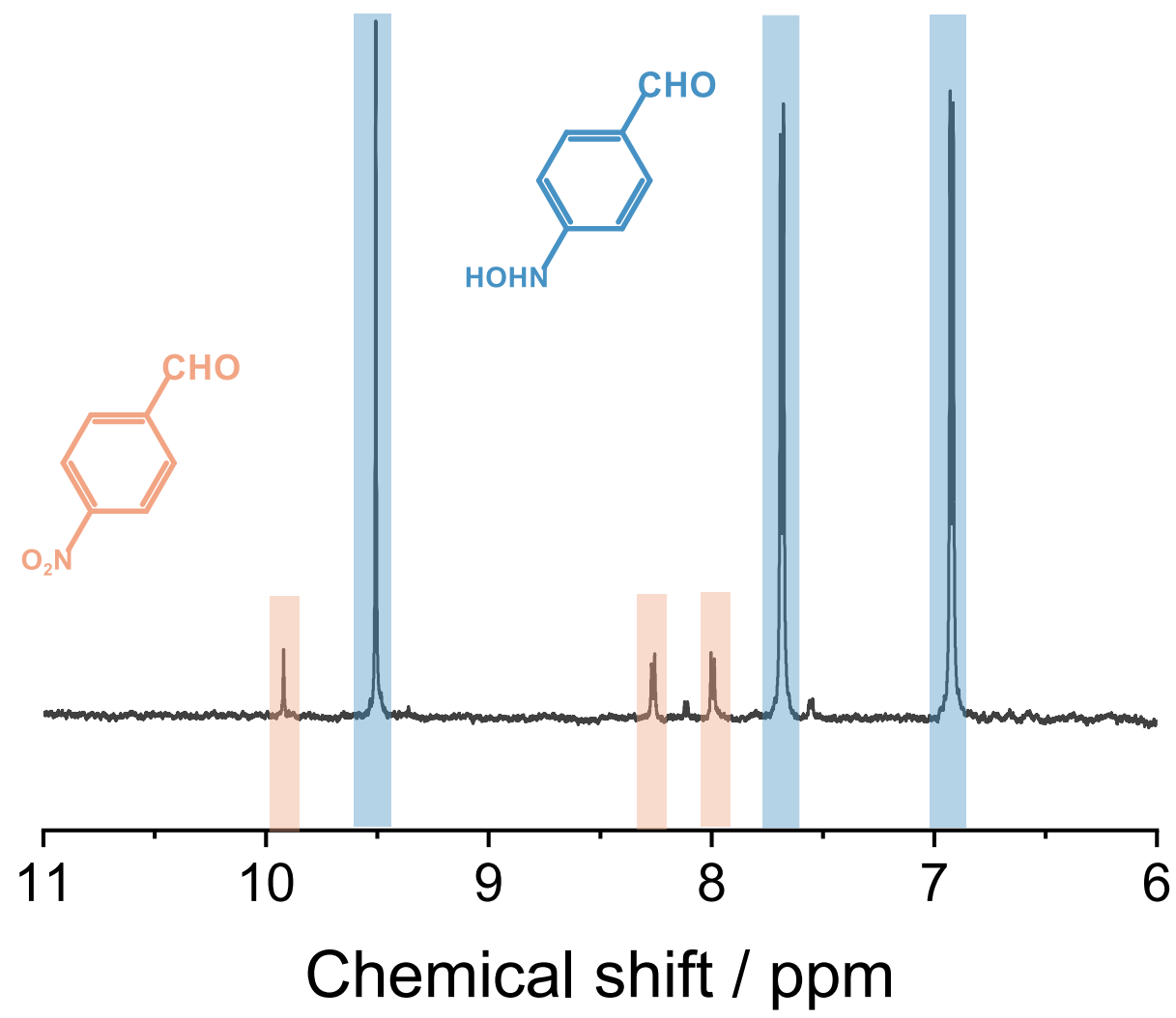

Supplementary Figure 32 ^{1}H NMR spectra of the crude solution obtained after electroreduction of para-formyl-substituted nitrobenzene. Source data for Fig. S32 are provided as a Source Data file.

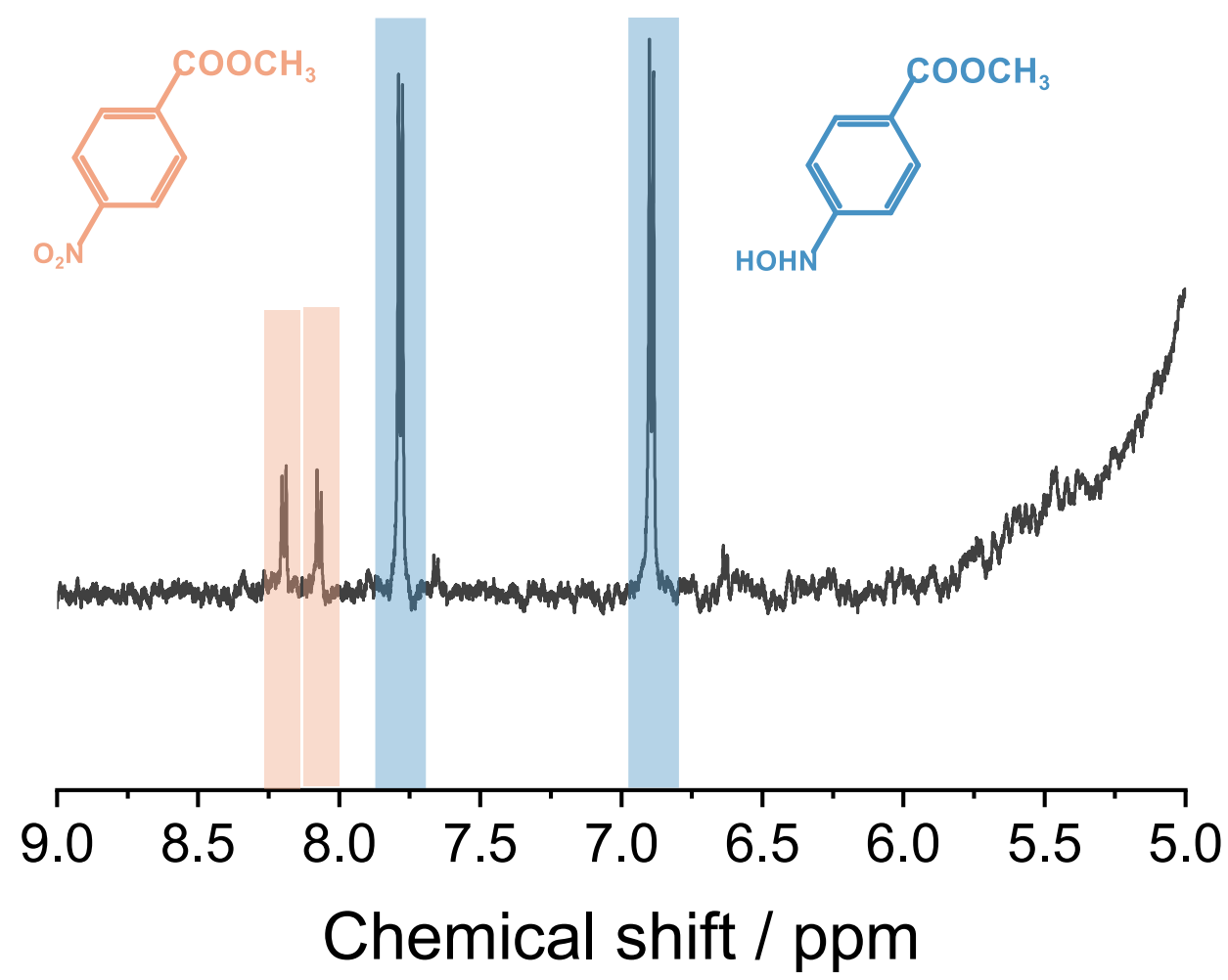

Supplementary Figure 33 ^{1}H NMR spectra of the crude solution obtained after electroreduction of para-ester-substituted nitrobenzene. Source data for Fig. S33 are provided as a Source Data file.

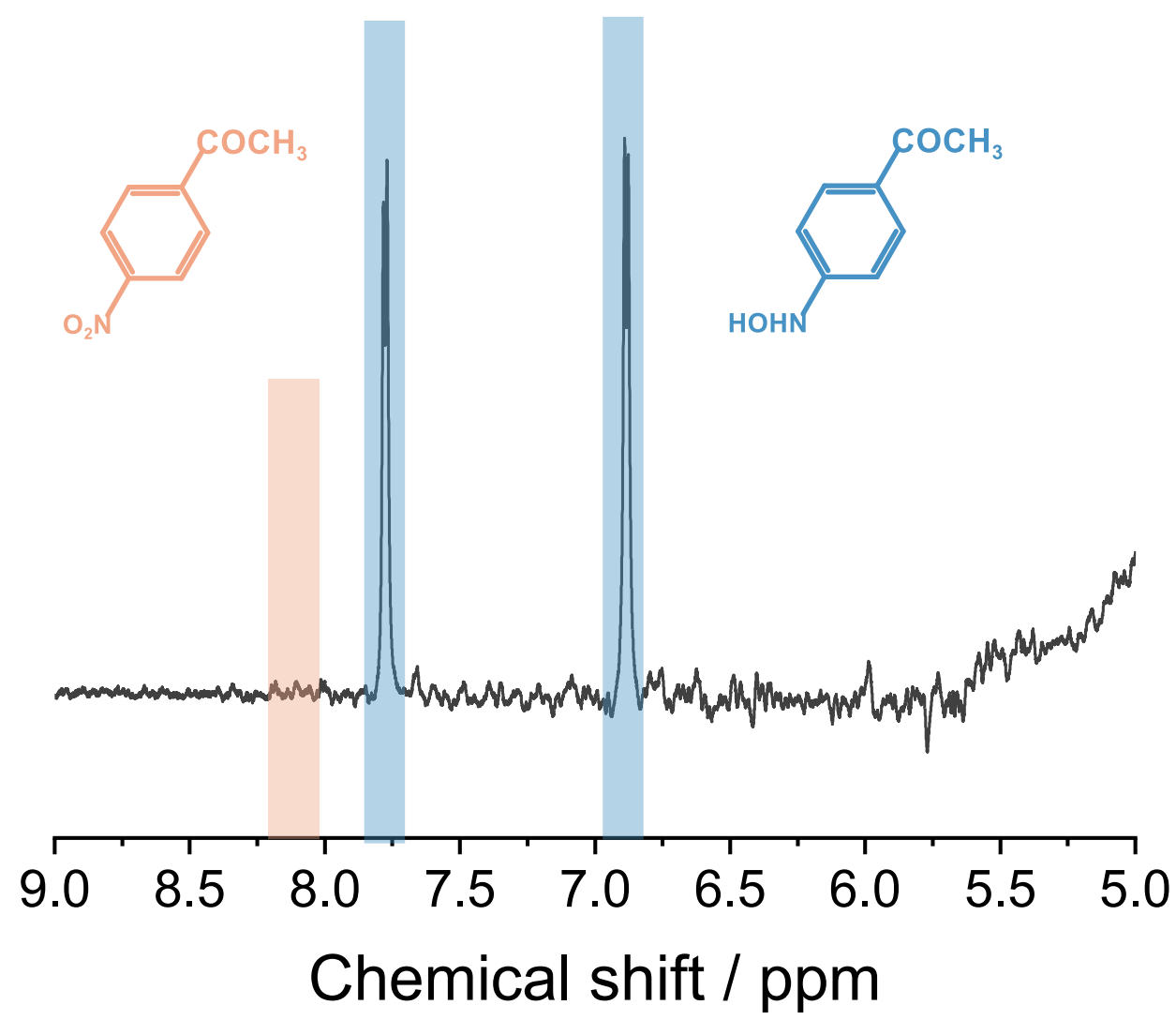

Supplementary Figure 34 ^{1}H NMR spectra of the crude solution obtained after electroreduction of para-keto-substituted nitrobenzene. Source data for Fig. S34 are provided as a Source Data file.

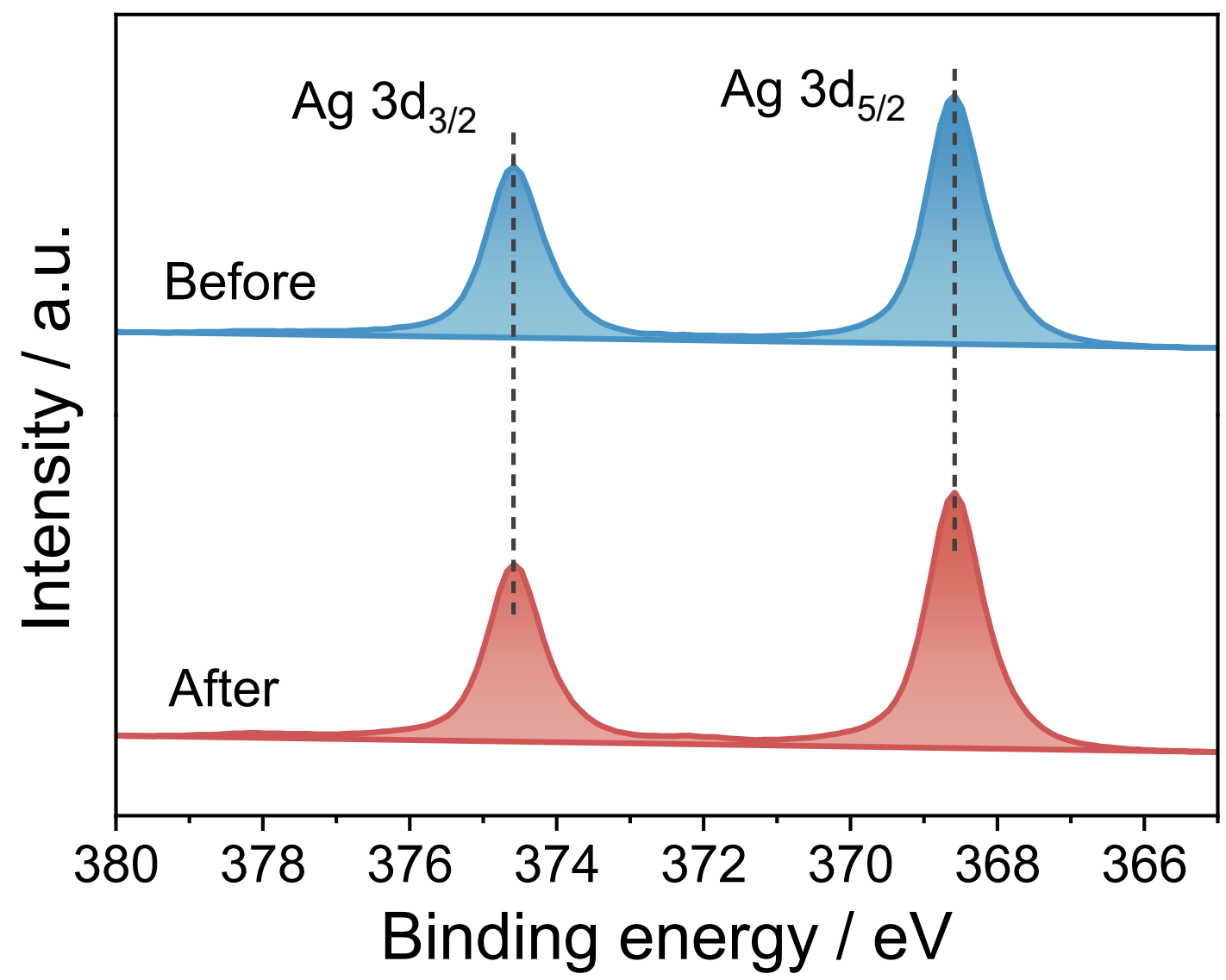

Supplementary Figure 35 Ag 3d XPS spectra of the CV-Ag electrode before and after long-term electrolysis. Source data for Fig. S35 are provided as a Source Data file.

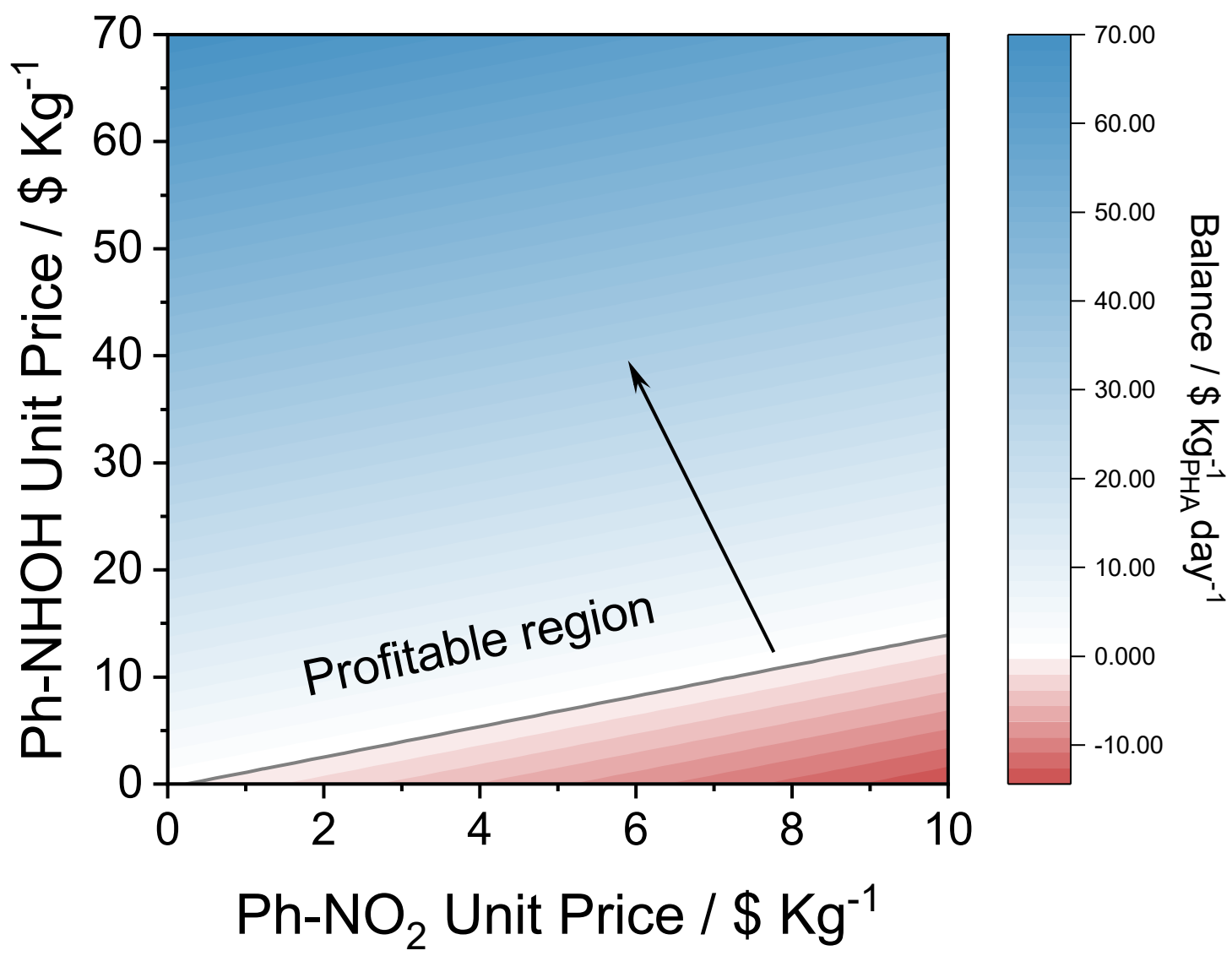

Supplementary Figure 36 The subdivided cost of the entire process for Ph-NHOH electrosynthesis from Ph-NO_2 under a current density of 1.5 A at a given electricity price of US$0.04 per kWh. Source data for Fig. S36 are provided as a Source Data file.

Supplementary Note 8 Relationships between profit, FE, and electricity price.

$$Profit = \ Ph - NHOH\ price - 1.424798 * Ph - NO_2\ price - 0.00108304\ \$\ kg^{-1} day^{-1}$$

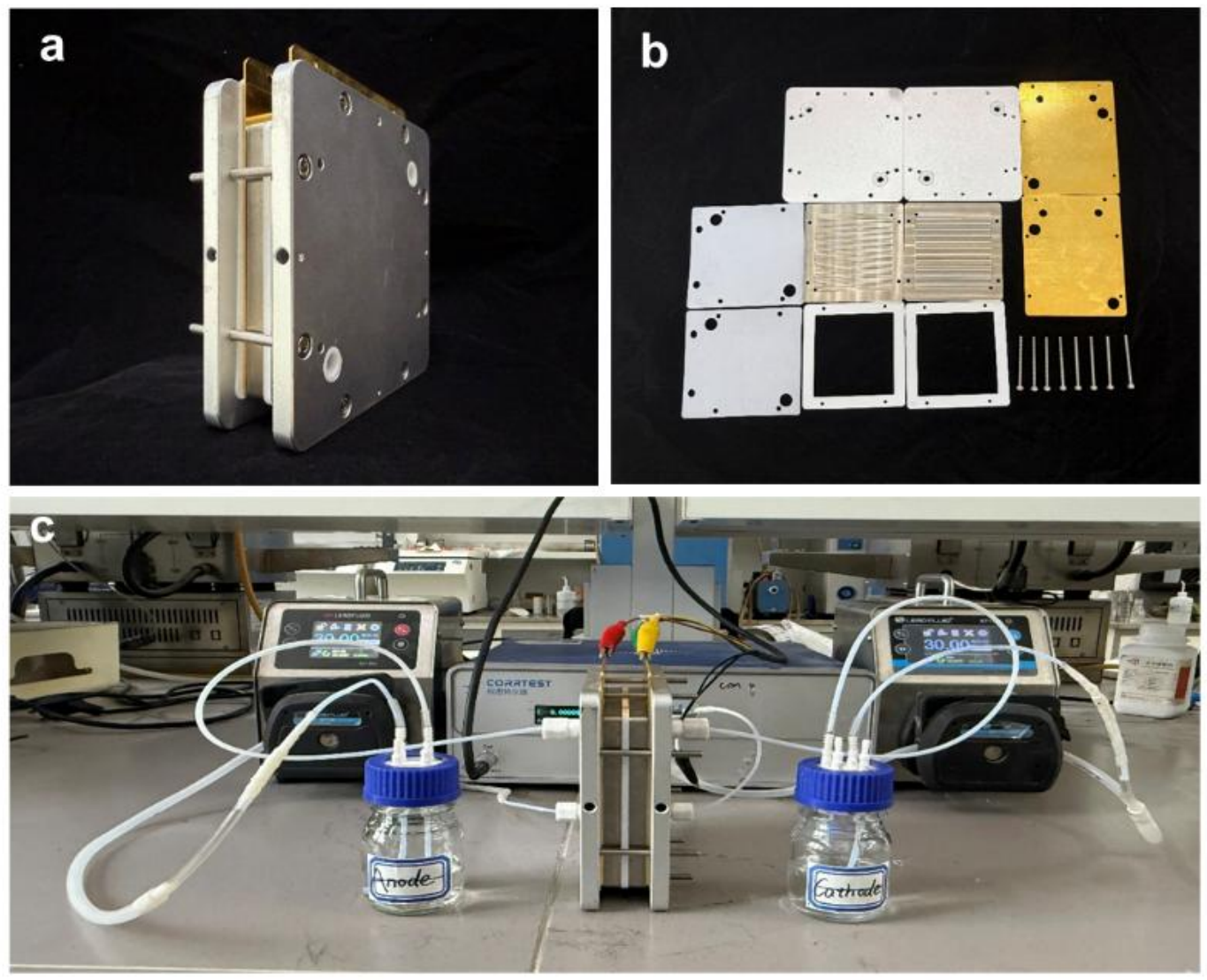

Supplementary Figure 37 Schematic diagram of the 25 cm^2 flow cell electroyter.

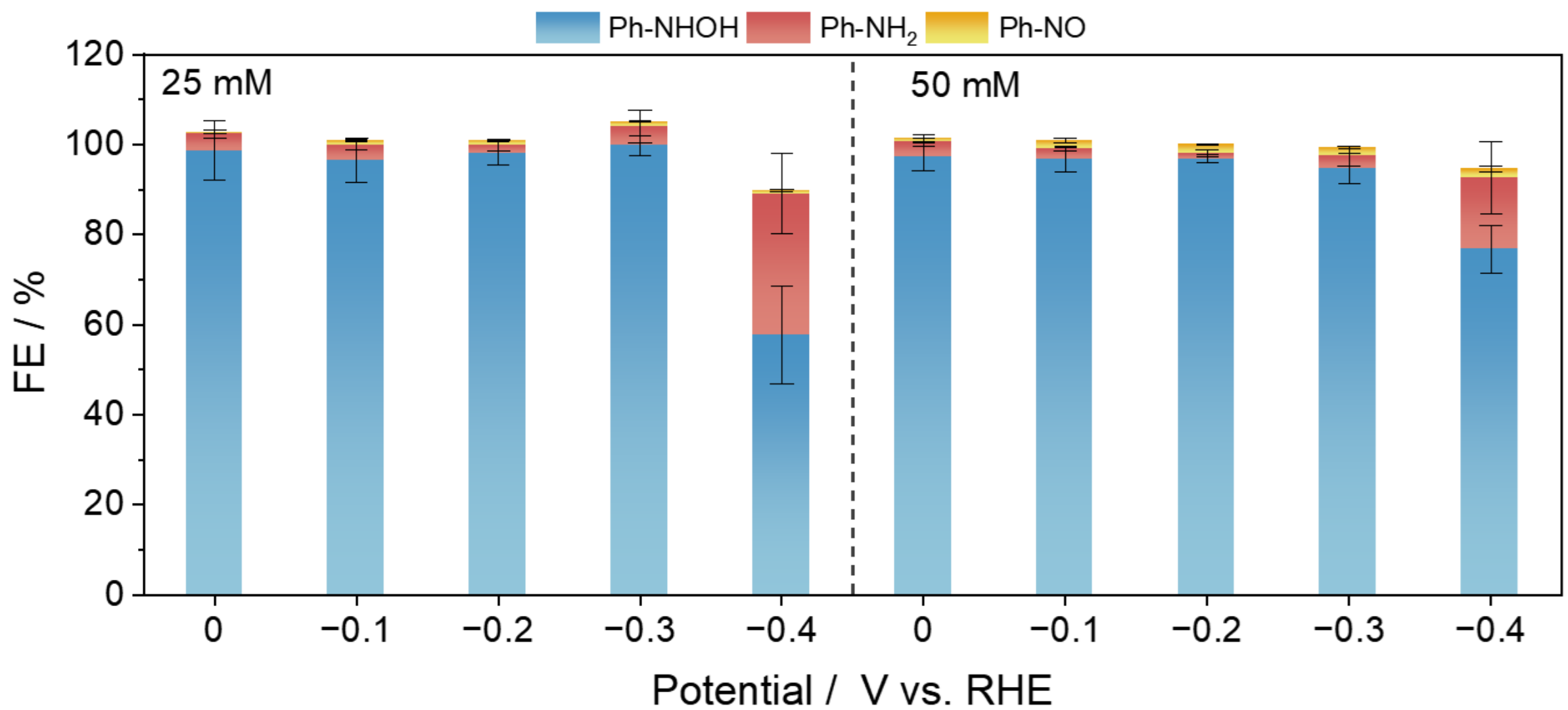

Supplementary Figure 38 FEs of CV-Ag with different Ph-NO_2 concentrations. Source data for Fig. S38 are provided as a Source Data file.

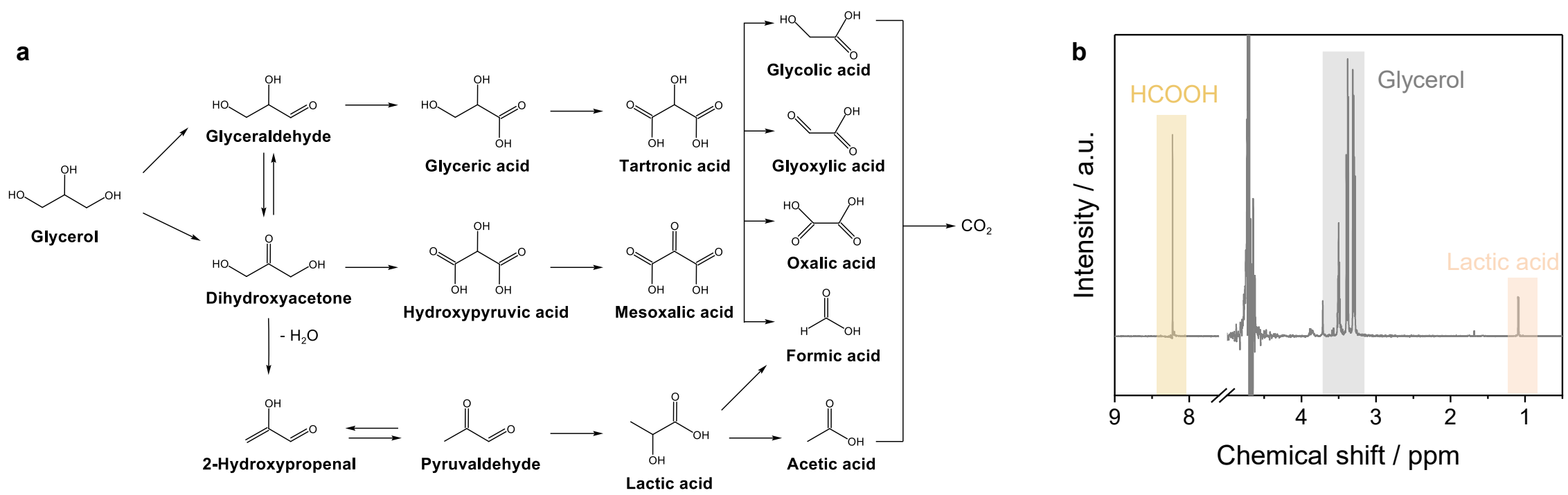

Supplementary Figure 39 a, Possible reaction pathways and main products derived from the oxidation of glycerol. **b,** ^{1}H NMR spectra of the solution for glycerol electrooxidation (GOR) at 1.5 A. Source data for Fig. S39 are provided as a Source Data file.

Supplementary Note 9 Glycerol was used as an anode additive to replace oxygen evolution reaction (OER). With a theoretical oxidation potential of 0.9 V vs. RHE, the GOR offers a significantly lower thermodynamic barrier than the OER does. Its primary oxidation product, formic acid, is widely used as a silage additive and animal biomass preservative owing to its residue-free properties during evaporation. Additionally, its acidity, aldehydic nature, and reducing properties make it valuable in various industrial applications, including as a tanning agent in leather processing, as a pH regulator in the textile sector, and as a synthetic intermediate for various pharmaceuticals and food chemicals[7]. In this work, the low theoretical oxidation potential of glycerol allows the GOR/Ph-NO_2 electroreduction pair to work at a low cell voltage, reducing the energy consumption and equipment requirements associated with high voltage and realizing chemical conversion under low energy consumption conditions.

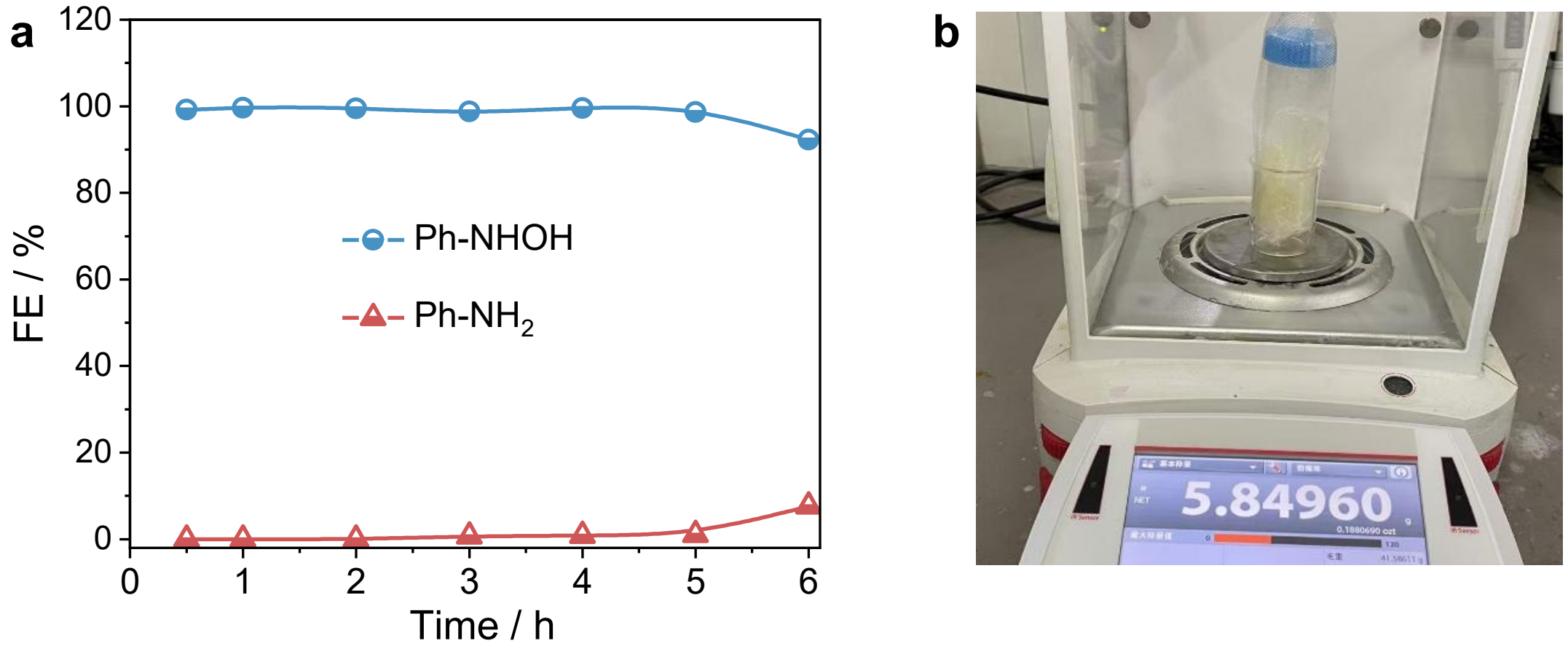

Supplementary Figure 40 a, Time-dependent voltage curve and FEs over CV-Ag at 1.5 A. **b,** Optical photograph of the Ph-NHOH product and its yield. Source data for Fig. S40 are provided as a Source Data file.

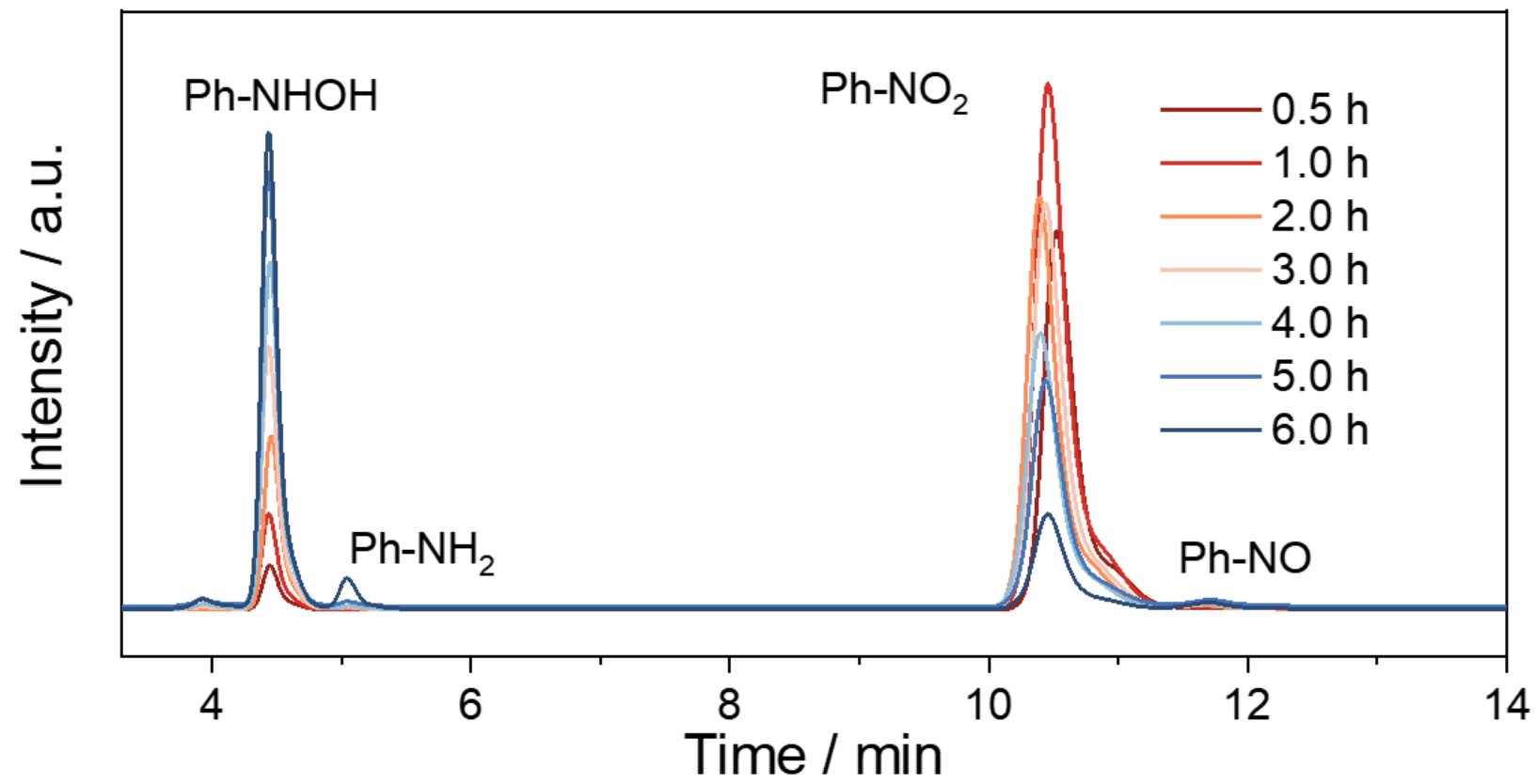

Supplementary Figure 41 HPLC chromatograms for the electrochemical reaction of Ph-NHOH obtained at 1.5 A over the CV-Ag catalyst. Source data for Fig. S41 are provided as a Source Data file.

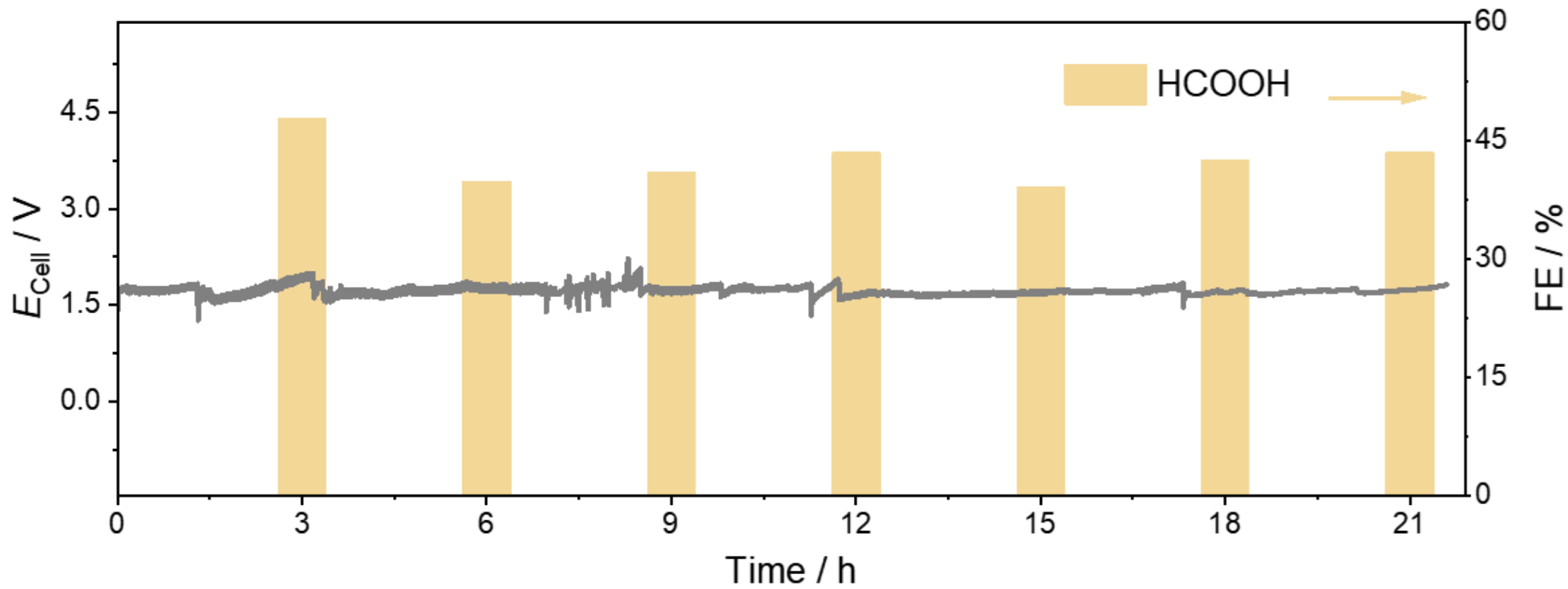

Supplementary Figure 42 Time-dependent voltage curve and HCOOH FE over Ni foam at 1.5 A. Source data for Fig. S42 are provided as a Source Data file.

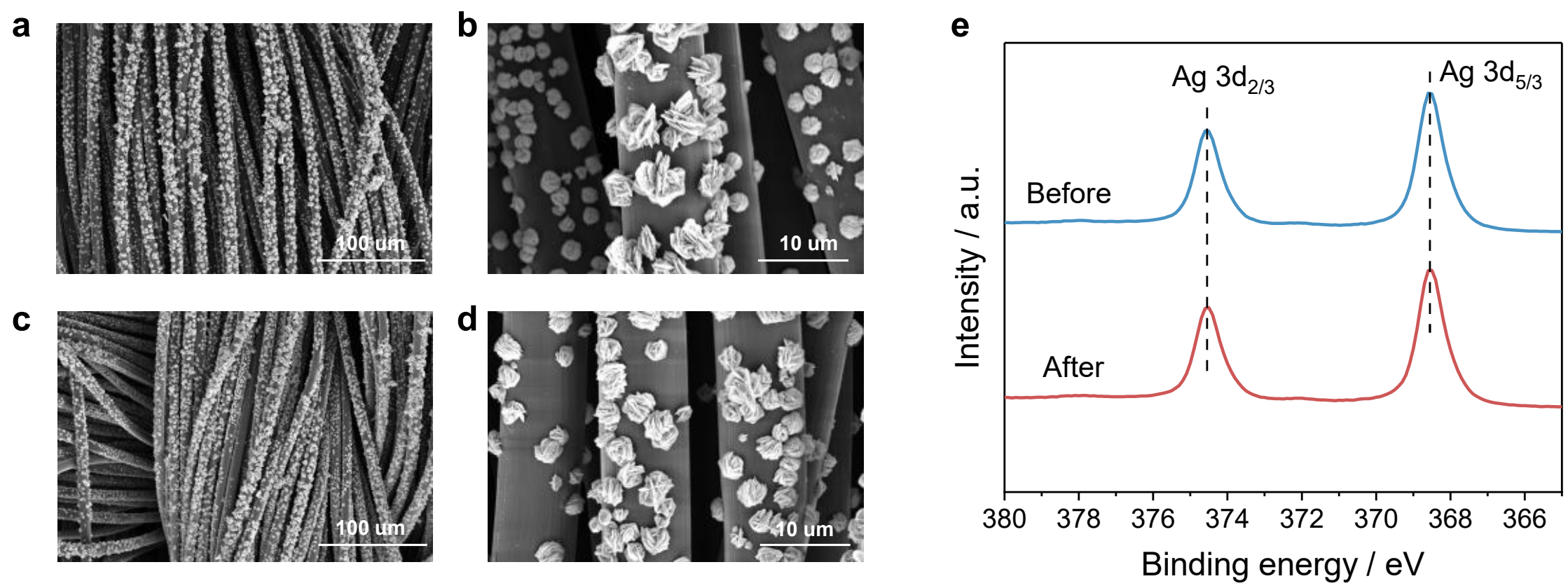

Supplementary Figure 43 a-b, SEM images of CV-Ag deposited on carbon cloth and **c-d,** SEM images of CV-Ag after long-term electrolysis. **e,** XPS spectra of CV-Ag before and after long-term electrolysis. Source data for Fig. S43 are provided as a Source Data file.

Supplementary Note 10 SEM images show that the original morphology of the catalyst was maintained after prolonged high-current electrolysis, with no obvious agglomeration, detachment, or structural collapse. XPS measurements further confirmed that the binding energy peaks of Ag did not shift compared with those of the fresh CV-Ag catalyst, indicating that the chemical valence state of Ag remained stable. These results confirm the stability of the CV-Ag catalyst during long-term operation.

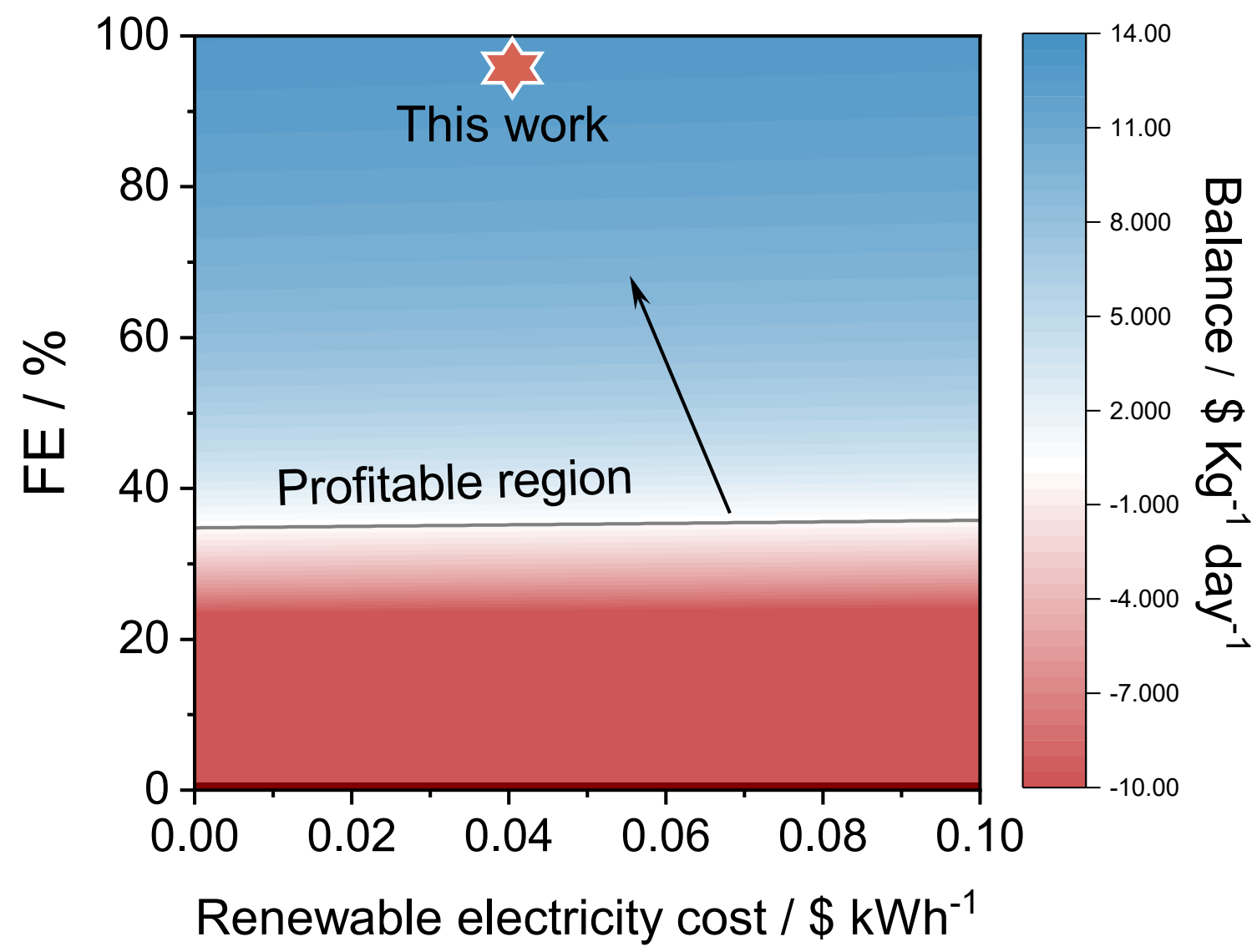

Supplementary Figure 44 Plant-gate levelized cost per tonne of Ph-NHOH from TEA at 1.5 A. Source data for Fig. S44 are provided as a Source Data file.

Supplementary Note 11 The functional relationships between profit, FE, and material price.

$$Profit = 20 - \frac{6.953071271}{FE} - 2.005344 \times \frac{electricity\ price}{FE}\ \$\ kg^{-1}day^{-1}$$

Supplementary Table 1 Gibbs free energy correction for adsorption intermediates, which were obtained with DFT through vibrational analyses via VASPKIT.

Species	***ZPE*/eV**	***T*△*S*/eV**
*PhNO$_2$H	2.95	0.35
*Ph-NO	2.58	0.33
*Ph-NHO	2.89	0.30
*Ph-NOH	2.84	0.29
*Ph-NHOH	3.23	0.30
*Ph-NH	2.79	0.28

Supplementary Table 2 The solvation energies (E_{sol}) for each adsorbate. The solvation corrections used here were explicitly calculated via $VASP_{sol}$.

Species	E_{sol}/eV
*$PhNO_2H$	–0.14
*Ph-NO	–0.13
*Ph-NHO	–0.21
*Ph-NOH	–0.62
*Ph-NHOH	–0.33
*Ph-NH	–0.09

Supplementary Table 3 EXAFS fitting parameters at the Ag K-edge.

Samples	Shell	CN[a]	R (Å)[b]	ΔE_0 (eV)[c]	σ^2(Å^2·10^{-3})[d]	R-factor (%)
**CV-Ag**	Ag−Ag	10.7±0.78	2.862±0.005	2.998±0.507	9.43±0.67	1.8
**Bulk Ag**	Ag−Ag	12	2.865±0.002	3.103±0.220	10.24±0. 30	0.25

Supplementary Note 12 [a]CN: coordination number; [b]R: bond distance; [c]ΔE_0: inner potential correction. [d]σ^2: Debye–Waller factors; R-factor: goodness of fit. S_0^2 was set as 0.929 for the Ag data, which was obtained from the experimental EXAFS fit of the Ag foil reference by fixing CN as the known crystallographic value and was fixed to all the samples. The k^2 weighting, k range of 1~12.4 Å$^{-1,}$ and R range of 1~3 Å were used for fitting.

Supplementary Table 4 Selectivity and FE of various phenylhydroxylamine derivatives

Groups at the para-position	Sele./%	FE/%
−F	93.9	87.1
−Cl	98.0	96.2
−I	97.0	87.9
−Br	98.2	95.6
$-CH_3$	91.9	81.0
−CN	98.0	96.6
−CHO	95.9	87.1
$-COOCH_3$	94.3	83.8
$-COCH_3$	96.2	88.6

Supplementary Table 5 Comparison of the performance of Ph-NO_2-to-Ph-NHOH with that of other reported methods.

Catalysts	Catalysis technique	Solvent and electrolyte	Reaction conditions	Performance	Refs.
CV-Ag	Electrocatalysis	0.5 M phosphate solution + 25 mM Ph-NO_2	0~-0.3 V vs. RHE	[a]99% [b]99%	**This Work**
		0.5 M phosphate solution + 50 mM Ph-NO_2	1.5 A	[a]99%	
EDA-Pt NWs	liquid-phase hydrogenation	MeOH	H_2/25°C	[b]98%	8
Bi(I)	Chemical reduction	1,4-dioxane	NH_3BH_3/35°C	[c]89%	9
Au@PPh_2-PIILP	Photocatalysis	H_2O	$NaBH_4$/25°C	[c]97%	10
Au@PtNi	Electrocatalysis	0.1 M Na_2SO_4 10 mg/L Ph-NO_2	0.2 V vs. RHE	[b]~70%	11
Ir@Ps	Catalytic hydrogenation	PEG-400	$N_2H_4 \cdot H_2O$/80°C	[c]87%	12
BaNTR1	Enzymatic catalysis	0.1 M PBS + glucose	30°C	[c]60%	13
SS-Pt	Catalytic hydrogenation	PEG-400	$N_2H_4 \cdot H_2O$/60°C	[c]92%	14
Ru/CNT	Catalytic hydrogenation	THF	$N_2H_4 \cdot H_2O$/25°C	[c]97%	15
Se	Catalytic hydrogenation	EtOH	Se + $N_2H_4 \cdot H_2O$/23-26°C	[c]79%	16
–	Chemical reduction	MeCN	$MeNHNH_2$/28 °C	[c]95%	17
–	Chemical reduction	C_6H_6	Sn/PhSH/Et_3N/-20°C	[c]81%	18

[a]FE, [b]Sele, [c]Yield

Supplementary Table 6 ICP–MS analysis of the Ag content in the electrolyte before and after 20 h of long-term electrolysis

Element	Before electrolysis	After electrolysis
**Ag**	0.64 ppb	0.68 ppb

1. Jouny, M., Luc, W. & Jiao, F. General techno-economic analysis of CO_2 electrolysis systems. *Ind. Eng. Chem. Res.* **57**, 2165–2177 (2018).
2. Barecka M. H., Ager J. W., Lapkin A. A. Techno-economic assessment of emerging CO_2 electrolysis technologies. *STAR protoc.*, **2**, 100889 (2021).
3. Shin, H., Hansen, K.U. & Jiao, F. Techno-economic assessment of low-temperature carbon dioxide electrolysis. *Nat. Sustain.* **4**, 911–919 (2021).
4. Li, X*., et al.* In situ SERS reveals the route regulation mechanism mediated by bimetallic alloy nanocatalysts for the catalytic hydrogenation reaction. *Chem. Sci.* **14**, 3554–3561 (2023).
5. Palacios-Ramírez, J. I. & González, F. J. Revisiting the electrochemical reduction mechanism of nitrosobenzene in acetonitrile: concomitant reactions and voltammetric simulation. *ChemElectroChem* **11**, e202300486 (2024).
6. McCrory, C. C. L., et al. Benchmarking hydrogen evolving reaction and oxygen evolving reaction electrocatalysts for solar water splitting devices. *J. Am. Chem. Soc.* **137**, 4347−4357 (2015).
7. Hietala, J. et al. in *Ullmann's Encyclopedia of Industrial Chemistry* 1–22 (Wiley-VCH Verlag GmbH & Co. KGaA, Weinheim, Germany, 2016).
8. Chen, G., et al. Interfacial electronic effects control the reaction selectivity of platinum catalysts. *Nat. Mater.* **15**, 564–569 (2016).
9. Wang, F., et al. Bi(I)-Catalyzed transfer-hydrogenation with ammonia-borane. *J. Am. Chem. Soc.* **141**, 4235–4240 (2019).
10. Doherty, S., et al. Highly Selective and Solvent-Dependent Reduction of Nitrobenzene to *N*-Phenylhydroxylamine, Azoxybenzene, and aniline catalyzed by phosphino-modified polymer immobilized ionic liquid-stabilized AuNPs. *ACS Catal.* **9**, 4777–4791 (2019).
11. Ma, J., et al. Role of Ni in PtNi alloy for modulating the proton–electron transfer of electrocatalytic hydrogenation revealed by the in situ Raman–rotating disk electrode method. *ACS Catal.* **12**, 14062–14071 (2022).
12. Bhattacherjee, D., et al. Polystyrene stabilized iridium nanoparticles catalyzed chemo- and regio-selective semi-hydrogenation of nitroarenes to *N*-arylhydroxylamines. *Mol. Catal.* **514**, 111836 (2021).

13. Nguyen-Tran, H.-H., Zheng, G.-W., Qin, X.-H. & Xu, J.-H. Highly selective and controllable synthesis of arylhydroxylamines by the reduction of nitroarenes with an electron-withdrawing group using a new nitroreductase BaNTR1. *Chem. Commun.* **50**, 2861–2864 (2014).
14. Shil, A. K. & Das, P. Solid supported platinum(0) nanoparticles catalyzed chemo-selective reduction of nitroarenes to *N*-arylhydroxylamines. *Green Chem.* **15** (12), 3421–3428 (2013).
15. Jawale, D. V., et al. Selective conversion of nitroarenes using a carbon nanotube–ruthenium nanohybrid. *Chem. Commun.* **51**, 1739–1742 (2015).
16. Yanada, K., Yamaguchi, H., Meguri, H. & Uchida, S. Selenium-catalysed reduction of aromatic nitro compounds to *N*-arylhydroxylamines. *Chem. Commun.* **22**, 1655–1656 (1986).
17. Kallitsakis, M. G., Ioannoun, D. I., Terzidis, M. A., Kostakis, G. E. & Lykakis, I. N. Selective photoinduced reduction of nitroarenes to *N*-arylhydroxylamines. *Org. Lett.* **22**, 4339–4343 (2020).
18. Bartra, M., Romea, P., Urpi, F. & Villarrasa, J. A fast procedure for the reduction of azides and nitro compounds based on the reducing ability of $Sn(SR)_3$-species. *Tetrahedron.* **46**, 587–594 (1990).
