# Supplementary material for: Metal-mediated weak adsorption enables the selective electrosynthesis of N-phenylhydroxylamine from nitrobenzene hydrogenation in water
Source: Nat Commun. 2026 Jul 30;17:9221. doi: 10.1038/s41467-026-76066-6 (PMC13522461; doi:10.1038/s41467-026-76066-6)
Supplement: Supplementary file 2 [file 41467_2026_76066_MOESM2_ESM.pdf]

Description of Additional Supplementary Files

File Name: Supplementary Data 1

Description: The adsorption configurations of the reaction intermediates involved in the nitrobenzene reduction reaction on different metals.
