# Supplementary material for: Metal-mediated weak adsorption enables the selective electrosynthesis of N-phenylhydroxylamine from nitrobenzene hydrogenation in water
Source: Nat Commun. 2026 Jul 30;17:9221. doi: 10.1038/s41467-026-76066-6 (PMC13522461; doi:10.1038/s41467-026-76066-6)
Supplement: Supplementary file 3 [file 41467_2026_76066_MOESM4_ESM.pdf]

nature portfolio

# Peer Review File

# Metal-mediated weak adsorption enables the selective electrosynthesis of N-phenylhydroxylamine from nitrobenzene hydrogenation in water

Corresponding Author: Professor Bin Zhang

**This file contains all reviewer reports in order by version, followed by all author rebuttals in order by version.**

Version 0:

Reviewer comments:

Reviewer #1

(Remarks to the Author)
This manuscript reports the efficient electrocatalytic synthesis of N-phenylhydroxylamine (Ph-NHOH) from nitrobenzene (Ph-$NO_2$) and demonstrates that the weak adsorption of the Ph-NHOH intermediate on Ag metal is crucial for preventing overhydrogenation to Ph-$NH_2$, thereby enabling high selectivity. Through a combination of electrochemical in situ FTIR spectroscopy and density functional theory (DFT) calculations, the authors provide valuable mechanistic insights. Additionally, gram-scale Ph-NHOH electrosynthesis was achieved in a flow MEA electrolyser. Therefore, I recommend this manuscript for acceptance in Nature Communications. The minor points listed below are suggested to further enhance clarity and impact.

1. The Ph-NHOH selectivity of the bulk Ag electrode is 98.0%, which is virtually identical to that of the optimized CV-Ag. This raises the following question: what is the fundamental improvement offered by nanostructured CV-Ag? The data suggest that it does not improve intrinsic selectivity.
2. In the experiments conducted at different pH levels, only the conditions at pH 12.7 and 5.8 were mentioned. How about the performance in more acidic conditions?
3. The impressive increase to 99.6% FE in the MEA system is a key finding, yet its origin remains insufficiently explained. Does the different reactor configurations or the high current density intrinsically favor Ph-NHOH formation? A mechanistic discussion is therefore needed.
4. The OER was replaced by the AOR. Therefore, what are the corresponding results or performance of the AOR?
5. A few minor errors should be checked and corrected: For instance, in Figure 3, it is necessary to clarify the label on the y-axis in the IR spectra whether it represents absorbance or transmittance. Additionally, in Figure 4b, the horizontal axis label should be R, not k.

Reviewer #2

(Remarks to the Author)
In this manuscript, Zhang and coworkers constructed a Ag-based catalyst for the selective electrosynthesis of N-phenylhydroxylamine from nitrobenzene, achieving impressive selectivity and ampere-level current density. Detail mechanistic studies have been carried out, revealing the weak adsorption of Ph-NHOH on Ag is key to catalytic efficiency. Overall, this topic is industrially important, and this work is systematically investigated and well organized. However, several critical issues remain, and should be addressed before publication:
1. The authors chose *Ph-NO adsorption energy ($G^*_{ad}$(Ph-NO)) as the descriptor for constructing the activity volcano plot. Please provide a more detailed justification for this choice. Specifically, why is *Ph-NO the most appropriate descriptor among all possible intermediates? Furthermore, given that the experimental results ultimately highlight the critical role of weak *Ph-NHOH adsorption in determining selectivity, how do the authors reconcile the use of *Ph-NO as the initial descriptor with the final conclusion centered on *Ph-NHOH? Is there a scaling relationship that links the adsorption energies of *Ph-NO and *Ph-NHOH across the different metal surfaces?
2. In the in situ ATR-FTIR spectroscopy results, the authors assign the peak near 1500 cm-1 to the C–H bending vibration of Ph-NH2 and use this as evidence for its formation, but the reviewer does not understand the reasoning behind this assignment. Furthermore, this peak is very weak in intensity and is close to the noise level in some spectra.

3. The authors couple cathodic Ph-NO2 reduction with anodic ascorbic acid oxidation (AOR) to significantly reduce the cell voltage. While this is an elegant strategy for energy savings, a critical practical concern needs to be addressed: what is the economic viability of using ascorbic acid as a sacrificial agent on a large scale? Ascorbic acid is relatively expensive, and its oxidation product (dehydroascorbic acid) would need to be separated and potentially discarded. Have the authors considered the cost of the ascorbic acid consumed in the process? A more comprehensive cost-benefit analysis that includes the expense of the anode reactant, rather than just the electricity savings, would be necessary to truly assess the industrial potential.
4. The authors attribute the lowest Ph-NHOH selectivity on the Pd electrode (51.7%) primarily to the strong adsorption of Ph-NHOH on its surface, which promotes overhydrogenation to Ph-NH2. However, Pd is also well-known for its excellent hydrogen evolution reaction (HER) activity and ability to provide high coverage of adsorbed hydrogen (*H). Given this, how can the authors definitively rule out the possibility that the low selectivity on Pd is, at least in part, due to a higher surface coverage of *H, which could accelerate the hydrogenation steps? Could the authors decouple the *H coverage effect from the Ph-NHOH adsorption strength, perhaps by comparing the HER activity or performing *H titration experiments on the different metal electrodes?
5. The electrochemical measurements were primarily conducted in a 0.5 M PBS buffer at pH=5.8. Given the proton-coupled electron transfer (PCET) nature of the reaction, a systematic investigation of the pH effect would strengthen the mechanistic understanding. The authors are requested to provide data on the Ph-NO2 conversion, Ph-NHOH selectivity, and FE for the CV-Ag catalyst across a wider pH range (e.g., from strongly acidic pH=1 to near-neutral pH=7). This is particularly important to determine if the chemical stability of Ph-NHOH becomes a limiting factor under more acidic conditions, which would be crucial for process design.
6. The enhanced performance of the CV-Ag catalyst is primarily attributed to its enlarged electrochemical surface area (ECSA). While ECSA is certainly a factor, the unique surface structure of electrodeposited nanoparticles, such as grain boundaries, defects, and undercoordinated sites, can also impart distinct intrinsic activity. How do the authors exclude the possibility that the improved performance of CV-Ag, compared to bulk Ag, stems from these unique surface atomic configurations rather than just an increase in the number of active sites? A normalization of the activity by ECSA (i.e., specific activity) is already provided, but a more direct characterization of the surface defects (e.g., using lead underpotential deposition or high-resolution TEM with geometric phase analysis) and correlating this with the weak adsorption property would be insightful.
7. The technoeconomic analysis (TEA) presented in the manuscript, estimating a profit of $520 per tonne of Ph-NHOH, is a strong point. However, this analysis appears to be based primarily on electricity and feedstock costs. It seems to omit the significant costs associated with downstream processing, specifically the product separation and purification steps. The Methods section describes extraction with ethyl acetate and recrystallization using petroleum ether and dichloromethane. These unit operations involve substantial solvent costs, energy for distillation/recovery, and waste management. The authors are requested to provide a revised TEA that explicitly includes the costs of these downstream separation processes to present a more realistic "plant-gate" cost and a fairer comparison with existing chemical routes.
8. The substrate scope demonstrates excellent compatibility with halogens, methyl, and cyano groups. To truly define the application boundaries of this Ag-catalyzed system, it is essential to test its tolerance towards more sensitive functional groups. The authors are encouraged to expand the substrate scope to include nitroaromatics bearing easily reducible groups, such as esters (e.g., methyl 4-nitrobenzoate), aldehydes (e.g., 4-nitrobenzaldehyde), or ketones (e.g., 4-nitroacetophenone). These experiments would clarify whether the high selectivity for the hydroxylamine can be maintained when competing, more electrophilic sites are present in the molecule, which is a common challenge in organic synthesis.
9. The long-term stability test over 20 hours at 1.5 A is impressive and crucial for practical application. However, several operational details are missing. Firstly, how was the nitrobenzene reactant replenished during this 20-hour period to maintain a constant concentration? Was it a batch process with periodic replacement of the catholyte, or was a continuous flow system used? Secondly, and more importantly, a post-mortem characterization of the CV-Ag catalyst after this prolonged electrolysis is necessary. The authors should provide SEM images to check for morphological changes (e.g., nanoparticle agglomeration or detachment), XPS analysis to examine any changes in the chemical state or surface composition (e.g., oxidation or poisoning), and ICP-MS analysis of the electrolyte to quantify any potential leaching of Ag. This data is vital to substantiate the claim of excellent durability.

Reviewer #3

(Remarks to the Author)
Lu et al. demonstated a selective electrochemical route for converting Ph-NO2 to high-value Ph-NHOH. This study highlights the pivotal role of Ag in enabling weak adsorption of Ph-NHOH, which is supported by the results of DFT and in situ FTIR. Then, the authors successfully achieved highly selective electrosynthesis of Ph-NHOH, overcoming the thermodynamic unfavorability of its formation. This innovative and impactful study advances our understanding of selectivity control in Ph-NHOH electrosynthesis. Accordingly, this work is suitable for publication in Nature Communications once the following issues have been addressed.
1. When the adsorption energies of Ph-NO, Ph-NHOH, and other intermediates are calculated, multiple adsorption modes are possible, which may influence the selectivity among Ph-NHOH, Ph-NH2, and azoxy products. Which configuration is adopted for each intermediate in the DFT discussion? Does the benzene ring participate in flat-lying adsorption or -NO2-centered vertical adsorption?
2. In the context of this multistep reaction system, the choice of Ph-NO adsorption energy as a universal descriptor requires further justification, particularly given that the adsorption/desorption behavior of Ph-NHOH governs selectivity.
3. For the mechanistic analysis, all figures presented in the Supporting Information require detailed captions, including experimental conditions, to make them understandable to a general audience.
4. In Figure 22, the LSV curve shows obvious current fluctuation after adding Ph-NO2, and can it be avoided?

5. For the initial experiments that established Ag as the volcano-apex metal, what were the detailed electronic structure and surface crystallography of the other metals tested?
6. To better illustrate the superiority of this method, the authors could focus the functional group compatibility discussion on several particularly challenging cases.

Reviewer #4

(Remarks to the Author)

Version 1:

Reviewer comments:

Reviewer #1

(Remarks to the Author)
The authors have greatly revised the manuscript according to the comments from the Reviewers. It can be accepted for publication in its current version.

Reviewer #2

(Remarks to the Author)
The author has properly addressed the comments. This reviewer recommends the publication of revised manuscript.

Reviewer #3

(Remarks to the Author)
The reviewer is satisfied with the revisions the authors have made. I recommend its publication in Nature Communications without changes.

Reviewer #4

(Remarks to the Author)
The electrochemical reduction of nitrobenzene to N-phenylhydroxylamine is reported on different electrode types. The work is significant and the revision has improved the quality. Publication is now recommended.

**Open Access** This Peer Review File is licensed under a Creative Commons Attribution 4.0 International License, which permits use, sharing, adaptation, distribution and reproduction in any medium or format, as long as you give appropriate credit to the original author(s) and the source, provide a link to the Creative Commons license, and indicate if changes were made.
In cases where reviewers are anonymous, credit should be given to 'Anonymous Referee' and the source.

The images or other third party material in this Peer Review File are included in the article's Creative Commons license, unless indicated otherwise in a credit line to the material. If material is not included in the article's Creative Commons license and your intended use is not permitted by statutory regulation or exceeds the permitted use, you will need to obtain permission directly from the copyright holder.
To view a copy of this license, visit https://creativecommons.org/licenses/by/4.0/

# A point-by-point response to the reviewers

## To Reviewer 1:

### Reviewer Letter:

This manuscript reports the efficient electrocatalytic synthesis of *N*-phenylhydroxylamine (Ph-NHOH) from nitrobenzene (Ph-$NO_2$) and demonstrates that the weak adsorption of the Ph-NHOH intermediate on Ag metal is crucial for preventing overhydrogenation to Ph-$NH_2$, thereby enabling high selectivity. Through a combination of electrochemical in situ FTIR spectroscopy and density functional theory (DFT) calculations, the authors provide valuable mechanistic insights. Additionally, gram-scale Ph-NHOH electrosynthesis was achieved in a flow MEA electrolyser. Therefore, I recommend this manuscript for acceptance in Nature Communications. The minor points listed below are suggested to further enhance clarity and impact.

**Answer:** We are very grateful to the reviewers for their positive comments on our work. In accordance with the reviewers' comments, we have addressed all the issues. To save reviewers valuable time, key revisions are displayed on a yellow background in the revised manuscript and supporting information (SI).

**Comment 1.** The Ph-NHOH selectivity of the bulk Ag electrode is 98.0%, which is virtually identical to that of the optimized CV-Ag. This raises the following question: what is the fundamental improvement offered by nanostructured CV-Ag? The data suggest that it does not improve intrinsic selectivity.

**Answer:** We acknowledge the reviewer's insightful comments, which allow us to clarify the fundamental improvement offered by the CV-Ag catalyst. We fully agree that the high selectivity of Ag is already optimal. The key improvement in CV-Ag is not only enhancing selectivity but also significantly enhancing catalytic efficiency and practical performance while fully preserving this high selectivity. Although the selectivity of Ph-NHOH is similar, compared with the bulk Ag electrode, CV-Ag achieves a higher Ph-NHOH yield rate at the same overpotential. This directly proves the significant improvement in its catalytic efficiency due to nanostructure optimization. In addition, the larger ECSA of CV-Ag ensures that the yield of Ph-NHOH per unit time and per unit area on CV-Ag is

greater than that on the Ag electrode. This means a smaller reactor volume and higher production efficiency at the industrial scale, which is a fundamental improvement.

**Comment 2.** In the experiments conducted at different pH levels, only the conditions at pH 12.7 and 5.8 were mentioned. How about the performance in more acidic conditions?

**Answer:** Thank you for your careful review. As suggested, we systematically supplemented the catalytic performance of CV-Ag in strongly acidic electrolytes (pH 1.0–5.0). The results show that high Ph-NHOH selectivity and FE are well maintained under acidic conditions (**Fig. R1**). Given that the Ph-NHOH selectivity at pH 5.8 is comparable to that in strongly acidic electrolytes, we chose weakly acidic conditions (pH 5.8) for subsequent reaction studies, considering factors such as reaction mildness, metal catalyst stability, and equipment corrosion.

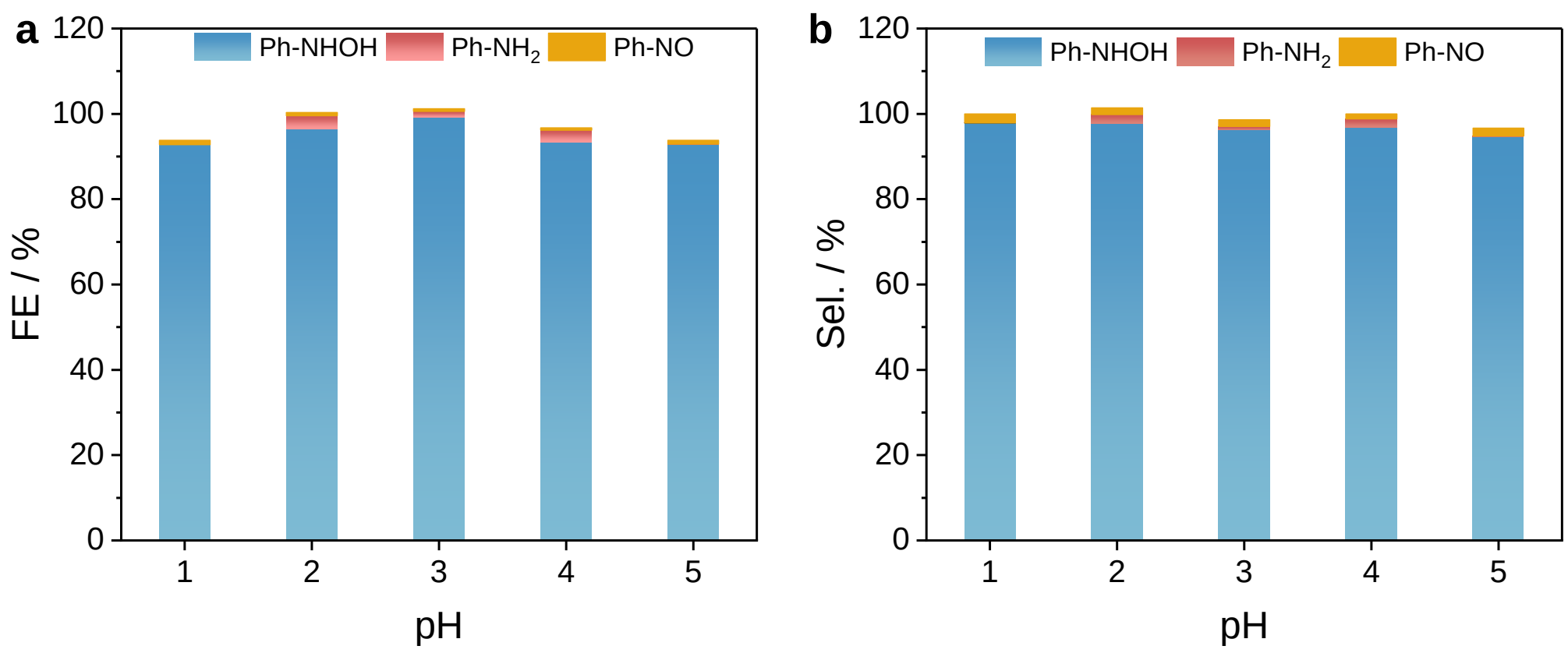


**Fig. R1** Product distribution over the CV-Ag electrodes in acidic electrolytes (pH 1.0–5.0).

**Comment 3.** The impressive increase to 99.6% FE in the MEA system is a key finding, yet its origin remains insufficiently explained. Does the different reactor configurations or the high current density intrinsically favor Ph-NHOH formation? A mechanistic discussion is therefore needed.

**Answer:** We acknowledge the reviewer's kind comments. The improved mass transfer (reducing the residence time of intermediate products) may indeed be beneficial for selectivity. In the H-type cell, the generated Ph-NHOH has a greater chance of diffusing back to the electrode surface and being further reduced, whereas in the MEA reactor, the product is quickly removed from the reaction interface.

To prove this point, we compared the reaction processes in different reactors (**Fig. R2**). In the H-type cell, we observed a high FE for Ph-NHOH generation in the initial reaction stage. As the reaction progressed, the FE rapidly decreased. In the MEA reactor, the Ph-NHOH FE remained at a high level of 99.6% in the initial stage. After 6 h of continuous electrolysis, a similar trend in the FE was observed. This trend suggests that the change in the reaction microenvironment may influence the Ph-NHOH FE. Compared with the H-type cell, better FE retention in the later reaction stage was observed in the MEA reactor. This is attributed to the superior mass transport efficiency in the MEA reactor. The enhanced convective flow in the MEA reduces the residence time of the Ph-NHOH intermediate, which kinetically suppresses its further reduction to Ph-$NH_2$. This may be the main reason for the difference in selectivity between the two reactors. This phenomenon has been commonly reported (***Energy Environ. Sci.*** 2022, 15, 3603; ***Nat. Commun.*** 2026, 17, 124; ***Angew. Chem. Int. Ed.*** 2025, 64, e202506845).

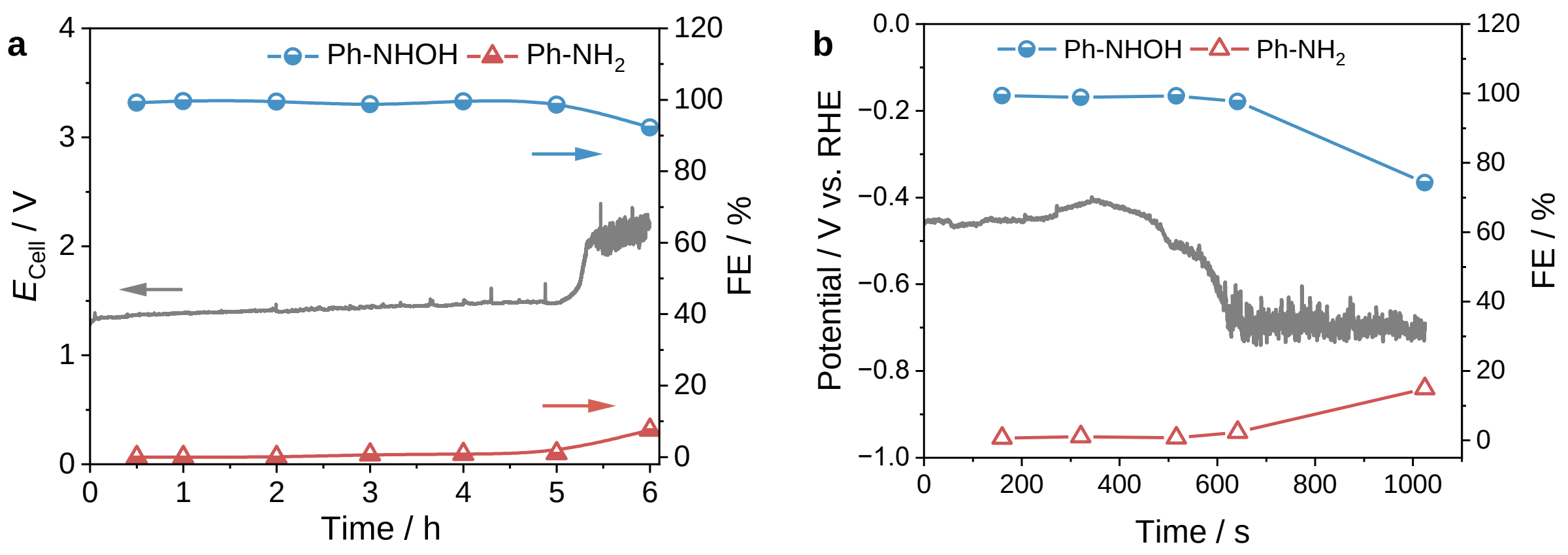


**Fig. R2** Time-dependent voltage curve and FEs over CV-Ag (a) in the MEA system and (b) in the H-type cell.

**Comment 4.** The OER was replaced by the AOR. Therefore, what are the corresponding results or performance of the AOR?

**Answer:** We appreciate your reminder. As suggested, we have supplemented the performance results of the ascorbic acid oxidation reaction (AOR). $^1$H NMR spectra of the anodic reaction under different currents confirm the generation of the target dehydroascorbic acid (DHA) product (**Fig. R3**). Using an internal standard, the DHA FEs under different currents were calculated to reach approximately 99%.

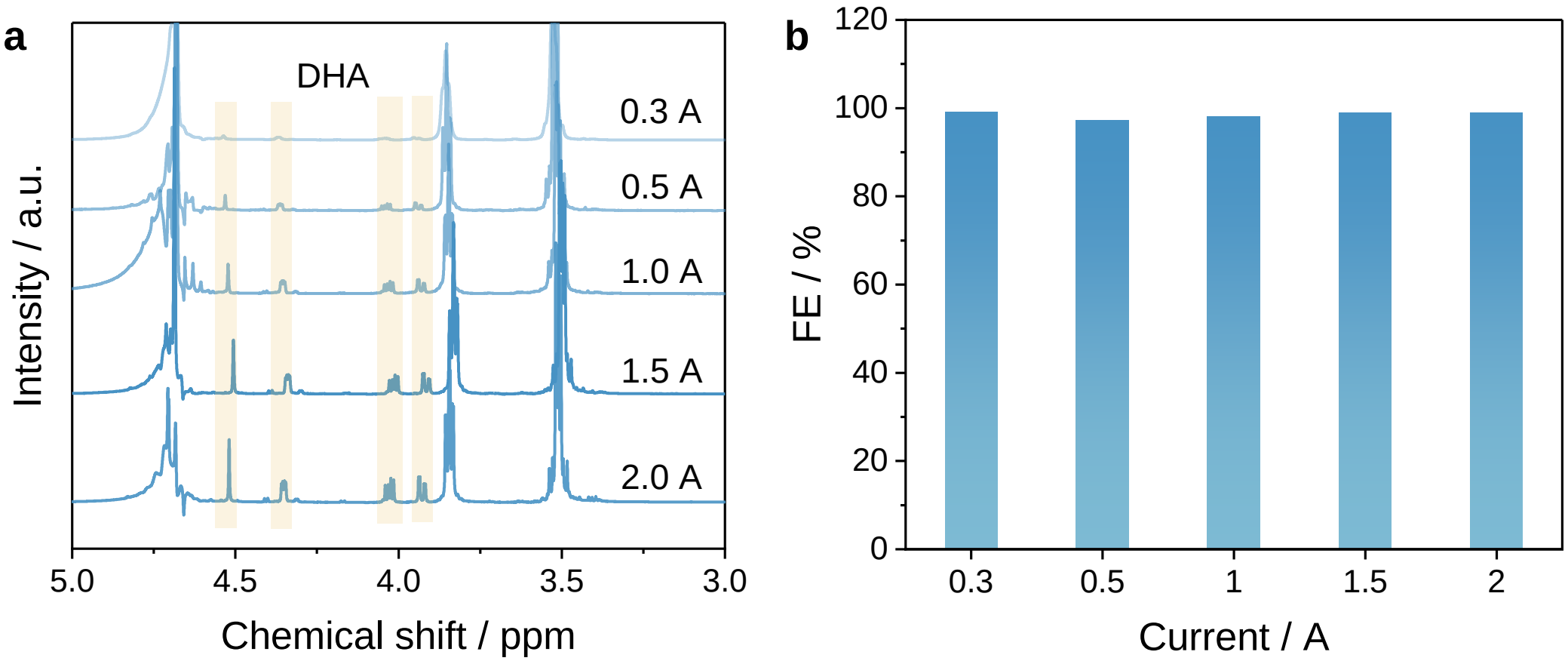


**Fig. R3** (a) $^1$H NMR spectra of DHA from the AOR. (b) DHA FE at different currents.

**Comment 5.** A few minor errors should be checked and corrected: For instance, in Figure 3, it is necessary to clarify the label on the y-axis in the IR spectra whether it represents absorbance or transmittance. Additionally, in Figure 4b, the horizontal axis label should be R, not k.

**Answer:** We thank the reviewer for pointing out these errors. We have added a clear explanation in the figure caption to explicitly indicate that the y-axis represents transmittance (not absorbance). In addition, the x-axis label in Figure 4b has been corrected to "R".

## To Reviewer 2:

## Reviewer Letter:

In this manuscript, Zhang and coworkers constructed a Ag-based catalyst for the selective electrosynthesis of N-phenylhydroxylamine from nitrobenzene, achieving impressive selectivity and ampere-level current density. Detail mechanistic studies have been carried out, revealing the weak adsorption of Ph-NHOH on Ag is key to catalytic efficiency. Overall, this topic is industrially important, and this work is systematically investigated and well organized. However, several critical issues remain, and should be addressed before publication:

**Answer:** We sincerely thank the reviewer for the positive comments. Our point-by-point responses to the concerns raised are as follows. To save the reviewer's valuable time, all key revisions have been highlighted in yellow in both the revised manuscript and the Supplementary Information (SI).

**Comment 1.** The authors chose *Ph-NO adsorption energy ($G^*_{ad}$ (Ph-NO)) as the descriptor for constructing the activity volcano plot. Please provide a more detailed justification for this choice. Specifically, why is *Ph-NO the most appropriate descriptor among all possible intermediates? Furthermore, given that the experimental results ultimately highlight the critical role of weak *Ph-NHOH adsorption in determining selectivity, how do the authors reconcile the use of *Ph-NO as the initial descriptor with the final conclusion centered on *Ph-NHOH? Is there a scaling relationship that links the adsorption energies of *Ph-NO and *Ph-NHOH across the different metal surfaces?

**Answer:** Thank you very much for the comments.

Regarding the first question, we selected the adsorption energy of *Ph-NO ($G_{ad}$(*Ph-NO)) as the descriptor to evaluate the activity of both the main and side reactions on the basis of the following two considerations:

i). Scaling relationship: Evaluating reaction activity for multistep reactions is inherently complex. Fortunately, the d-band center theory and volcano plot proposed by Nørskov and coworkers (***Nature*** 1995, 376, 238; ***Nat. Catal.*** 2023, 6, 460−461; ***J. Phys. Chem. B*** 2004, 108, 17886−17892) have provided a feasible solution. This theory leverages the linear scaling relationship (LSR) between the adsorption energies of any two intermediates, enabling the adsorption energy of any intermediate to be expressed as a linear function of that of a single intermediate (***Nat. Rev. Mater.*** 2019, 4, 792−804; ***Phys. Rev. Lett.*** 2012, 108, 116103; ***Phys. Chem. Chem. Phys.*** 2011, 13, 20760−20765;

***Angew. Chem. Int. Ed.*** 2008, 47, 4683−4686). Mathematically, the representation of all intermediates via a single descriptor is only valid when such a linear scaling relationship holds. Once the fitted linear scaling relationship is statistically significant (e.g., $R^2 > 0.8$), the selected intermediate can serve as a reliable descriptor.

ii). Chemical rationality: While the mathematical validity of the descriptor is confirmed by the linear scaling relationship, its chemical relevance must also be justified. *Ph-NO is an indispensable intermediate in the entire reaction pathway, making it applicable as a universal descriptor for all catalysts under investigation. Such a strategy is common in previous works (***Science*** 2008, 320, 1320--1322; ***Nature*** 2020, 581, 178--184; ***Nat. Commun.*** 2017, 8, 15438; ***J. Phys. Chem. Lett.*** 2012, 3, 251−258; ***Phys. Chem. Chem. Phys.*** 2014, 16, 4720−4727).

Regarding the second question, the use of *Ph-NO as a descriptor and our conclusion that *Ph-NHOH desorption dominates catalytic selectivity are not contradictory. The detailed reasons are as follows:

*Ph-NHOH is the key selectivity-determining intermediate; its desorption leads to the formation of Ph-NHOH, while its overhydrogenation results in Ph-$NH_2$. However, for certain catalysts, *Ph-NHOH cannot exist stably (e.g., it dissociates into *Ph-NH and *OH), which hinders accurate quantification of their activity. Fortunately, the volcano plots constructed using *Ph-NO as the descriptor reveal distinct shapes for the Ph-NHOH and Ph-$NH_2$ activity volcanoes. In the strong adsorption region (left side of the volcano), overhydrogenation of *Ph-NHOH is prone to occur, favouring Ph-$NH_2$ formation. In contrast, in the weak adsorption region (right side of the volcano), Ph-NHOH is thermodynamically dominant. This is because although the potential-limiting step (PLS) is the same for both Ph-NHOH and Ph-$NH_2$ formation (*Ph-$NO_2$ + $H^+$ + $e^-$ → *Ph-$NO_2H$), the sublimiting step for Ph-NHOH formation (*Ph-NHO + $H^+$ + $e^-$ → Ph-NHOH) is more easily achieved than that for Ph-$NH_2$ formation (*Ph-NHO + $H^+$ + $e^-$ → *Ph-NHOH). Thus, Ph-NHOH is more predominant in the weak adsorption region of the volcano plot. This is consistent with our in situ spectroscopy results, which demonstrate the weak adsorption intensity of *Ph-NHOH on Ag, a catalyst located on the right side of the *Ph-NO-based volcano plot.

In summary, *Ph-NO serves as a screening descriptor that identifies Ag as the optimal metal catalyst, while *Ph-NHOH desorption corresponds to the key step governing the high selectivity of Ag.

Regarding the third question, a distinct linear scaling relationship exists between the adsorption energies of *Ph-NO and *Ph-NHOH (Fig. R4), which is expressed as follows:

$$G_{ad}(*PhNHOH) = 0.7642 \times G_{ad}(*PhNO) - 0.6204$$

The correlation coefficient $R^2$ is 0.98, indicating a highly reliable linear relationship. This means that for a given catalyst, a weak adsorption energy of one intermediate (e.g., *Ph-NO) is accompanied by a similarly weak adsorption trend for other intermediates (e.g., *Ph-NHOH). Therefore, catalysts with weak *Ph-NHOH adsorption (which exhibit excellent Ph-NHOH selectivity) are distributed on the left side of the *Ph-NO-based volcano plot, further validating the rationality of using *Ph-NO as the initial activity descriptor.

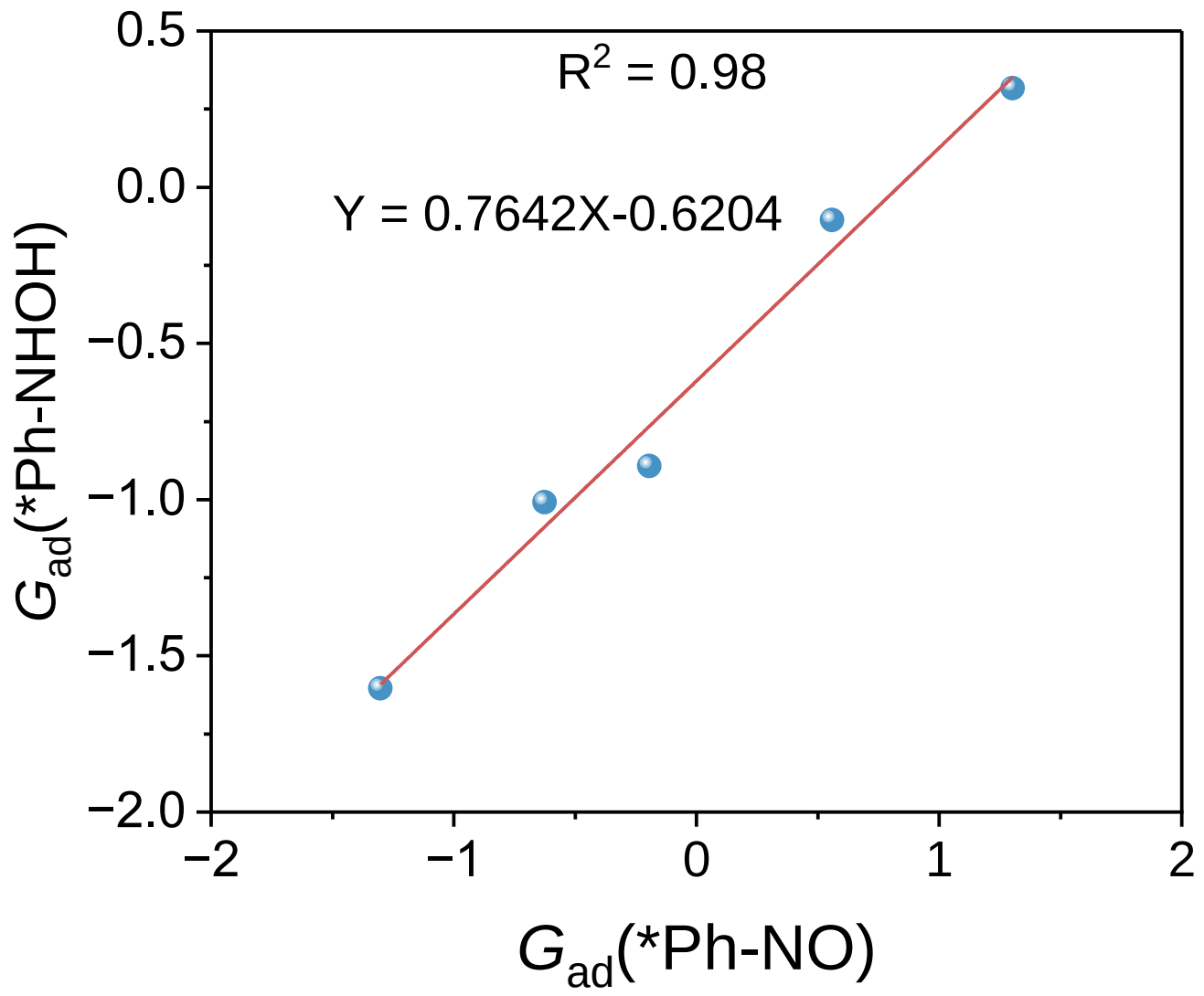


**Fig. R4** Scaling relationships of the $G_{ad}$(*Ph-NHOH) vs. $G_{ad}$(*Ph-NO).

**Comment 2.** In the in situ ATR-FTIR spectroscopy results, the authors assign the peak near 1500 $cm^{-1}$ to the C–H bending vibration of $Ph\text{-}NH_2$ and use this as evidence for its formation, but the reviewer does not understand the reasoning behind this assignment. Furthermore, this peak is very weak in intensity and is close to the noise level in some spectra.

**Answer:** Thank you for the suggestion. We agree with the reviewer's comment that the peak near 1500 $cm^{-1}$ to the C–H bending vibration of $Ph\text{-}NH_2$ is weak in intensity. Therefore, we have reanalyzed the in situ ATR-FTIR spectra and supplemented our assignment with additional evidence. The characteristic peak at 1622 $cm^{-1}$ is assigned to the N–H bending vibration of the $–NH_2$ group in $Ph\text{-}NH_2$, serving as reliable supplemental evidence for $Ph\text{-}NH_2$ formation (**Fig. R5 and Revised Fig. 3 a-b**) (***Nat. Commun.*** 2024, 15, 3195; ***Chem. Eng.*** 2024, 482, 149054). The peak is significantly more intense, well above the noise level, providing clear and direct evidence for the formation of $Ph\text{-}NH_2$. The corresponding revisions and references (**Ref. 33**) have been made in the revised manuscript.

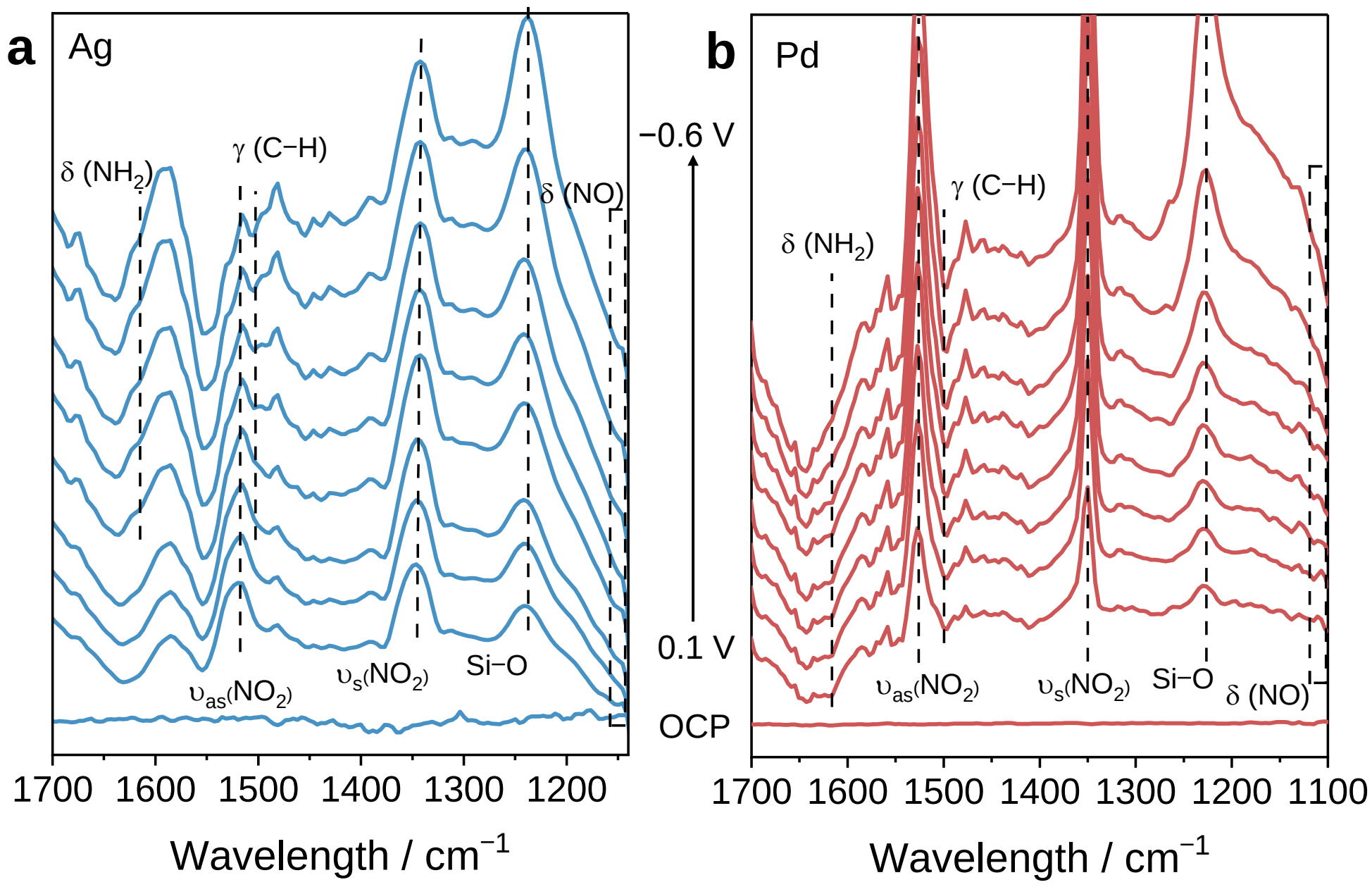


**Fig. R5 (Revised Fig. 3 a-b)** In situ ATR-FTIR spectra under various bias potentials over Ag and Pd.

**Comment 3.** The authors couple cathodic Ph-$NO_2$ reduction with anodic ascorbic acid oxidation (AOR) to significantly reduce the cell voltage. While this is an elegant strategy for energy savings, a critical practical concern needs to be addressed: what is the economic viability of using ascorbic acid as a sacrificial agent on a large scale? Ascorbic acid is relatively expensive, and its oxidation product (dehydroascorbic acid) would need to be separated and potentially discarded. Have the authors considered the cost of the ascorbic acid consumed in the process? A more comprehensive cost-benefit analysis that includes the expense of the anode reactant, rather than just the electricity savings, would be necessary to truly assess the industrial potential.

**Answer:** Thank you for the comment. We fully agree that the full-scale cost–benefit analysis proposed by the reviewer is indeed a prerequisite for evaluating the application potential of this system. Therefore, we have supplemented the cost calculation for the consumption of ascorbic acid (AA) (AA price: 12 $/kg). On the basis of the optimal reaction conditions (a current of 1.5 A, assuming 100% FE of AOR), the AA consumption per kilogram of target Ph-NHOH product is 4.07 kg, corresponding to a raw material cost of 48.93 $ per kg of Ph-NHOH. In accordance with the reviewer's suggestion, we have subsequently calculated the disposal cost for the oxidation product (dehydroascorbic acid, DHA) and clarified that a mature market for large-scale industrial application for DHA is lacking and that it cannot generate revenue through sales. The results confirm the reviewer's concern that under current laboratory-scale conditions, the cost of AA and the disposal of

DHA are not yet offset by electricity savings (**Fig. R6a-b**). We fully recognize this as a key bottleneck for the industrial application of AA-based systems, a challenge common to many sacrificial-agent-assisted electrocatalytic processes.

In response to this core bottleneck, we have further completed the optimization and upgrading of the system in the revised manuscript. In fact, replacing AA with such low-cost substrates is the direct path toward practical application. The same coupling strategy can be extended to cheap and abundant anodic reactants, such as glycerol (a biodiesel byproduct, <1.0 $/kg for industrial grad). We replaced the anodic coupled reaction from AA oxidation with a glycerol electrooxidation reaction and completed all relevant experimental verification and data supplementation (**Fig. R6c–d**). Glycerol, as a bulk byproduct of the biodiesel industry, has a wide range of sources and an extremely low cost. Moreover, glycerol electrooxidation can directly produce high-value-added fine chemicals such as formic acid and lactic acid, which reduces the need for additional waste product disposal costs and instead greatly improves the comprehensive economic benefit of the system. In addition, the optimized coupled system still retains the core advantage of significantly reducing the electrolytic cell voltage and electric energy consumption, which fundamentally solves the economic pain point of large-scale application raised by the reviewer. A full cost–benefit comparison between this coupled system and the traditional OER system is provided in the revised SI. We hope these additions and clarifications put our work in the right context without ignoring practical challenges. Thank you again for the thoughtful comments.

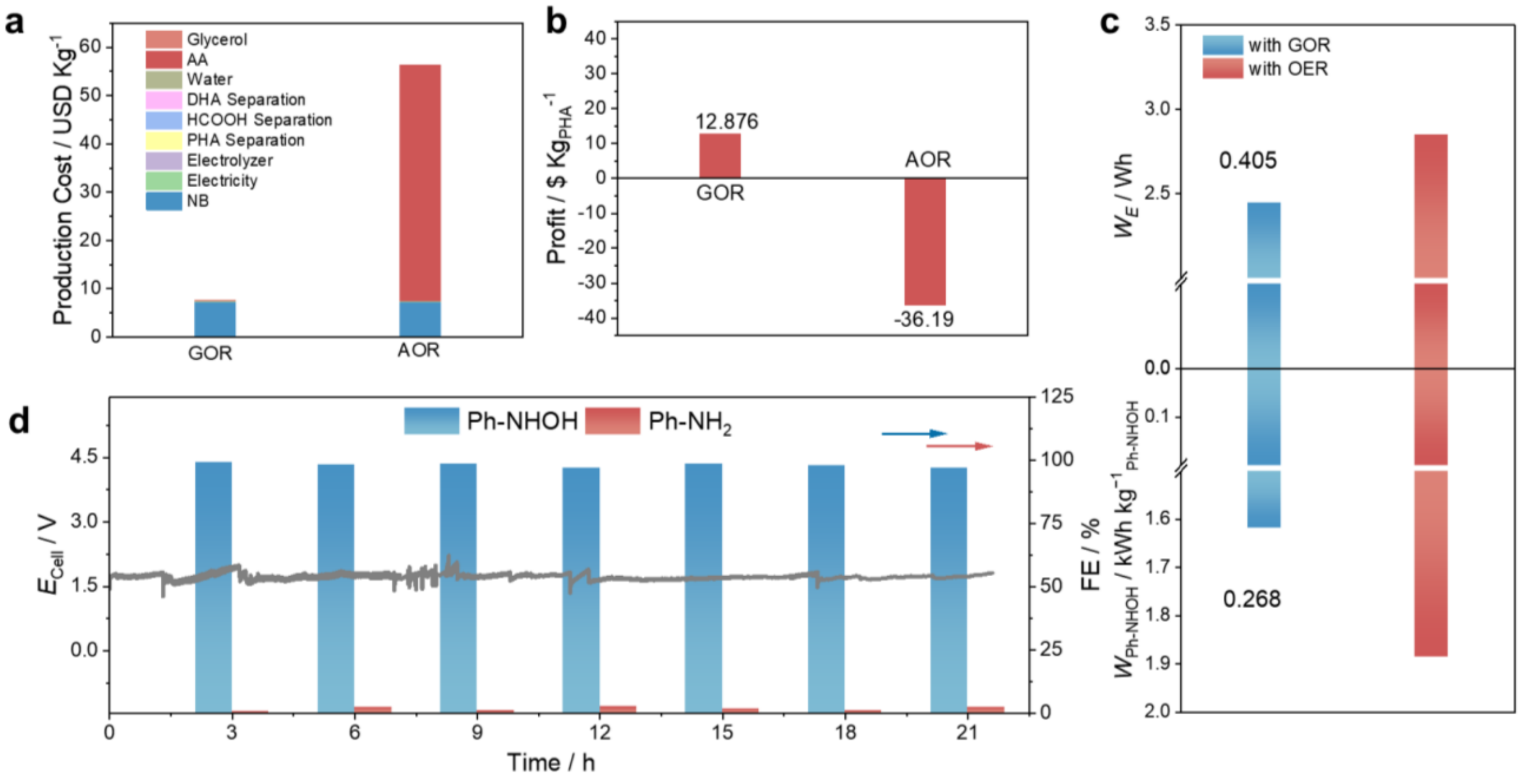


**Fig. R6 a,** Cost breakdown for production. **b,** Comparison of profitability.

**Comment 4.** The authors attribute the lowest Ph-NHOH selectivity on the Pd electrode (51.7%) primarily to the strong adsorption of Ph-NHOH on its surface, which promotes overhydrogenation to Ph-$NH_2$. However, Pd is also well-known for its excellent hydrogen evolution reaction (HER) activity and ability to provide high coverage of adsorbed hydrogen (*H). Given this, how can the authors definitively rule out the possibility that the low selectivity on Pd is, at least in part, due to a higher surface coverage of *H, which could accelerate the hydrogenation steps? Could the authors decouple the *H coverage effect from the Ph-NHOH adsorption strength, perhaps by comparing the HER activity or performing *H titration experiments on the different metal electrodes?

**Answer:** Thank you for the comment. We fully agree that Pd has excellent HER activity and can provide a high surface coverage of *H on its surface. As *H serves as the core hydrogen source for both Ph-$NO_2$ reduction reactions, its surface coverage inevitably affects the hydrogenation reaction process. In the original manuscript, we emphasized the effect of the strong adsorption of Ph-NHOH on the Pd surface on the over-hydrogenation reaction but did not fully discuss the influence of *H coverage. On the basis of the following considerations, we attribute the low selectivity on Pd primarily to the strong adsorption of Ph-NHOH rather than to *H coverage alone.

First, if *H coverage was the dominant factor, we would expect a strong correlation between HER activity (or *H coverage) and Ph-NHOH selectivity across all studied metals (**Fig. 2b**). For example, Pt with high HER activity is expected to exhibit similarly low selectivity, but Pt does not show such low Ph-NHOH selectivity. Au has a much lower HER activity than Cu does, yet both show similarly high Ph-NHOH selectivity. This lack of correlation further supports that the intrinsic adsorption strength of Ph-NHOH is the key selectivity determinant.

Second, to further decouple the *H coverage effect from the Ph-NHOH adsorption strength, we conducted pH-dependent electrocatalytic experiments at the same potential (**Fig. R7**). The potential ensures a consistent thermodynamic driving force for the target reaction across all the tested pH conditions, while the varied proton activity modulates the steady-state *H coverage on the Pd electrode by altering the Volmer step kinetics of the HER. The results show that the Ph-NHOH selectivity on Pd remains at a similarly low level across different pH conditions, indicating that varying *H coverage does not significantly change the selectivity. This provides direct experimental evidence that *H coverage is not the dominant factor governing over-hydrogenation on Pd.

On the basis of the above analysis, we have revised the manuscript accordingly to include this more balanced discussion and additional pH-dependent experimental data. We thank the reviewer again for helping us strengthen our mechanistic analysis.

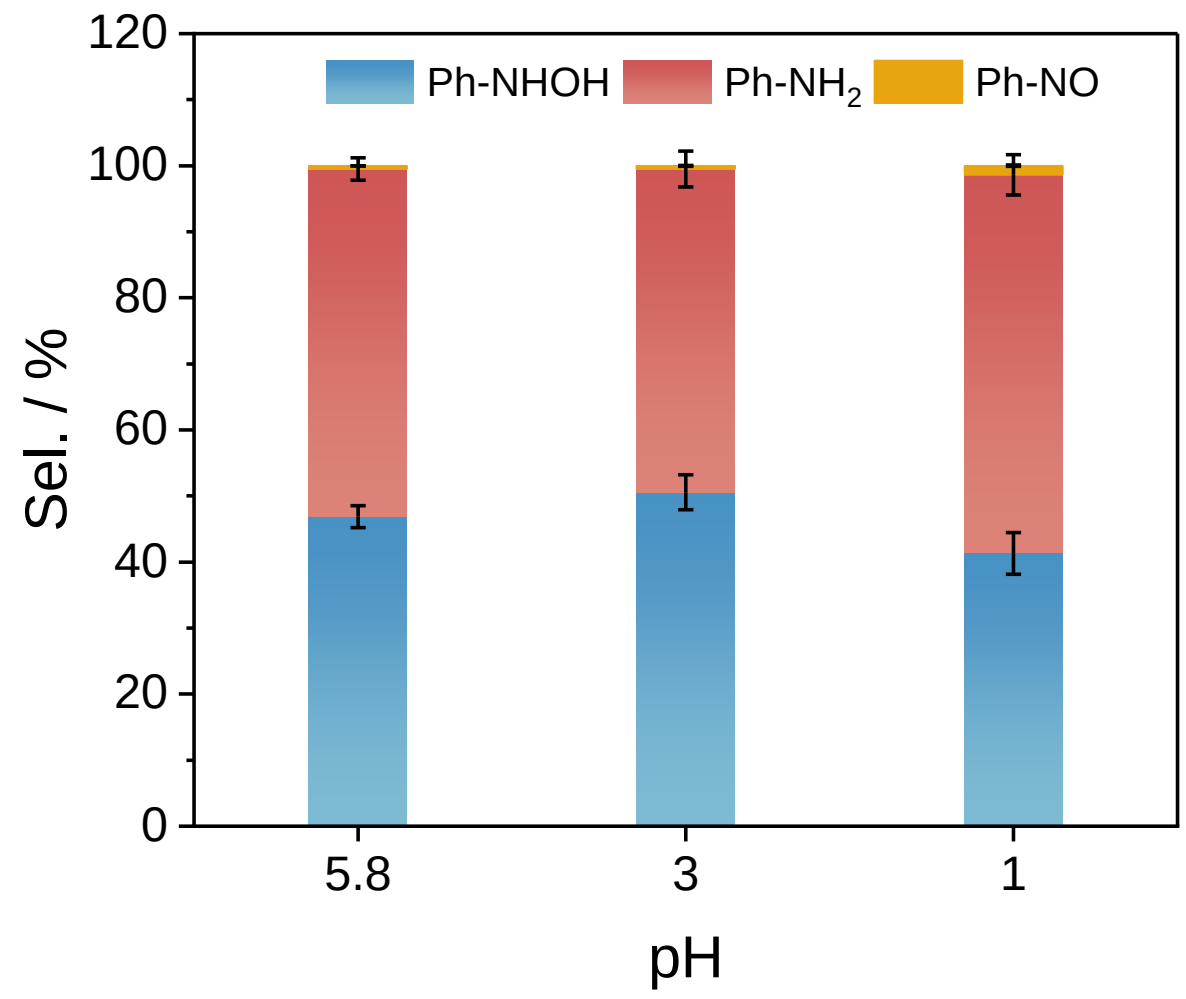


**Fig. R7** Product distribution over Pd in different pH electrolytes.

**Comment 5.** The electrochemical measurements were primarily conducted in a 0.5 M PBS buffer at pH=5.8. Given the proton-coupled electron transfer (PCET) nature of the reaction, a systematic investigation of the pH effect would strengthen the mechanistic understanding. The authors are requested to provide data on the Ph-$NO_2$ conversion, Ph-NHOH selectivity, and FE for the CV-Ag catalyst across a wider pH range (e.g., from strongly acidic pH=1 to near-neutral pH=7). This is particularly important to determine if the chemical stability of Ph-NHOH becomes a limiting factor under more acidic conditions, which would be crucial for process design.

**Answer:** Thank you for the comment. As the reviewer suggested, we have conducted additional electrocatalytic measurements over the CV-Ag catalyst across a wider range of pH values from acidic to neutral (pH 1.0–7.0). The results demonstrate that the Ph-$NO_2$ conversion remains at approximately 100% and that the Ph-NHOH selectivity is consistently above 90% in 1.0–6.0 pH electrolytes. However, when the pH is 7.0, the Ph-NHOH selectivity significantly decreases to approximately 83%. The FE follows a similar trend, decreasing to 85%. We attribute the decreased selectivity under alkaline conditions to the chemical instability of Ph-NHOH, which undergoes base-catalyzed decomposition or oxidation, leading to the formation of azoxy or azo byproducts (**Supplementary Fig. S2**). This is also proven by the control experiment. This confirms that the low selectivity under alkaline conditions is primarily due to the chemical instability of Ph-NHOH rather than changes in the electrocatalytic pathway. Although our pH-dependent experiments confirm that the CV-Ag catalyst also exhibits high conversion and selectivity under strongly acidic conditions, we

selected a pH of 5.8 as our primary reaction condition, considering electrode stability, system compatibility, and postprocessing convenience.

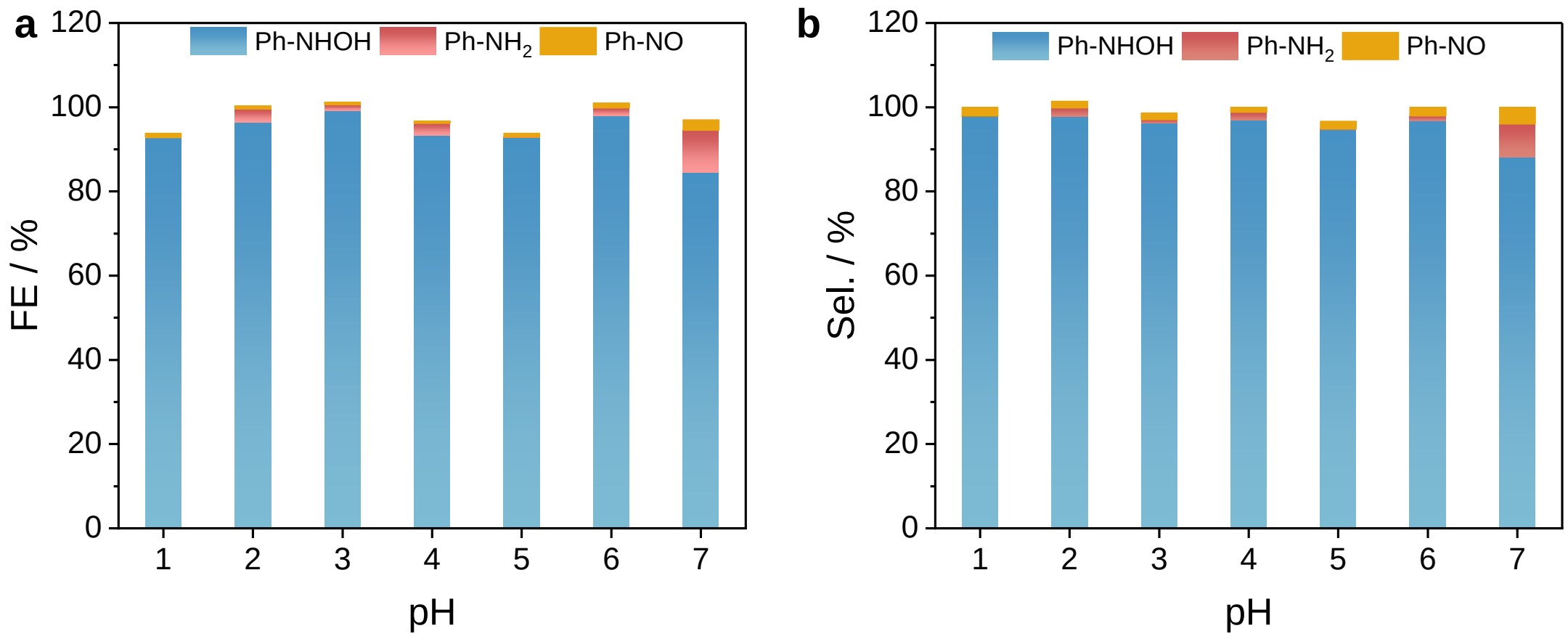


**Fig. R8** Product distribution over the CV-Ag in different pH electrolytes.

**Comment 6.** The enhanced performance of the CV-Ag catalyst is primarily attributed to its enlarged electrochemical surface area (ECSA). While ECSA is certainly a factor, the unique surface structure of electrodeposited nanoparticles, such as grain boundaries, defects, and undercoordinated sites, can also impart distinct intrinsic activity. How do the authors exclude the possibility that the improved performance of CV-Ag, compared to bulk Ag, stems from these unique surface atomic configurations rather than just an increase in the number of active sites? A normalization of the activity by ECSA (i.e., specific activity) is already provided, but a more direct characterization of the surface defects (e.g., using lead underpotential deposition or high-resolution TEM with geometric phase analysis) and correlating this with the weak adsorption property would be insightful.

**Answer:** Thank you for the comment. We agree that surface defects and undercoordinated sites may influence the intrinsic activity of Ag catalysts. To directly verify the effect of surface atomic configurations on catalytic performance, we synthesized a defective Ag catalyst with abundant low-coordination sites (OD-Ag) (***Nat. Catal.*** 2023, 6, 906–915). EXAFS analysis revealed an average coordination number of 7.2 for OD-Ag. The electrochemical test results show that OD-Ag is more selective toward Ph-$NH_2$, indicating that OD-Ag promotes overhydrogenation and leads to lower Ph-NHOH selectivity (**Fig. R9**). These results suggest that excessive defect density and low-coordination sites increase the adsorption strength of the intermediate Ph-NHOH on the Ag surface, thereby accelerating its further hydrogenation to Ph-$NH_2$. These results indicate that excessive defects are detrimental to selective Ph-NHOH formation (***Nat. Chem.*** 2025, 17, 1152–1160). In contrast, the

surface defect density of CV-Ag is low (**Supplementary Table S3**). Thus, we conclude that the enhanced Ph-$NO_2$-to-Ph-NHOH conversion of CV-Ag relative to that of bulk Ag is attributed primarily to the increased number of active sites (larger ECSA) rather than to the optimization of intrinsic activity by surface atomic configurations.

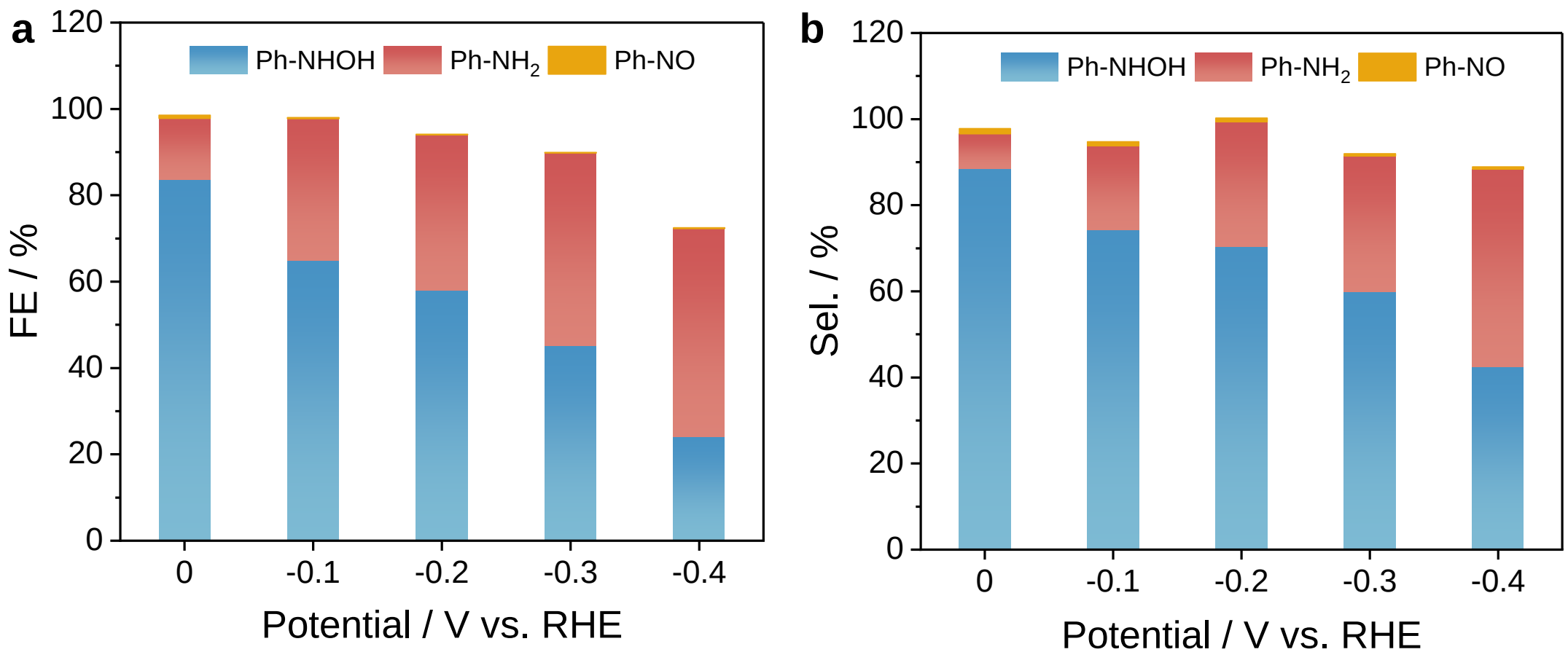


**Fig. R9** Electrochemical performance of OD-Ag.

**Comment 7.** The technoeconomic analysis (TEA) presented in the manuscript, estimating a profit of $520 per tonne of Ph-NHOH, is a strong point. However, this analysis appears to be based primarily on electricity and feedstock costs. It seems to omit the significant costs associated with downstream processing, specifically the product separation and purification steps. The Methods section describes extraction with ethyl acetate and recrystallization using petroleum ether and dichloromethane. These unit operations involve substantial solvent costs, energy for distillation/recovery, and waste management. The authors are requested to provide a revised TEA that explicitly includes the costs of these downstream separation processes to present a more realistic "plant-gate" cost and a fairer comparison with existing chemical routes.

**Answer:** We acknowledge the reviewer's constructive comments and suggestions. We apologize for the imperfectly presented technoeconomic analysis model. In accordance with the reported TEA model (***Nat. Sustain.***, 2021, 4, 911-919), we estimated the costs of extraction and recrystallization purification processes via the scaling method. To address the core economic bottleneck of the ascorbic acid oxidation system, we replaced the anodic reaction with glycerol electrooxidation and completed a comprehensive full-process TEA for this optimized coupled system using the same standardized model. The revised TEA results confirm that the glycerol oxidation coupled system

achieves a significant improvement in economic performance and clearly exhibits industrial profit potential. The amended calculation details are extracted below:

Taking the 5000 kg daily capacity of Ph-NHOH as an example at a current density of 0.12 A $cm^{-2}$ with 99% FE of Ph-NHOH as an example.

The specific assumptions made for the TEA are listed as follows:

The electricity price was considered to be 0.04 \$ $kWh$.

The operating voltage was set as 1.63 V.

The electrolyzer cost per area was calculated as follows:

$$Electrolyzer\ Cost\ per\ area\ = 275\ \$\ kW^{-1} \times \frac{0.12\ A}{cm^2} \times 1.63\ V \times \frac{10^4 cm^2}{m^2} \times \frac{kW}{1000} = 537.9\ \$\ m^{-2}$$

The anticipated service life of the electrolyzer was 20 years, and the working time per year was 350 days. It is assumed that the electrolyzer stacks do not have to be replaced during the lifetime of the plant.

4, The separation cost is composed of two parts: the separation cost of Ph-NHOH and that of HCOOH. We set the Ph-NHOH separation yield at 80%.

Thus, the parameters needed in the cost calculation are determined as follows:

$$The\ Current = \frac{5000\ Kg}{day} \times \frac{day}{86400\ s} \times \frac{1000g}{Kg} \times \frac{mol}{109\ g} \times 4e^- \times \frac{96485\ C}{mol} \times \frac{1}{0.99} \times \frac{1}{0.8} = 258716.64\ A$$

$$\mathrm{Ph-NO_2}\ Weight = 258716.64\ A\ \times \frac{1}{4e^-} \times \frac{1\ mol}{96485\ C} \times \frac{0.123\ Kg}{mol} \times \frac{86400\ s}{day} = 7123.99\ Kg\ day^{-1}$$

$$Glycerol\ Weight = 258716.64\ A\ \times \frac{1}{8e^-} \times \frac{1\ mol}{96485\ C} \times \frac{0.092\ Kg}{mol} \times \frac{86400\ s}{day} \times 0.4 = 1065.71\ Kg\ day^{-1}$$

$$HCOOH\ Weight = 258716.64\ A\ \times \frac{1}{8e^-} \times 3 \times \frac{1\ mol}{96485\ C} \times \frac{0.046\ Kg}{mol} \times \frac{86400\ s}{day} \times 0.4$$

$$= 1598.55\ Kg\ day^{-1}$$

$$Electrolyzer\ Area = 258716.64\ A \times \frac{1}{0.12\ A\ cm^{-2}} \times \frac{m^2}{10^4\ cm^2} = 215.60\ m^2$$

$$Power\ = 1.63\ V\ \times 258716.64\ A \times \frac{MW}{10^6 W} = 0.422\ MW$$

The Ph-$NO_2$ flow rate is as follows:

$$\mathrm{Ph-NO_2}\ flow\ rate = 7123.99\ \frac{Kg}{day} \times \frac{m^3}{1205\ Kg} \times \frac{day}{24\ h} = 4.11\ \frac{L}{min}$$

The glycerol flow rate is as follows:

$$Glycerol\ flow\ rate = 1065.71\ \frac{Kg}{day} \times \frac{m^3}{1250\ Kg} \times \frac{day}{24\ h} = 0.036\ \frac{L}{min}$$

It is assumed that the Ph-NHOH and HCOOH electrolytes are recycled until they reach a steady-state volume concentration of 10%. The continuous exit electrolyte flow rate is as follows:

$$Ph-NHOH\ \ Electrolyte\ \ flow\ \ =4.11\ \frac{L}{min}\ \times\ \frac{1}{0.1}=41.1\frac{L}{min}$$

$$HCOOH\ \ Electrolyte\ \ flow\ \ =0.036\ \frac{L}{min}\ \times\ \frac{1}{0.1}=0.36\frac{L}{min}$$

**Cost:**

Electrolyzer:

$$Electrolyzer\ \ Cost=537.9\ \ \$\ \ m^{-2}\times 215.60\ \ m^{2}\ \ \times\ \frac{1}{20\ \ year}\times\frac{1}{350\ \ day}=16.57\ \ \$\ \ day^{-1}$$

Electricity:

$$Electricity\ \ Cost\ \ =0.422\ \ MW\times\ \frac{1000\ \ kW}{MW}\ \times\frac{24\ \ h}{day}\ \times\frac{0.04\ \ \$}{kWh}=438.72\ \ \$\ \ day^{-1}$$

Separation cost:

$$\mathrm{Ph-NHOH\ \ Separation\ \ Cost}=4162240\ \ \$\times\left(\frac{41.1\ \frac{L}{min}}{1000\ \frac{L}{min}}\right)^{0.7}\ \times\ \frac{1}{20\ \ year}\times\frac{1}{350\ \ day}=63.78\ \ \$\ \ day^{-1}$$

$$\mathrm{HCOOH\ \ Separation\ \ Cost}=4162240\ \ \$\times\left(\frac{0.36\ \frac{L}{min}}{1000\ \frac{L}{min}}\right)^{0.7}\ \times\ \frac{1}{20\ \ year}\times\frac{1}{350\ \ day}=2.31\ \ \$\ \ day^{-1}$$

Material cost:

$$\mathrm{Ph-NO_2}\ \ Cost=7123.99\ \ Kg\ \ day^{-1}\times 5\ \ \$\ \ Kg^{-1}=35619.95\ \ \$\ \ day^{-1}$$

$$Glycerol\ \ Cost=1065.71\ \ \ Kg\ \ day^{-1}\times 0.97\ \ \$\ \ Kg^{-1}=1033.74\ \ \$\ \ day^{-1}$$

$$Water\ \ Cost=258716.64\ \ A\times 0.6\times\frac{1}{4e^{-}}\times\frac{mol}{96485\ \ C}\times\frac{0.018\ \ Kg}{mol}\ \times\frac{0.2642\ \ gal}{Kg}\times\frac{86400\ \ s}{day}\ \times\frac{0.0054\ \ \$}{gal}$$

$$=0.89\ \ \$\ \ day^{-1}$$

$$Total\ \ Cost=Electrolyzer\ \ Cost+Electricity\ \ Cost+\mathrm{Separation\ \ Cost}+Water\ \ Cost+Material\ \ Cost$$

$$Total\ \ Cost=\frac{16.57+438.72+63.78+2.31+35619.95+1033.74+0.89}{5000}=7.44\ \ \$\ \ day^{-1}Kg_{Ph-NHOH}^{-1}$$

$$Profit=20+\frac{1598.55\ \ \times\ \ 0.97}{5000}-7.44=12.87\ \ \$\ \ day^{-1}Kg_{Ph-NHOH}^{-1}$$

**Comment 8.** The substrate scope demonstrates excellent compatibility with halogens, methyl, and cyano groups. To truly define the application boundaries of this Ag-catalyzed system, it is essential to test its tolerance towards more sensitive functional groups. The authors are encouraged to expand the substrate scope to include nitroaromatics bearing easily reducible groups, such as esters (e.g., methyl 4-nitrobenzoate), aldehydes (e.g., 4-nitrobenzaldehyde), or ketones (e.g., 4-nitroacetophenone). These experiments would clarify whether the high selectivity for the

hydroxylamine can be maintained when competing, more electrophilic sites are present in the molecule, which is a common challenge in organic synthesis.

**Answer:** Thank you for the suggestion. Following the reviewer's recommendation, we performed additional reduction experiments on three representative nitroaromatics with easily reducible functional groups, namely, methyl 4-nitrobenzoate (ester group), 4-nitrobenzaldehyde (aldehyde group), and 4-nitroacetophenone (ketone group). The experimental results show that all three substrates are smoothly converted to the corresponding hydroxylamine products, with the selectivity exceeding 90% in all the cases (**Fig. R10**). Notably, even in the presence of competitive reducible sites (aldehyde and ketone groups), the nitro group is preferentially reduced, and no significant reduction products of the aldehyde or ketone groups are observed. With respect to the ester-containing substrate, the ester group remains intact during the reaction without hydrolysis or reduction, indicating that this system has good compatibility. Therefore, our Ag catalytic system exhibits good functional group tolerance and high hydroxylamine selectivity for nitroaromatics bearing ester, aldehyde, and ketone groups. These results have been added to the substrate scope section of the revised manuscript (**Revised Fig. 4e** and **Supplementary Fig. S32-34**).

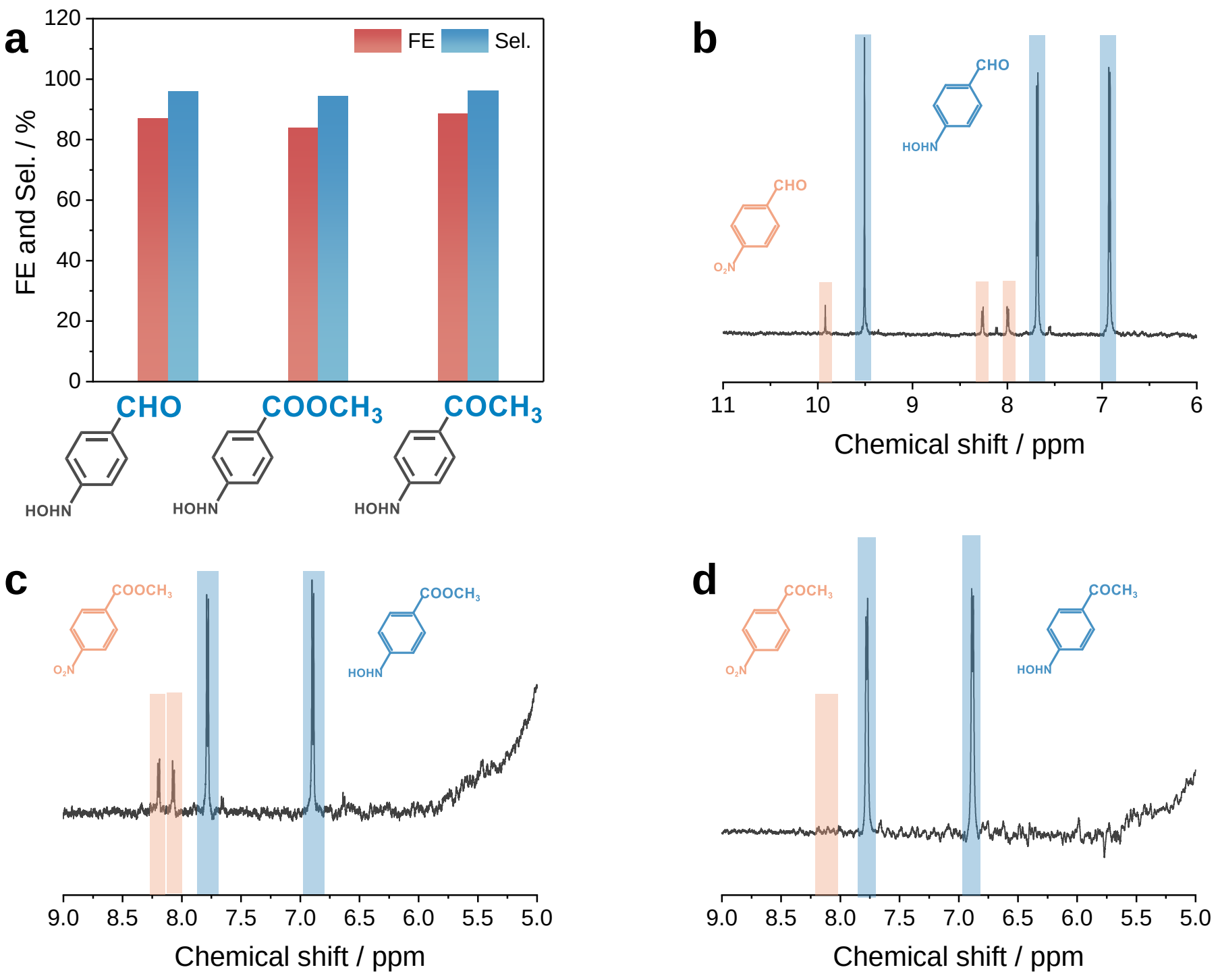


**Fig. R10 a,** Synthesis of functionalized *N*-phenylhydroxylamine derivatives over CV-Ag. **b–d,** $^1$H NMR spectra of the crude solution.

**Comment 9.** The long-term stability test over 20 hours at 1.5 A is impressive and crucial for practical application. However, several operational details are missing. Firstly, how was the nitrobenzene reactant replenished during this 20-hour period to maintain a constant concentration? Was it a batch process with periodic replacement of the catholyte, or was a continuous flow system used? Secondly, and more importantly, a post-mortem characterization of the CV-Ag catalyst after this prolonged electrolysis is necessary. The authors should provide SEM images to check for morphological changes (e.g., nanoparticle agglomeration or detachment), XPS analysis to examine any changes in the chemical state or surface composition (e.g., oxidation or poisoning), and ICP-MS analysis of the electrolyte to quantify any potential leaching of Ag. This data is vital to substantiate the claim of excellent durability.

**Answer:** Thank you for your comment, which helps improve the rigor of our study and fully substantiates the excellent durability of the CV-Ag catalyst for practical applications. We have completed all the corresponding revisions and supplementary experiments as detailed below.

1. Operational details of reactant replenishment during the 20-hour stability test: Long-term electrolysis was conducted in batch operation mode with periodic catholyte replacement. Specifically, during continuous electrolysis at 1.5 A for more than 20 hours, the catholyte was completely replaced with freshly prepared electrolytes every 5 hours. This operational detail has been added to the revised manuscript as the following sentence: "Long-term electrolysis was conducted at a constant current for 20 hours in batch operation mode, during which the catholyte was completely replaced with freshly prepared electrolytes every 5 hours."

2. Characterization of the CV-Ag catalyst after electrolysis: In accordance with the reviewer's suggestion, we performed postelectrolysis characterization of the CV-Ag catalyst using SEM, XPS, and ICP-MS to comprehensively evaluate its structural and compositional stability. The SEM images show that the original morphology of the catalyst was maintained after high-current electrolysis, with no obvious nanoparticle agglomeration, detachment, or structural collapse (**Fig. R11a–d**). The XPS results indicate that the characteristic Ag 3d binding energy peaks did not shift before or after electrolysis. No additional peaks corresponding to $Ag^{+}$ were detected, indicating that the chemical state of Ag remained stable without significant changes in valence during long-term operation (**Fig. R11e**). The ICP–MS analysis results indicate that the Ag leaching concentration in the electrolyte was extremely low and negligible (<0.68 ppb), demonstrating the outstanding anti-leaching stability of the CV-Ag catalyst (**Table R1**). All the above results confirm the excellent durability of the CV-Ag catalyst, comprehensively addressing the reviewer's concerns. The corresponding data and

discussion have been incorporated into the revised manuscript (**Supplementary Fig. S42 and Table S5**).

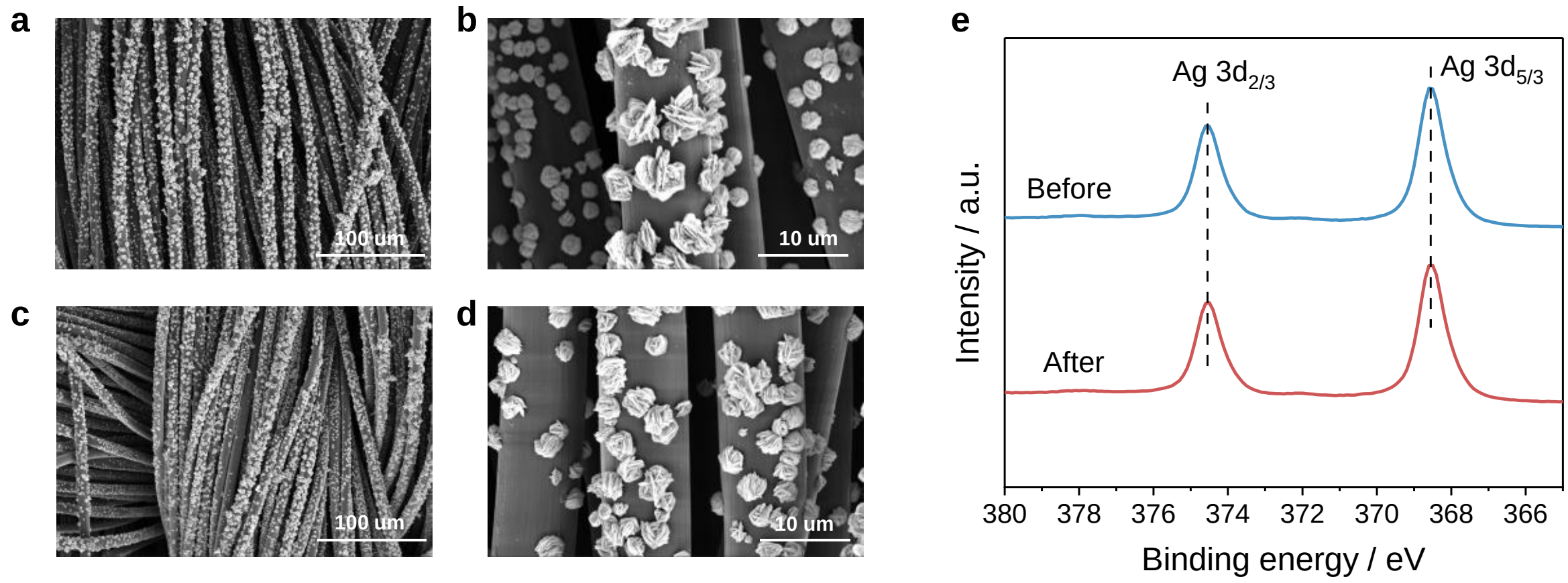


**Fig. R11 (Supplementary Fig. S42) a-b,** SEM images of CV-Ag deposited on carbon cloth. **c–d,** SEM images of CV-Ag after long-term electrolysis. **e,** XPS spectra of CV-Ag before and after long-term electrolysis.

**Table R1 (Supplementary Table S5)** ICP–MS analysis of the Ag content in the electrolyte before and after 20 h of long-term electrolysis

| Element | Before | After |
|---|---|---|
| Ag | 0.64 ppb | 0.68 ppb |

## To Reviewer 3:

## Reviewer Letter:

Lu et al. demonstated a selective electrochemical route for converting Ph-$NO_2$ to high-value Ph-NHOH. This study highlights the pivotal role of Ag in enabling weak adsorption of Ph-NHOH, which is supported by the results of DFT and in situ FTIR. Then, the authors successfully achieved highly selective electrosynthesis of Ph-NHOH, overcoming the thermodynamic unfavorability of its formation. This innovative and impactful study advances our understanding of selectivity control in Ph-NHOH electrosynthesis. Accordingly, this work is suitable for publication in Nature Communications once the following issues have been addressed.

**Answer:** We thank the reviewer for the positive assessment of our work. With respect to the concerns or comments of the reviewer, we have provided point-by-point responses. To save the reviewer's valuable time, key revisions are displayed on a yellow background in the revised manuscript and Supplementary Information (SI).

**Comment 1.** When the adsorption energies of Ph-NO, Ph-NHOH, and other intermediates are calculated, multiple adsorption modes are possible, which may influence the selectivity among Ph-NHOH, Ph-$NH_2$, and azoxy products. Which configuration is adopted for each intermediate in the DFT discussion? Does the benzene ring participate in flat-lying adsorption or -$NO_2$-centered vertical adsorption?

**Answer:** We sincerely appreciate the reviewers' valuable suggestions. To obtain the globally stable adsorption configuration for each species, we systematically examined various configurations of all the adsorption intermediates, and the optimal configurations are presented in Supplementary Figure S3. Owing to the distinct electronic structures of different metals, their adsorption capacities toward the same intermediate vary significantly, accompanied by differences in adsorption configurations.

For postshock metals (Cu and Ag), the adsorbed intermediates exhibit weak adsorption energies and adopt physical adsorption configurations. For example, *Ph-$NO_2$ adsorbs in a flat-lying manner on the surface with an adsorption distance exceeding 3 Å. In contrast, intermediates adsorbed on Pt and Pd display stronger adsorption energies; for example, *Ph-$NO_2$ binds to the surface via its benzene ring, whereas the −$NO_2$ functional group does not form chemical bonds with the surface. When adsorbed on Ru, the adsorption capacity is further enhanced. For *Ph-$NO_2$ specifically, both

the benzene ring and the $-NO_2$ group are chemically bound to the surface; however, the excessively strong adsorption capability induces the dissociation of $-NO_2$ into *NO and *O species.

Additionally, competitive intermediates (e.g., *Ph-NOH and *Ph-NHO) have been evaluated in the identified reaction pathway to clarify their potential effects.

**Comment 2.** In the context of this multistep reaction system, the choice of Ph-NO adsorption energy as a universal descriptor requires further justification, particularly given that the adsorption/desorption behavior of Ph-NHOH governs selectivity.

**Answer:** We sincerely thank the reviewer for noting that the adsorption/desorption behavior of Ph-NHOH may be a key step influencing the reaction selectivity. We first emphasize that our goal is to characterize the activity selectivity by the difference in activity between the main reaction and side reactions (Fig. 2a). Here, we select the adsorption energy of *Ph-NO as the descriptor to evaluate the activity of both the main and side reactions on the basis of the following two considerations:

1. Scaling relationship: Evaluating reaction activity for multistep reactions is inherently complex. Fortunately, the d-band center theory and volcano plot proposed by Nørskov and coworkers (***Nature*** 1995, 376, 238; ***Nat. Catal.*** 2023, 6, 460−461; ***J. Phys. Chem. B*** 2004, 108, 17886−17892) have provided a feasible solution. This theory leverages the linear scaling relationship (LSR) between the adsorption energies of any two intermediates, enabling the adsorption energy of any intermediate to be expressed as a linear function of that of a single intermediate (***Nat. Rev. Mater.*** 2019, 4, 792−804; ***Phys. Rev. Lett.*** 2012, 108, 116103; ***Phys. Chem. Chem. Phys.*** 2011, 13, 20760−20765; ***Angew. Chem. Int. Ed.*** 2008, 47, 4683−4686). Mathematically, the representation of all intermediates via a single descriptor is only valid when such a linear scaling relationship holds. Once the fitted linear scaling relationship is statistically significant (e.g., $R^2 > 0.8$), the selected intermediate can serve as a reliable descriptor.

2. Chemical rationality: While the mathematical validity of the descriptor is confirmed by the linear scaling relationship, its chemical relevance must also be justified. *Ph-NO is an indispensable intermediate in the entire reaction pathway, making it applicable as a universal descriptor for all catalysts under investigation. In contrast, if the adsorption energy of *Ph-NHOH was chosen as the descriptor, potential issues would arise: for certain catalysts, *Ph-NHOH cannot exist stably (e.g., it dissociates into *Ph-NH and *OH), which would hinder the accurate quantification of activity for these catalysts. Such a strategy is common in previous works (***Science*** 2008, 320, 1320--1322; ***Nature***,

2020, 581, 178−184; ***Nat. Commun.*** 2017, 8, 15438; ***J. Phys. Chem. Lett.*** 2012, 3, 251−258; ***Phys. Chem. Chem. Phys.*** 2014, 16, 4720−4727).

Thus, we select *Ph-NO as the descriptor. The linear scaling relationships of the adsorption energies are presented in **Supplementary Fig. S4**, where all $R^2$ values are approximately 0.8. This confirms the rationality and reliability of *Ph-NO as an activity descriptor.

**Comment 3.** For the mechanistic analysis, all figures presented in the Supporting Information require detailed captions, including experimental conditions, to make them understandable to a general audience.

**Answer:** Thank you for the comment. We fully accept this comment and have made the corresponding revisions. Specifically, we have systematically checked all the figures related to the mechanistic analysis in the Supporting Information and supplemented each figure with a detailed caption. These revised captions include complete experimental conditions and clear descriptions of the content, ensuring that the figures are understandable to a general audience.

**Comment 4.** In Figure 22, the LSV curve shows obvious current fluctuation after adding Ph-$NO_2$, and can it be avoided?

**Answer:** Thank you for the comment. We have made multiple experimental attempts to avoid current fluctuations. The current fluctuation originates from the constant magnetic stirring applied during the LSV tests. Unfortunately, this fluctuation cannot be eliminated without compromising the scientific validity of the test data. If the stirring was stopped to obtain a smoother LSV curve, the resulting current data would be severely affected by concentration polarization, which would fail to reflect the intrinsic reaction kinetics and the real electrocatalytic performance of the catalyst.

**Comment 5.** For the initial experiments that established Ag as the volcano-apex metal, what were the detailed electronic structure and surface crystallography of the other metals tested?

**Answer:** Thank you for the comment. We fully agree that clarifying the structural information of different metal electrodes is important for properly interpreting the volcano-type trend. In our initial screening experiments, we tested a series of metal electrodes (e.g., Cu, Ag, Au, Pd, and Pt). To confirm their crystal phase, preferred orientation, and structural integrity, we collected X-ray diffraction (XRD) patterns for all the tested metals. The results show that each metal exhibits characteristic crystalline

diffraction peaks without obvious alloy formation or impurity phases, and the dominant facets are consistent with those of standard polycrystalline metal structures (**Fig. R12**). With respect to the electronic structure, we referred to the literature-reported d-band center and work function data. The order of the d-band centers is Pt > Pd > Cu > Au > Ag, which is consistent with the order of binding strength of reaction intermediates, thereby positioning Ag at the volcano apex (***J. Am. Chem. Soc.*** 2025, 147, 46729−46744). The work function data for each metal are also available in the literature (***J. Appl. Phys.*** 1977, 48, 4729−4733).

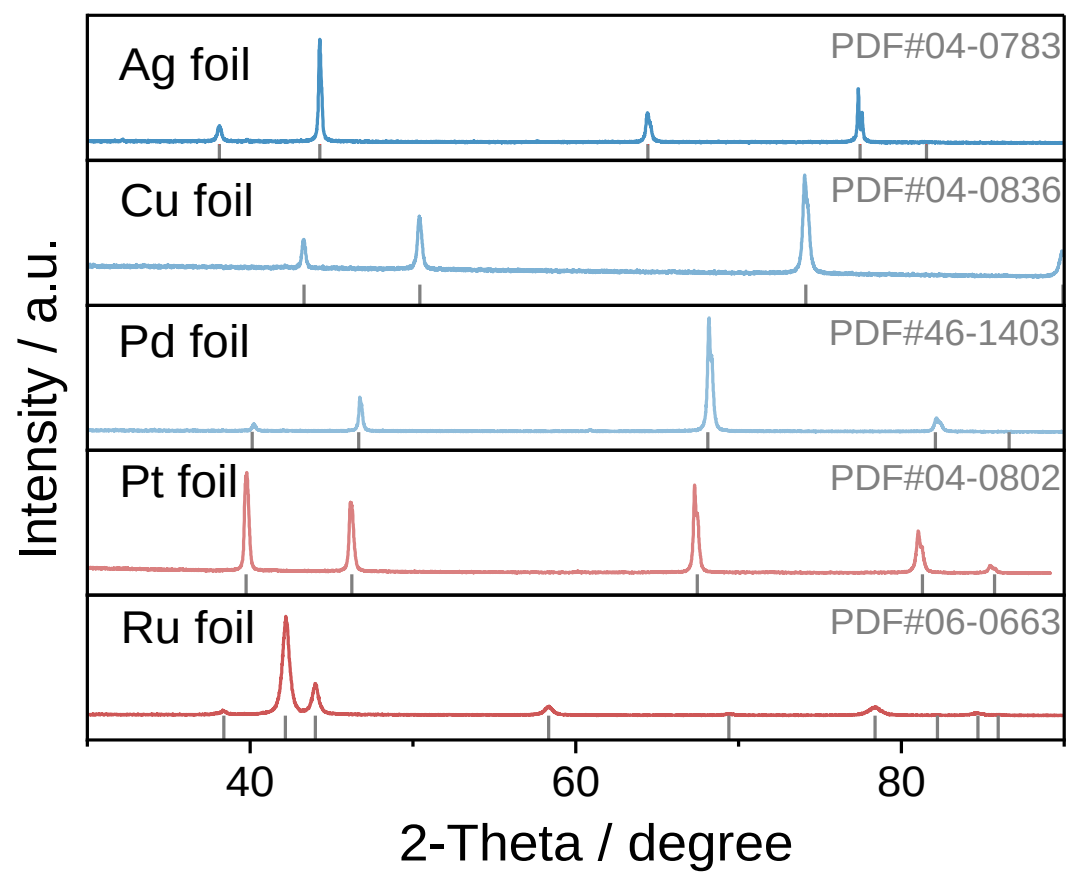


**Fig. R12** XRD patterns of the five metals.

**Comment 6.** To better illustrate the superiority of this method, the authors could focus the functional group compatibility discussion on several particularly challenging cases.

**Answer:** Thank you for the insightful comment. To better highlight the superiority of our method, we have added a functional group compatibility study on several particularly challenging substrates, including nitroarenes bearing aldehyde, ester, and ketone groups, which are prone to overreduction under conventional conditions (**Fig. R13**). The supplementary results demonstrate that our CV-Ag catalyst consistently delivers high selectivity and Faradaic efficiency toward the target *N*-phenylhydroxylamine product for all these challenging substrates. These results have been added to **Revised Fig. 4**.

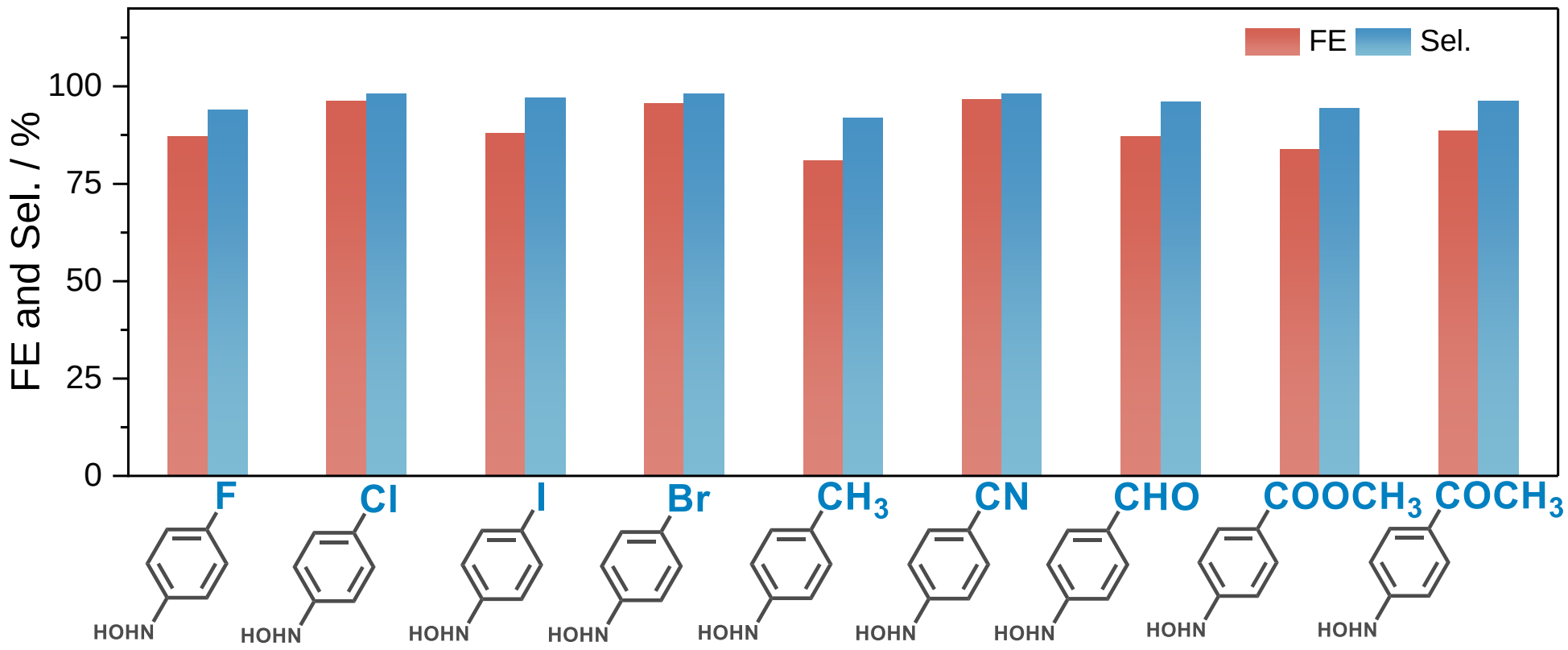


**Fig. R13 (Revised Fig. 4e)** Electrosynthesis of functionalized N-phenylhydroxylamine derivatives over CV-Ag.

## To Reviewer 4:

## Reviewer Letter:

This report makes a notable contribution to the field of electrosynthesis by optimising the reduction of nitrobenzene to phenylhydroxylamine, a valuable product. The topic is now of broader interest and the combination of experiment and theory is attractive. The outcome is demonstrated with a bulk scale synthesis clearly demonstrating the value of the work.

The study has good depth with a range of experimental methods including isotope effects. There are some odd choices like using the expensive ascorbic acid as sacrificial reagent. Some parts are simplified to a level that might mislead readers (e.g. important process parameters are not clear). But the text reads well and the results are generally important. Figures are provided in good quality, but figure captions need to give more information. Publication is recommended with a few revision suggestions.

**Answer:** We appreciate the reviewer's positive evaluation and constructive suggestions. With respect to the concerns or comments of the reviewer, we have carefully addressed all the concerns raised, including the use of ascorbic acid, the missing process parameters, and the figure captions. The detailed point-by-point responses are provided below. To save the reviewer's valuable time, key revisions are displayed on a yellow background in the revised manuscript and Supplementary Information (SI).

**Comment 1.** There is substantial previous literature which is ignored. In "Organic Electrochemistry" by Hamerich/Speiser this electrosynthesis is reported on page 1161 and two more references are provided: (1) Le Guyader in Bull. Soc. Chim. Fr. 1966, 1848 and (2) Brand in Ber. Deut. Chem. Ges. 1905, 38, 3077. This historical perspective should be included.

**Answer:** Thank you for the comment. We are grateful to the reviewer for bringing this highly relevant and excellent work to our attention. We fully agree that providing a comprehensive and fair literature context is essential, and we sincerely respect the historical contributions in this field. We have carefully studied the original French text of Le Guyader's work as well as the text of Brand's work and have cited them in the revised manuscript (**Refs. 17 and 18**).

**Comment 2.** From title and text, it appears as if simply selecting the right metal is the key to this electrosynthesis, but very important parameters here are pH, T, and the diffusion layer. Changing transport at the electrode surface usually changes the mechanism or the yield. It would be good if this could be acknowledged just for the benefit of the readers. What is the meaning to 800 rpm stirring in the experimental section?

**Answer:** Thank you for the comment. To investigate the intrinsic catalytic performance of different metals with a single-variable approach, we fixed key parameters, including the pH, reaction temperature, and interfacial mass transport conditions, in this work. The main text focused on the central role of metal selection but indeed omitted the elaboration of the importance of these parameters. We supplemented a statement on the critical impact of the pH, temperature, diffusion layer, and mass transport processes on the electrosynthesis reaction in the revised manuscript: "Given that the pH, temperature, and diffusion layer critically influence the reaction kinetics and mass transport, these parameters were fixed to ensure a fair comparison of the relative catalytic activities of the different bulk metal electrodes." Meanwhile, we supplemented more explicit experimental parameter details in the revised manuscript to improve its rigor and completeness, and to provide readers with more comprehensive information.

Regarding 800 rpm stirring in the electrochemical potentiostatic tests, this constant stirring was applied for two core purposes. First, to eliminate concentration polarization at the electrode surface caused by the rapid consumption of the substrate, stable and uniform mass transfer of the Ph-$NO_2$ substrate from the bulk electrolyte to the electrode surface was ensured, thereby allowing us to determine the intrinsic catalytic performance of the catalyst. Second, identical test conditions for all parallel catalyst samples were ensured by fixing a single rotation rate, thus eliminating the interference of mass transfer differences on the performance comparison between different catalysts.

**Comment 3.** An obvious electrode material to use in electrosynthesis is carbon (rather than noble metals). Was this considered? Where would it be in the volcano?

**Answer:** We thank the reviewer for this insightful comment. Carbon-based materials were indeed considered during our research design phase. To determine the position of the carbon in our activity volcano plot, we performed additional DFT calculations using the same activity descriptor and theoretical framework as in the original manuscript (**Fig. R14**). The calculated value places carbon on the weak adsorption branch of the volcano plot, near Ag, but slightly farther from the theoretical

apex than Ag. This position indicates that compared with Ag, carbon would exhibit a moderately higher reaction barrier and thus a slightly lower theoretical activity.

Beyond volcano analysis, another important advantage of Ag over carbon for mechanistic studies is its well-defined and uniform surface. Ag provides a relatively homogeneous surface with a narrow distribution of active sites. This allows us to apply d-band theory and the volcano relationship to rationally design and predict catalytic behavior, which is essential for establishing clear structure–activity relationships and systematically optimizing performance. In contrast, carbon materials are often heterogeneous with complex defect structures and oxygen-containing functional groups. While these features are valuable for certain electrocatalytic applications, they complicate the decoupling of different active sites and the direct application of d-band theory, which relies on a relatively uniform surface. We thank the reviewer again for this valuable suggestion, which has improved the generality and depth of our theoretical analysis.

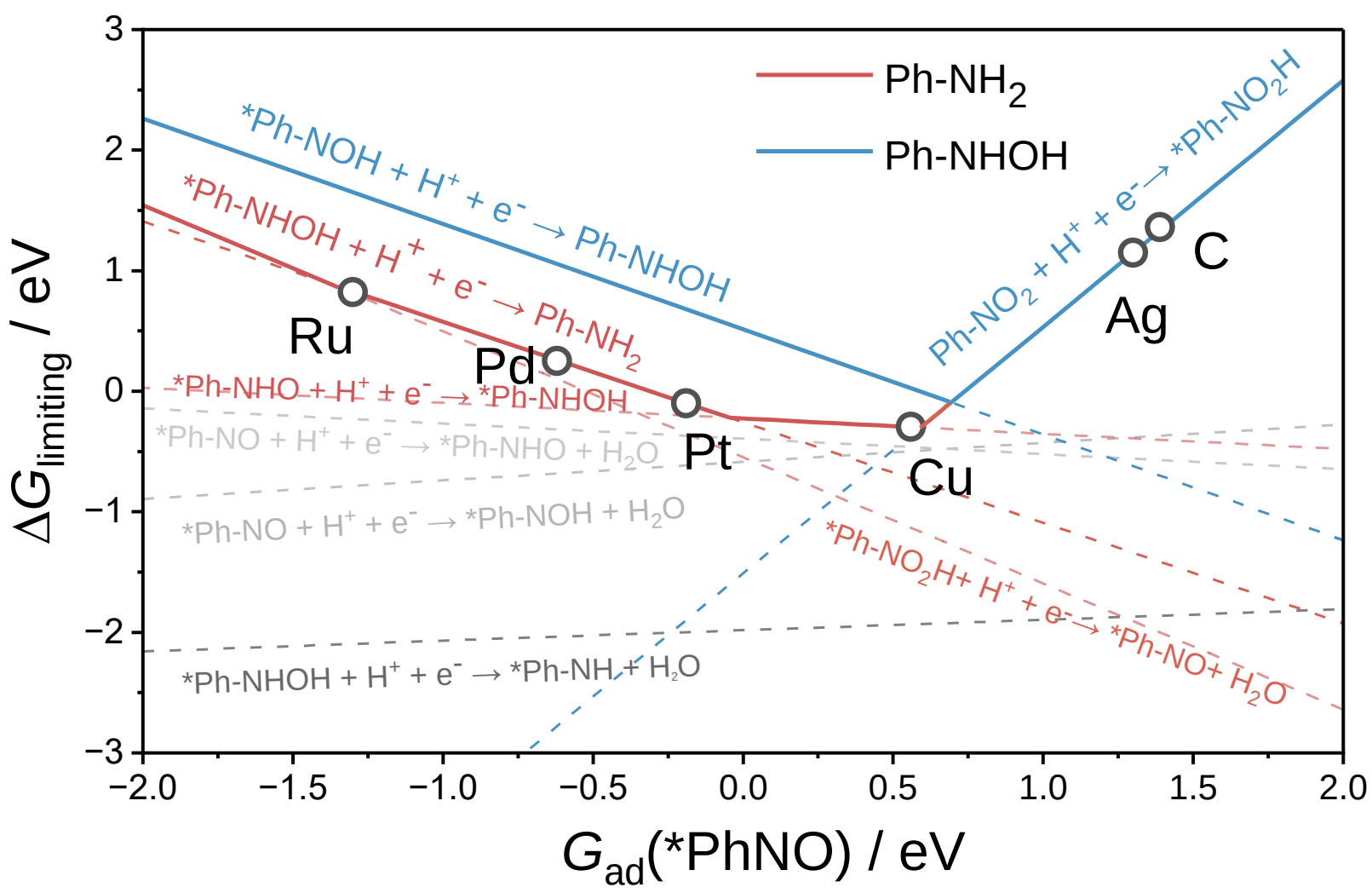


**Fig. R14** $G_{ad}$(*Ph-NO) plotted against $\Delta G_{limiting}$ over different cathodes from DFT calculations.

**Comment 4.** Figures showing CV or LSV data must have information in the caption to reveal the scan rate.

**Answer:** Thank you for your valuable comment. We have now added the scan rate information to the captions of all the figures that present the CV and LSV data. All the corresponding changes are marked in yellow in the revised manuscript and the revised SI.

**Comment 5.** in methods: please reveal what $Na_3C_6H_5O_7$ is.

**Answer:** Thank you for your comment. As suggested, we have clarified the chemical name of $Na_3C_6H_5O_7$ in the methods section. Specifically, $Na_3C_6H_5O_7$ is trisodium citrate.

**Comment 6.** in methods: the choice of pH 5.8 seems odd as this is where phosphate buffer has no buffer capacity?

**Answer:** Thank you for the comment. You are correct that phosphate buffer has a low buffering capacity at pH 5.8. In our experiments, however, the primary role of the phosphate solution was to maintain a constant weakly acidic condition and ionic strength, as the amount of acid and alkali generated by the reaction system itself is extremely minimal. To avoid misunderstanding, we have revised the Methods section by replacing "phosphate buffer (pH 5.8)" with "phosphate solution (pH 5.8)".

**Comment 7.** in methods: the Ag electrode was 1 cm x 1 cm, but were both sides active? Is the true geometric area twice this?

**Answer:** Thank you for the comment. We confirmed that the Ag electrode (1 cm × 1 cm) was fully immersed in the electrolyte and that both sides were active and participated in the electrochemical reaction. Therefore, the actual effective geometric area is 2 $cm^2$. Accordingly, we have revised the electrode description in the electrochemical measurements section. Furthermore, we have thoroughly rechecked all the electrochemical data normalized by the geometric area throughout the manuscript to ensure accuracy and consistency.

**Comment 8.** in the flow reactor a new type of cathode is introduced and apparently not characterised. How much silver was on the surface? Did the authors test just carbon cloth as cathode? It might work as well.

**Answer:** Thank you for the comment. The cathode used in the flow reactor is a carbon cloth-supported Ag catalyst prepared via electrodeposition. Following the reviewer's suggestion, we accurately quantified the Ag loading on the cathode via ICP–OES, with the measured loading being 0.488 mg $cm^{-2}$. We have also performed postelectrolysis characterization of the CV-Ag catalyst using SEM and XPS to comprehensively evaluate its structural and compositional stability (**Fig. R15**). The SEM images show that the original morphology of the catalyst was maintained, and the XPS results indicate that the characteristic Ag 3d binding energy peaks did not shift before and after electrolysis.

No additional peaks corresponding to $Ag^+$ were detected, indicating the chemical state of the Ag metal.

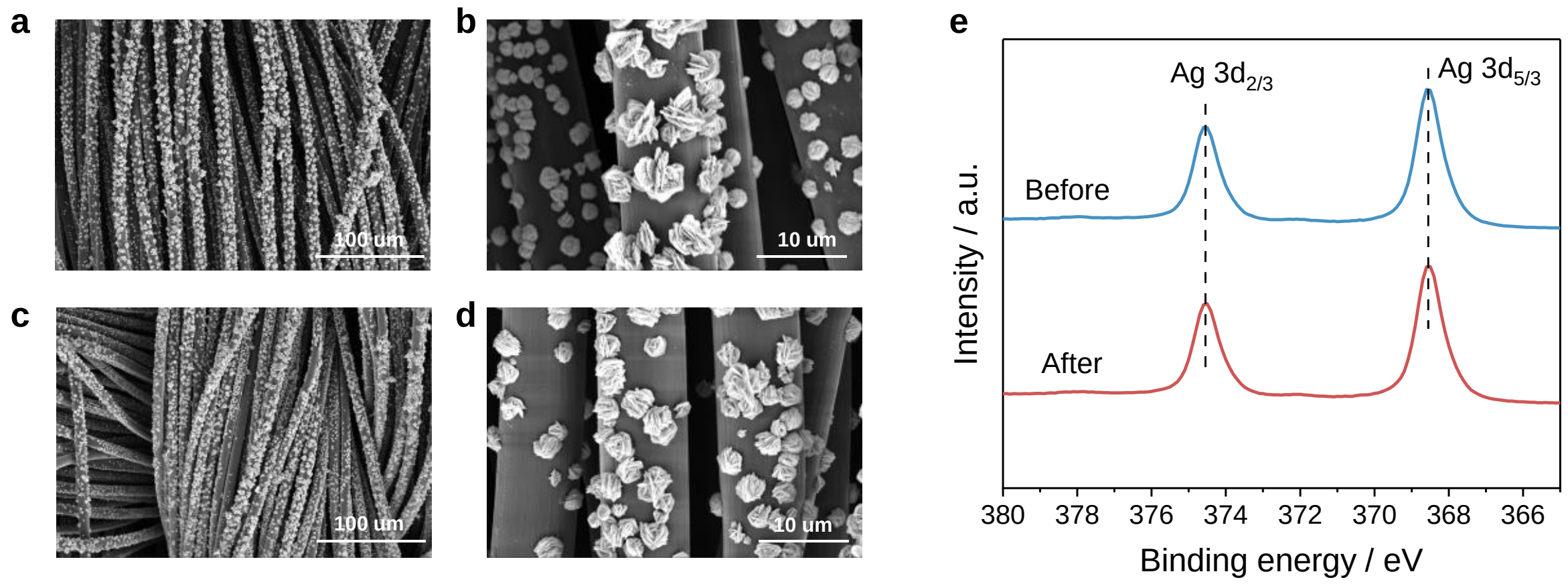


**Fig. R15 (Supplementary Fig. S42) a-b,** SEM images of CV-Ag deposited on carbon cloth. **c–d,** SEM images of CV-Ag after long-term electrolysis. **e,** XPS spectra of CV-Ag before and after long-term electrolysis.

Regarding the reviewer's question about pristine carbon cloth, we have tested it as a cathode under the same conditions. As shown in **Fig. R16**, the linear sweep voltammetry (LSV) curves of CV-Ag and carbon cloth in a flow MEA reactor reveal that compared with pristine carbon cloth, CV-Ag achieves a higher current and a lower overpotential. These results confirm that the deposited CV-Ag cathode provides a clear performance advantage.

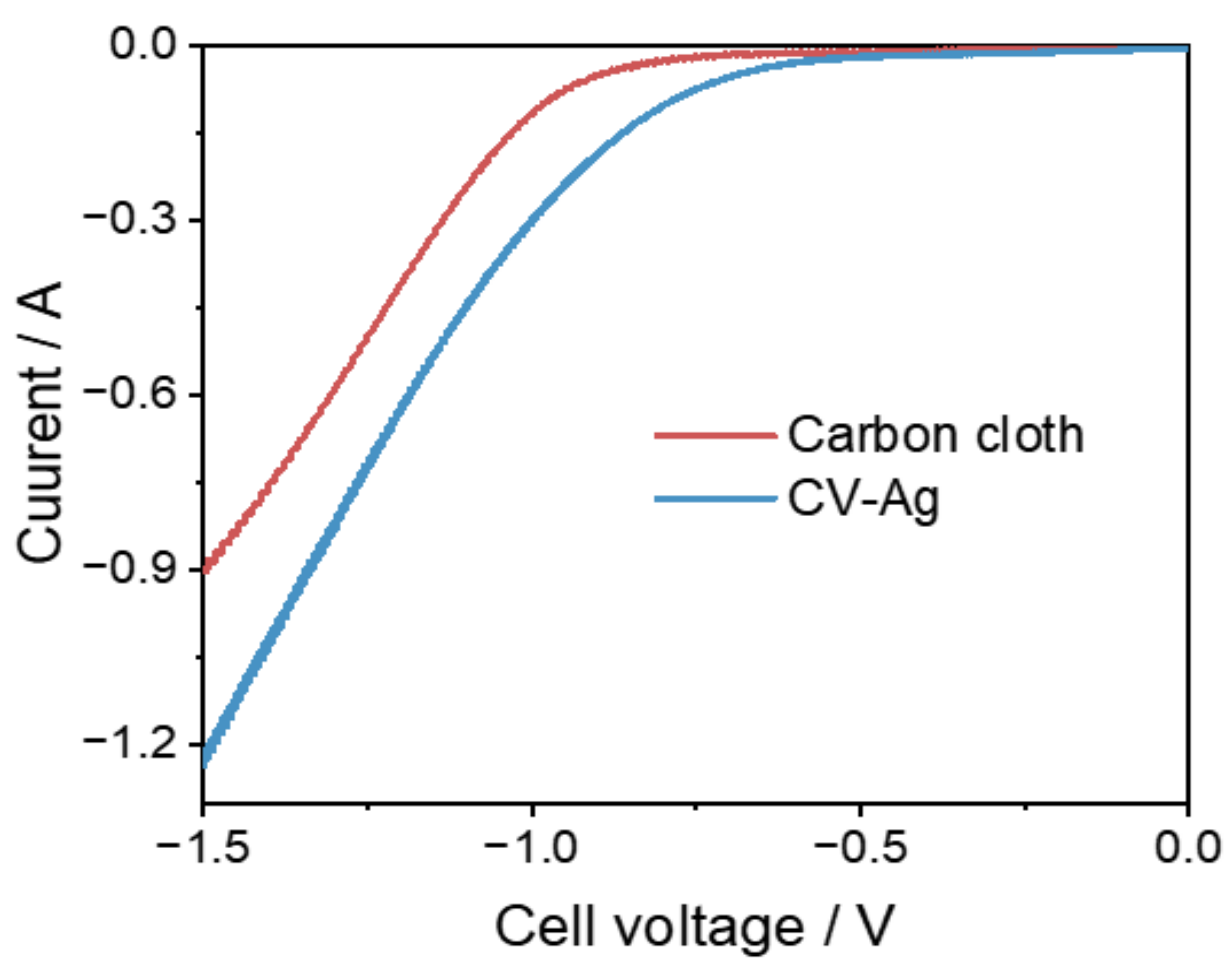


**Fig. R16** LSV curves of CV-Ag and carbon cloth in a flow MEA reactor.

**Comment 9.** in the flow reactor a new type of anode is introduced and not explained.

**Answer:** Thank you for the comment. The synthesis procedure of the carbon-cloth-supported iron single-atom catalyst (Fe-SAC)  was adapted from this reported method (***Nat. Commun.*** 2023, 14, 4210), and the obtained metal loading is consistent with that reported in the literature. Moreover, we performed XRD characterization to confirm the successful synthesis of Fe-SAC. The XRD pattern shows no characteristic diffraction peaks of crystalline Fe nanoparticles, indicating that Fe is atomically dispersed on the support (**Fig. R17**).

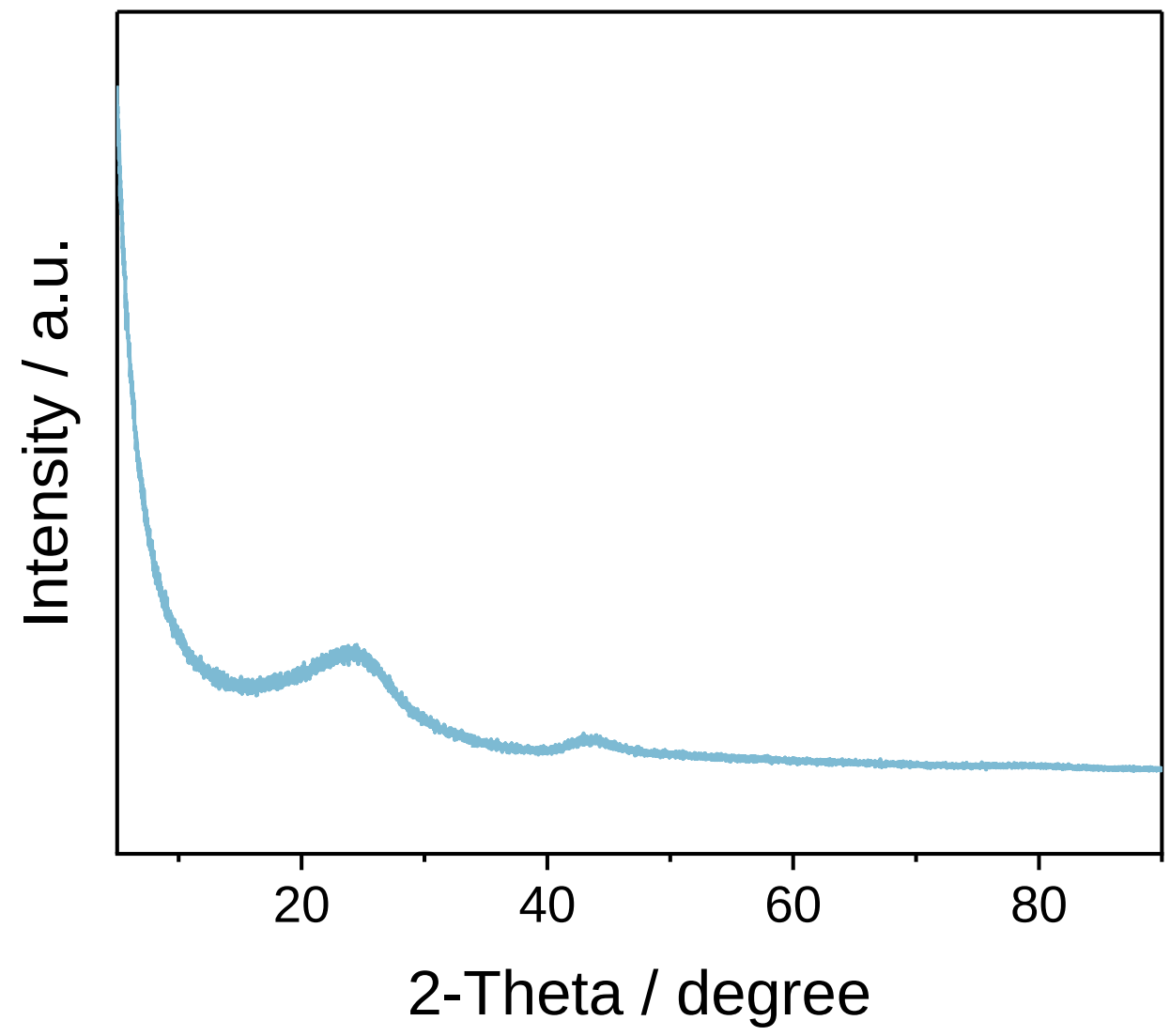


**Fig. R17** XRD pattern of Fe-SAC.

**Comment 10.** the use of dioxane as co-solvent is reported, but not clear. How much dioxane was necessary for the solubility of reagents/products?

**Answer:** Thank you for the comment. In our experiments, the volume ratio of the electrolyte to the cosolvent dioxane was 16:1 (v/v). Through repeated experimental optimization, at this ratio, 10 mM Ph-$NO_2$ and its product Ph-NHOH remained fully dissolved without any precipitation, and the presence of dioxane did not interfere with the electrochemical tests. A higher ratio of dioxane was avoided to minimize any potential influence on the reaction kinetics. We have added this detail to the revised SI.

**Comment 11.** Was the silver surface area determined quantitatively?

**Answer:** Thank you for the comment.  The Ag electrochemical surface area (ECSA) was quantitatively estimated using the double-layer capacitance ($C_{dl}$) method. This method provides a reliable relative

ECSA comparison among similar Ag-based electrodes because of the absence of well-defined H-UPD features on Ag in aqueous electrolytes (***ACS Catal.*** 2018, 8, 1234-1241). The complete test methods, calculation processes, and raw data are included in **Supplementary Fig. S25 and Supplementary Note 4**. In addition, we have added the corresponding discussion to the revised manuscript.

**Comment 12.** The choice of ascorbic acid seems odd. In fact, there is literature suggesting that ascorbate can reduce nitrobenzene even without any electrochemistry. In term of thermodynamics, electricity should be produced rather than consumed? It might be better for readers to be made aware of the fact that other cheaper sacrificial molecules could be used (or even better paired electrolysis could be developed).

**Answer:** Thank you for the comment. With respect to the nonelectrochemical reduction of Ph-$NO_2$ by ascorbic acid (AA), we are aware of those studies, which were conducted under strongly alkaline conditions where ascorbate is a strong reducing agent (Ref.). Under these acidic to near-neutral conditions in our system (pH 5.8), AA exists predominantly in its fully protonated form, which has a much lower reducing power. We conducted a blank control experiment under open-circuit conditions. The results show that the target Ph-NHOH product is not detected, confirming that the spontaneous reaction is negligible (**Fig. R18a**). Notably, a key point is that our electrolysis was performed in an MEA or an H-type cell, where the cathodic and anodic compartments are physically separated by a Nafion membrane. In this configuration, AA is confined to the anodic compartment and cannot directly contact the catholyte containing Ph-$NO_2$. Therefore, direct chemical reduction of Ph-$NO_2$ by AA is impossible in our system. We have clarified this point in the revised Experimental section.

With respect to the choice of AA and cheaper alternatives, we fully agree that AA is not a practical industrial reactant and was employed only as a proof-of-concept model reactant. The conceptual framework is transferable to low-cost alternatives such as glycerol (<1 USD/kg) or urea. Therefore, we are actively exploring paired electrolysis, where both compartments produce valuable products. In the revised manuscript, we have replaced the anodic reaction from AA oxidation with a glycerol electrooxidation reaction (**Fig. R18d–e**). Glycerol is a bulk byproduct of the biodiesel industry with wide sources and low cost. Moreover, glycerol can be directionally converted into high-value-added fine chemicals such as formic acid and lactic acid through electrooxidation, resulting in the construction of a paired electrolysis system with high-value products produced at both the cathode and anode. This fundamentally solves the cost problem of AA as a sacrificial agent and greatly improves the industrial application potential of the system. The relevant data have been organized and incorporated into the revised manuscript (**Revised Fig. 5 and Supplementary Fig. S25**).

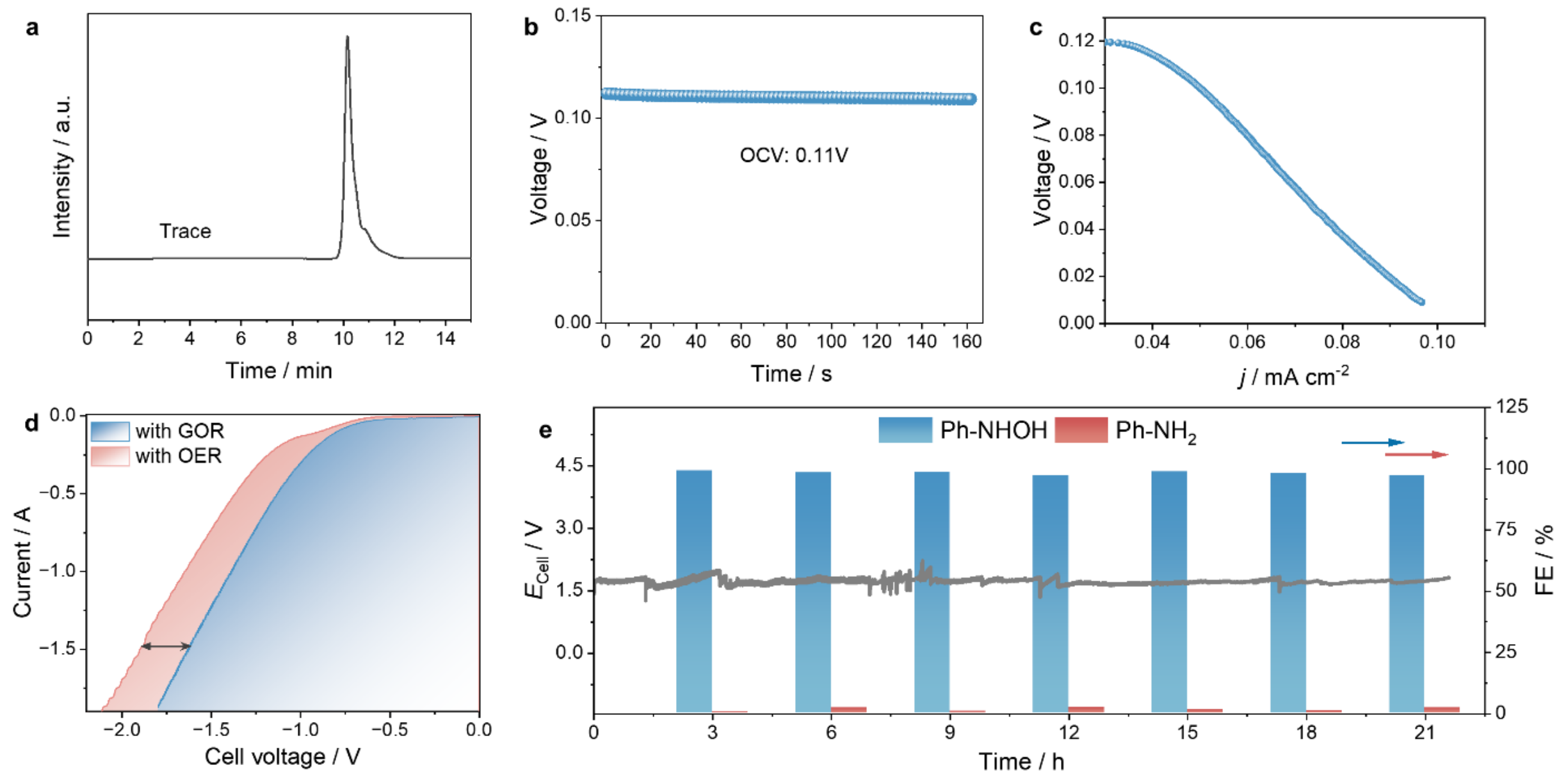


**Fig. R18 a,** Performance without electricity at the OCV. **b,** OCV curve. **c,** Discharge curve. **d,** LSV curves over the CV-Ag catalyst with or without GOR. **e,** Time-dependent voltage curve and FEs over CV-Ag at 1.5 A.

**Comment 13.** Would the presence of dioxane affect the adsorption energies on the silver??

**Answer:** Thank you for the comment. Previous work has demonstrated, through cyclic voltammetry and in situ spectroelectrochemistry, that dioxane does not undergo adsorption or redox reactions on gold, platinum, or glassy carbon electrodes within the common electrochemical window and can therefore be used as an inert solvent (***Electrochim. Acta*** 2017, 247, 1050-1056). Control experiments also revealed no difference in the Ph-NHOH FE with or without 1,4-dioxane under otherwise identical conditions (**Fig. R19**). Although dioxane does not interfere with catalysis, it is necessary for substrate solubility. Ph-$NO_2$ has limited solubility in pure water (approximately 2.0 g $L^{-1}$), and the addition of a small amount of dioxane (electrolyte: 1,4-dioxane = 16:1, v/v) significantly improves the solubility of Ph-$NO_2$ and the product Ph-NHOH, ensuring sufficient substrate concentration at the electrode surface for efficient electrolysis (***J. Electroanal. Chem.*** 1998, 453, 47-57). In summary, 1,4-dioxane acts as an inert cosolvent that is necessary for solubility but does not affect the catalytic surface. We have clarified this point in the revised manuscript.

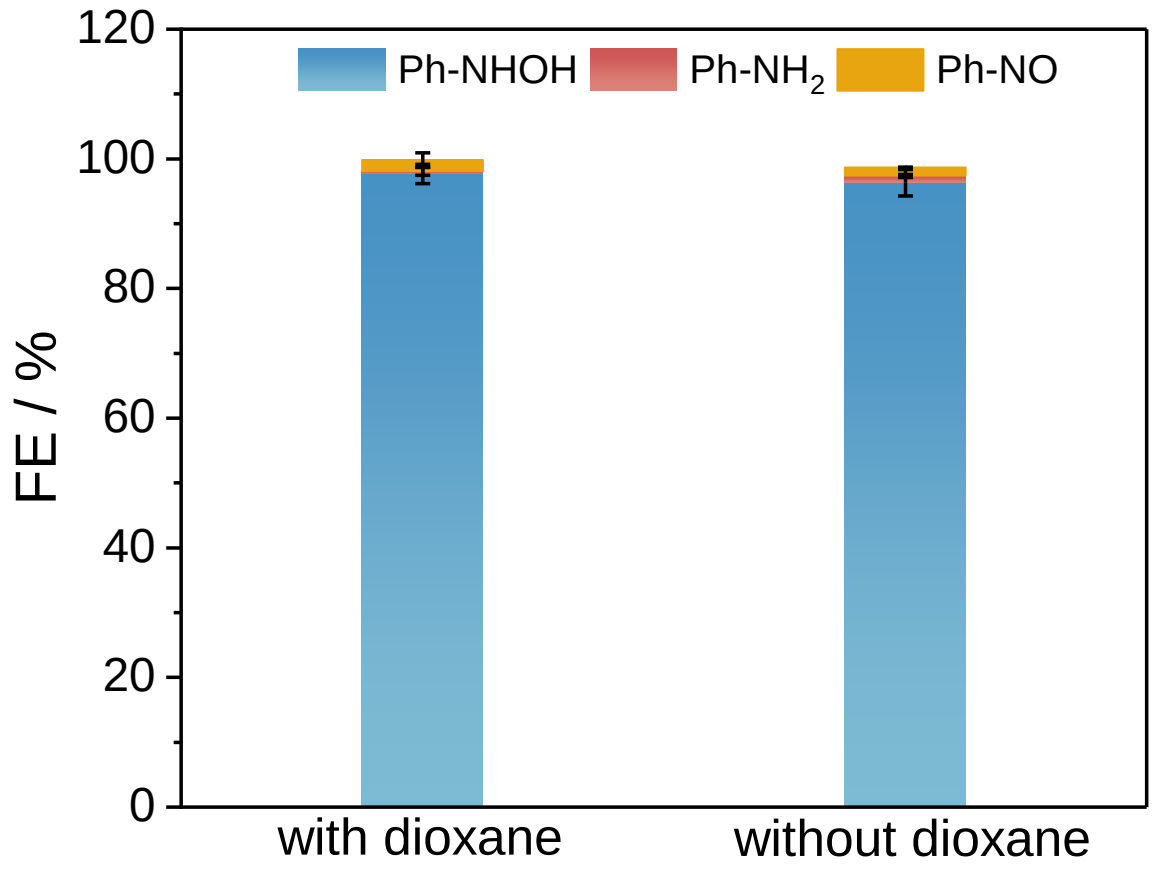


**Fig. R19** Comparison of performance with and without 1,4-dioxane

***We acknowledge all the kind comments and suggestions from the reviewers. We are sure that the quality of this work will be greatly improved according to these helpful comments and suggestions.***

# A point-by-point response to the reviewers

## To Reviewer 1:

### Reviewer Letter:

The authors have greatly revised the manuscript according to the comments from the Reviewers. It can be accepted for publication in its current version.

**Answer:** We highly appreciate the reviewer for the positive comments on our revised manuscript. We are sure that the quality of this work has been greatly improved. Thanks very much.

## To Reviewer 2:

### Reviewer Letter:

The author has properly addressed the comments. This reviewer recommends the publication of revised manuscript.

**Answer:** We sincerely thank the reviewer for the positive evaluation and the recommendation for publication. We greatly appreciate the valuable time and constructive suggestions that have helped strengthen this work.

## To Reviewer 3:

### Reviewer Letter:

The reviewer is satisfied with the revisions the authors have made. I recommend its publication in Nature Communications without changes.

**Answer:** We sincerely thank the reviewer for the highly positive assessment and the strong recommendation for publication in Nature Communications without further changes. We truly appreciate the time and expert insights devoted to evaluating our work.

## To Reviewer 4:

### Reviewer Letter:

The electrochemical reduction of nitrobenzene to N-phenylhydroxylamine is reported on different electrode types. The work is significant and the revision has improved the quality. Publication is now recommended.

**Answer:** We sincerely thank the reviewer for recognizing the significance of our work and for the positive recommendation for publication. We are very glad that the revisions have successfully improved the quality of the manuscript to meet with your approval.
